# KDM3A catalyses the oxidation of acetyl-lysine to hydroxyacetyl-lysine on histone H3K9

**DOI:** 10.1038/s41557-026-02112-x

**Published:** 2026-04-15

**Authors:** Roman Belle, John-Paul Bukowski, Rachel Schiller, Ronald Cutler, Eidarus Salah, Robert S. Dawber, Anthony Tumber, Joanna Bonnici, Jessica D. Kindrick, Loane Serrano, Patrick Rabe, Catrine Johansson, Marie-Hélène Ruchaud, Richard J. Hopkinson, William D. Figg, Sr, Paul E. Brennan, David R. Mole, Simone Sidoli, Akane Kawamura, Christopher J. Schofield

**Affiliations:** 1 Chemistry Research Laboratory, Department of Chemistry, University of Oxford, Oxford, UK; 2 Chemistry – School of Natural and Environmental Sciences, Newcastle University, Newcastle upon Tyne, UK; 3 Albert Einstein College of Medicine, Bronx, NY USA; 4 Nuffield Department of Medicine, University of Oxford, Oxford, UK; 5 Center for Cancer Research and National Cancer Institute, National Institutes of Health, Bethesda, MD USA; 6 Diamond Light Source, Didcot, UK; 7 Institute for Structural and Chemical Biology and School of Chemistry, University of Leicester, Leicester, UK; 8 Ineos Oxford Institute for Antimicrobial Research, Department of Chemistry, University of Oxford, Oxford, UK

**Keywords:** Enzymes, Chemical modification, Enzyme mechanisms, Post-translational modifications, Post-translational modifications

## Abstract

Histone modifications, including *N*^ε^-lysine acetylation and methylation, play critical roles in the regulation of eukaryotic transcription. The addition of acetyl and methyl groups and removal of acetyl groups to histones involve redox-neutral reactions. Demethylation is O_2_-dependent, as reported for reactions catalysed by the 2-oxoglutarate-dependent hypoxia-inducible factor (HIF) hydroxylases, one of which is structurally related to the Jumonji-C (JmjC) histone demethylases. We screened for substrates of the HIF-regulated JmjC lysine demethylase KDM3A and unexpectedly observed that purified recombinant KDM3A catalyses oxidation of the *N*^ε^-acetyl group of the Lys-9 of histone H3 (H3K9ac) giving an *N*^ε^-hydroxyacetylated product (H3K9acOH). Here we show that *N*^ε^-hydroxyacetyl-lysine is recognized by proteins known to bind to H3K9ac, including histone deacetylases and the YEATS domain-containing AF9. Studies employing an *N*^ε^-hydroxyacetyl-lysine selective antibody and mass spectrometry support the cellular relevance of *N*^ε^-hydroxyacetyl-lysine. Our combined biochemical and cellular results provide evidence for an unanticipated O_2_-mediated link between histone lysine *N*^ε^-acetylation and JmjC catalysis.

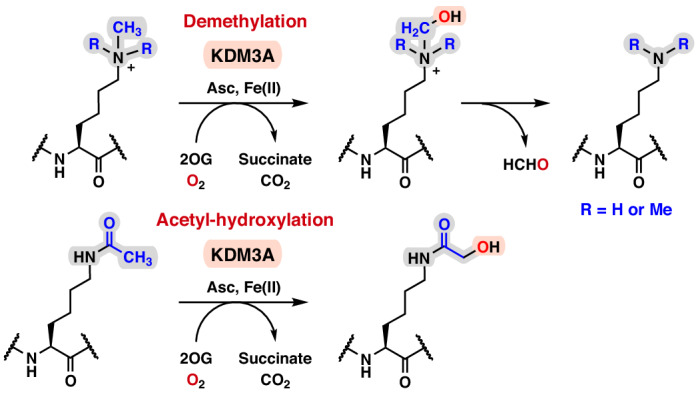

## Abstract (web summary)

Histone modifications, such as *N*^ε^-lysine acetylation and methylation, play critical roles in regulating eukaryotic transcription. Now, the oxidation of acetyl-lysine to hydroxyacetyl-lysine of a select histone has been identified as a distinct modification catalysed by the human JmjC histone demethylase KDM3A, which plays a role in the cellular hypoxic response.

## Main

**Fig. 1:**
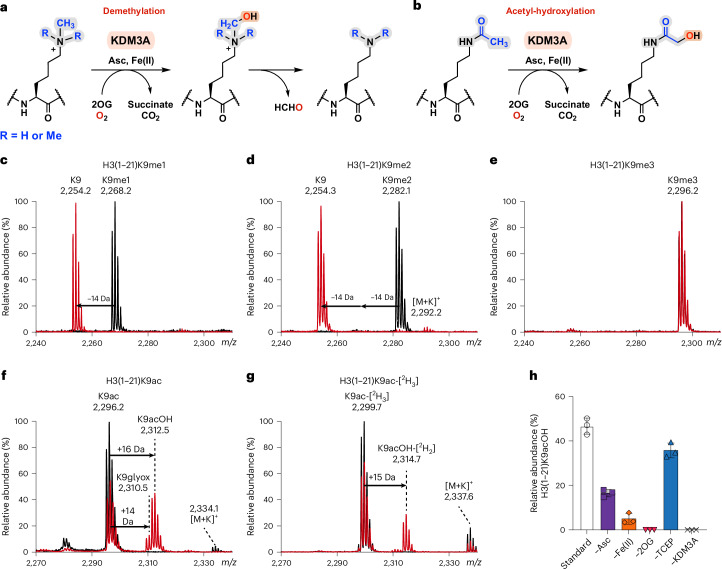
Screening of *N*^ε^-lysines-modified histone H3(1–21) peptides as KDM3A substrates. **a**,**b**, KDM3A-catalysed demethylation of *N*^ε^-methylated lysine 9 (mass shift: −14 Da) (**a**) and acetyl-hydroxylation of *N*^ε^-acetylated lysine 9 (mass shift: +16 Da) (**b**) on histone H3. Each two-electron oxidation is coupled to the conversion of O_2_/2OG to succinate/CO_2_. **c**–**h**, KDM3A was incubated with histone H3(1–21) peptides containing *N*^ε^-modified lysines and analysed by MALDI–TOF MS. **c**–**g**, Representative MS spectra of H3(1–21)K9me1 (**c**), H3(1–21)K9me2 (**d**), H3(1–21)K9me3 (**e**), H3(1–21)K9ac (**f**) and H3(1–21)K9ac-[^2^H_3_] (**g**). A −14-Da mass shift indicates loss of one methyl group; a +16-Da shift is consistent with hydroxylation. Black: *t* = 0 min; red: *t* = 60 min. **h**, KDM3A-catalysed hydroxylation of H3(1–21)K9ac in the absence of assay components. Conditions: KDM3A, 0.5 µM; histone peptide, 10 µM; ascorbate (Asc), 500 µM; Fe(II), 50 µM; 2OG, 100 µM; TCEP, 500 µM; 60 min (37 °C). Data are presented as mean ± s.d. (*n* = 3 independent assays). 2OG, 2-oxoglutarate; Asc, sodium l-ascorbate; TCEP, tris(2-carboxyethyl)-phosphine. Source data

Histone *N*^ε^-lysine acetylation (Kac) generally correlates with increased transcription and is dynamically regulated by histone acetyltransferases (HATs) and deacetylases (HDACs)^1,2^. In contrast, histone *N*^ε^-lysine methylation (Kme) is catalysed by methyltransferases (KMTs) and its effect on transcription is more context-dependent, including with respect to the position of the modified lysine residue and its methylation state^3,4^. Some histone lysine residues undergo multiple post-translational modifications (PTMs)—for example, lysine 9 on histone H3 (H3K9)^5^—where there is evidence for functional antagonism between *N*^ε^-lysine acetylation and methylation^6–8^. Despite longstanding evidence for lysine demethylation^9^, it was only relatively recently that two families of mechanistically distinct histone demethylases were identified: the flavin-dependent amine oxidases (KDM1s)^10^ and the JmjC domain containing histone demethylases (JmjC-KDMs), including the human KDM2–KDM7 subfamilies^11^. KDM1 and the JmjC-KDMs both demethylate mono-(me1) and di-(me2) *N*^ε^-methylated lysines, with some JmjC-KDMs acting on tri-methylated (me3) lysine^12^. The JmjC-KDMs are Fe(II)- and 2-oxoglutarate (2OG)-dependent oxygenases, which catalyse the O_2_-dependent hydroxylation of *N*^ε^-methyl groups to give a protein-bound hemiaminal intermediate that fragments to the demethylated product and formaldehyde^13^ (Fig. 1a).

The JmjC-KDMs are part of the JmjC 2OG oxygenase structural subfamily, other members of which catalyse the formation of stable alcohols on proteins (JmjC hydroxylases)^14^. A JmjC hydroxylase, factor-inhibiting hypoxia-inducible factor (FIH), has been shown to play a role in the hypoxic response in animals via catalysing hydroxylation of an asparagine residue on hypoxia-inducible factor (HIF) α-isoforms^15,16^. This modification hinders the interaction of α,β-HIF with histone acetyltransferases (CBP/p300), thus negatively regulating HIF-mediated transcription, which works to ameliorate the effects of hypoxia^15,16^. There is evidence that, like FIH and the 2OG-dependent HIF prolyl hydroxylases^17^, the cellular activities of some JmjC-KDMs are regulated by dioxygen (O_2_) availability^18–20^, and some JmjC-KDMs, in particular *KDM3A*, are HIF target genes that are upregulated in hypoxia^21^.

KDM3A and KDM3B catalyse the demethylation of di- and mono-methylated lysine 9 of histone H3 (H3K9me2/1)^22,23^; KDM3A is also reported to catalyse the demethylation of non-histone substrates^24,25^. In mice, *Kdm3a* is important in spermatogenesis, obesity resistance and sex determination^26–28^, and *Kdm3a*/*Kdm3b* have roles in embryonic stem cell (ESC) viability and embryogenesis^29^. KDM3A and KDM3B are involved in the development and maintenance of some cancers, including multiple myeloma^30^, renal cell carcinoma^31^ and prostate^32,33^ and colorectal cancers^34^; they are thus potential therapeutic targets^35^. *KDM3A* is a HIF target gene, including in multiple myeloma cells^30^, and regulates the expression of a subset of HIF target genes via a mechanism proposed to involve histone demethylation^34^. In this Article we report biochemical and cellular evidence for *N*^ε^-hydroxyacetyl-lysine (KacOH; Fig. 1b), an unprecedented histone PTM that is formed by KDM3A-catalysed hydroxylation of acetyl-lysine at H3K9. The results imply an unexpected direct O_2_-mediated link between histone lysine *N*^ε^-acetylation and JmjC-KDM catalysis.

## Results

### Isolated KDM3A catalyses the hydroxylation of H3K9ac

**Extended Data Fig. 1:**
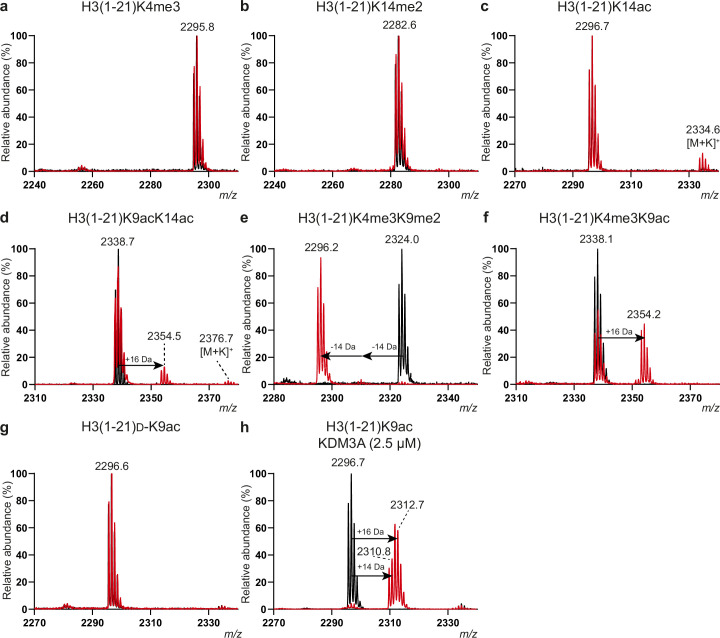
Screening of modified histone H3 *N*^ε^-lysines peptides as potential substrates for KDM3ACD using MALDI-TOF MS. Recombinant KDM3A_CD_ was incubated with modified histone H3 *N*^ε^-lysine peptides (residues 1-21). Reactions were analysed by MALDI-TOF MS. **a-h**, Peptide substrates used are given above representative MALDI-TOF MS spectra (n = 3 independent assays). A mass shift of −14 Da indicates removal of one methyl group; a + 16 Da shift indicates hydroxylation. Conditions: KDM3A_CD_ (**a-g**: 0.5 µM, **h**: 2.5 µM), peptide (10 µM), l-ascorbate (500 µM), Fe(II) (50 µM), 2OG (100 µM), and TCEP (500 µM) were reacted for 60 min (37 °C). Black t: 0 min, red t: 60 min.

Substrate screening studies have shown that, in addition to *N*^ε^-methyl lysine demethylation, some JmjC-KDMs also catalyse the formation of stable alcohol products^36^ as well as *N*^ω^-methylarginine demethylation^37–40^. Some JmjC protein hydroxylases, including FIH, are promiscuous, both in terms of their substrates and in the types of reaction they catalyse^14^. Given the roles of KDM3A and FIH in the HIF-mediated hypoxic response^34^, we considered it possible that KDM3A has unidentified substrates. To investigate this, a set of modified histone H3-derived peptide fragments were tested as potential substrates for the catalytic region of recombinant KDM3A (residues 515–1317, which includes its putative zinc finger and JmjC domain, KDM3A_CD_)^22^ (Supplementary Fig. 1 and Supplementary Table 1). As reported^41^, recombinant KDM3A_CD_ catalysed the demethylation of *N*^ε^-mono- and *N*^ε^-dimethyl-lysine of histone H3(1–21)K9me2/1 peptides, demonstrated by two −14-Da mass shifts observed in matrix-assisted laser desorption ionization-time of flight (MALDI–TOF) mass spectrometry (MS) assays (Fig. 1c,d). Demethylation was not observed for H3(1–21)K9me3, nor for other methylated lysines on H3 (H3K4, H3K14), as reported^41^ (Fig. 1e and Extended Data Fig. 1a,b).

**Extended Data Fig. 2:**
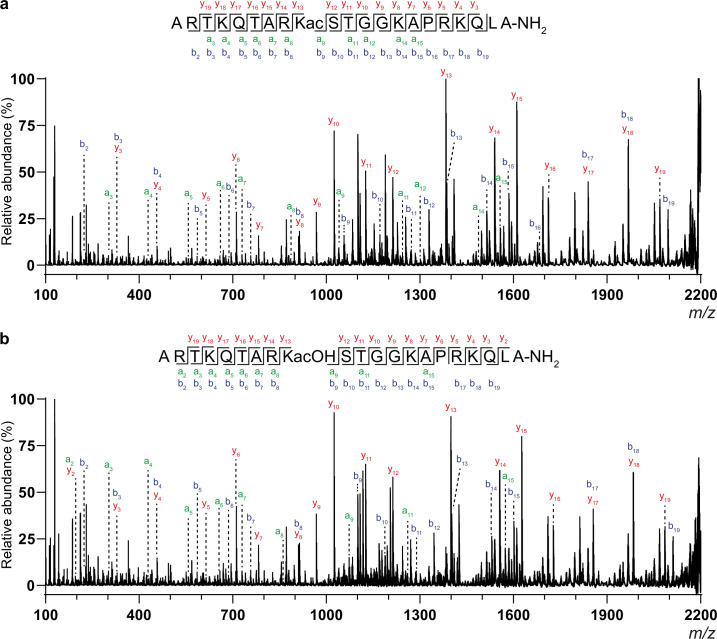
Identification of *N*^ε^-hydroxyacetyl-lysine 9 on histone H3 peptide from an KDM3A catalysed reaction using tandem MS (MS/MS). Recombinant KDM3A_CD_ was incubated with H3(1-21)K9ac for 0 min or 60 min; MS/MS analysis employed MALDI-TOF/TOF MS. MS/MS fragments were produced from the parent mass of: (**a**) H3(1-21)K9ac at 0 min, and (**b**) H3(1-21)K9acOH after 60 min incubation. Conditions: KDM3A_CD_ (0.5 µM), H3(1-21)K9ac (10 µM), l-ascorbate (500 µM), Fe(II) (50 µM), 2OG (100 µM), and TCEP (500 µM) were reacted for 60 min (37 °C). a-, b-, and y-ions are in green, blue, and red, respectively. Calculated and observed mass values are given in Supplementary Table 2.

**Extended Data Fig. 3:**
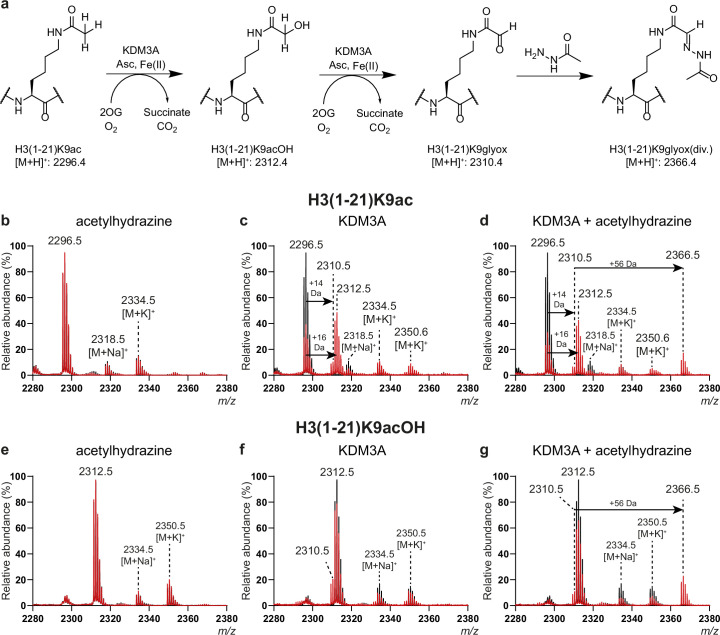
*N*^ε^-Acetyl-lysine and *N*^ε^-hydroxyacetyl-lysine modifications on histone H3 can be oxidized to *N*^ε^-glyoxylyl-lysine (K9glyox) which undergoes condensation with acetylhydrazine. **a**, KDM3A catalysed *N*^ε^-acetyl-lysine oxidation. Samples were quenched using EDTA solution after 60 min and where applicable, supplemented with acetyl-hydrazine solution, incubated for 60 min (37 °C), then analysed by MALDI-TOF MS. **b**, H3(1-21)K9ac incubated with acetyl-hydrazine without KDM3A; **c**, H3(1-21)K9ac incubated with KDM3A; **d**, H3(1-21)K9ac incubated with KDM3A, then acetyl-hydrazine; **e**, H3(1-21)K9acOH incubated with acetyl-hydrazine without KDM3A; **f**, H3(1-21)K9acOH incubated with KDM3A; and **g**, H3(1-21)K9acOH incubated with KDM3A, then with acetyl-hydrazine. Standard conditions: KDM3A (0.5 µM), peptide (10 µM), l-ascorbate (500 µM), Fe(II) (50 µM), 2OG (100 µM), and TCEP (500 µM) (60 min, 37 °C). For samples not containing acetyl-hydrazine or KDM3A, buffer was used. Black t: 0 min, red t: 120 min.

Unexpectedly, on testing *N*^ε^-acetylated histone H3 fragments, *N*^ε^-acetylated lysine 9 (H3K9ac) showed clear KDM3A_CD_-dependent conversion (~40%) to a product with a +16-Da mass shift, indicating potential oxidation to a hydroxyacetylated product (H3K9acOH) (Fig. 1f). By contrast, modification of H3(1–21)K14ac was not observed (Extended Data Fig. 1c). When both K9 and K14 were acetylated (H3(1–21)K9acK14ac), or when K4 was methylated (H3(1–21)K4me3K9ac), KDM3A_CD_ catalysed the formation of H3K9acOH (albeit at a reduced level for H3(1–21)K9acK14ac compared to H3(1–21)K9ac under the tested conditions), indicating potential for H3K9acOH formation on polyacetylated or methylated histone H3 (Extended Data Fig. 1d–f)^42,43^. H3(1–21)K9ac with a D-K9ac residue was not a KDM3A_CD_ substrate (Extended Data Fig. 1g). MS/MS fragmentation analyses demonstrated that the KDM3A_CD_ hydroxylation reaction occurs at H3K9ac (Extended Data Fig. 2 and Supplementary Table 2). Incubation with a tri-deuterated *N*^ε^-acetyl substrate, H3(1–21)K9ac-[^2^H_3_], resulted in a +15-Da mass shift, implying that hydroxylation occurs on the acetyl-lysine methyl group (Fig. 1g). We accrued evidence for further oxidation of *N*^ε^-hydroxylacetyl-lysine (H3(1–21)K9acOH) to *N*^ε^-glyoxylyl-lysine (H3(1–21)K9glyox) as demonstrated by the KDM3A_CD_-dependent formation of a +14-Da mass shift relative to H3(1–21)K9ac (Fig. 1f). Increasing the KDM3A_CD_ concentration promoted formation of H3(1–21)K9acOH and H3(1–21)K9glyox (Extended Data Fig. 1h), and derivatization with acetylhydrazine gave a +56-Da mass shift, supporting H3(1–21)K9glyox aldehyde formation (Extended Data Fig. 3a–d). Incubation of H3(1–21)K9acOH with KDM3A_CD_ also gave the H3(1–21)K9glyox product, supporting sequential oxidation to the alcohol, then aldehyde products (Extended Data Fig. 3e–g and Supplementary Fig. 2).

**Extended Data Fig. 4:**
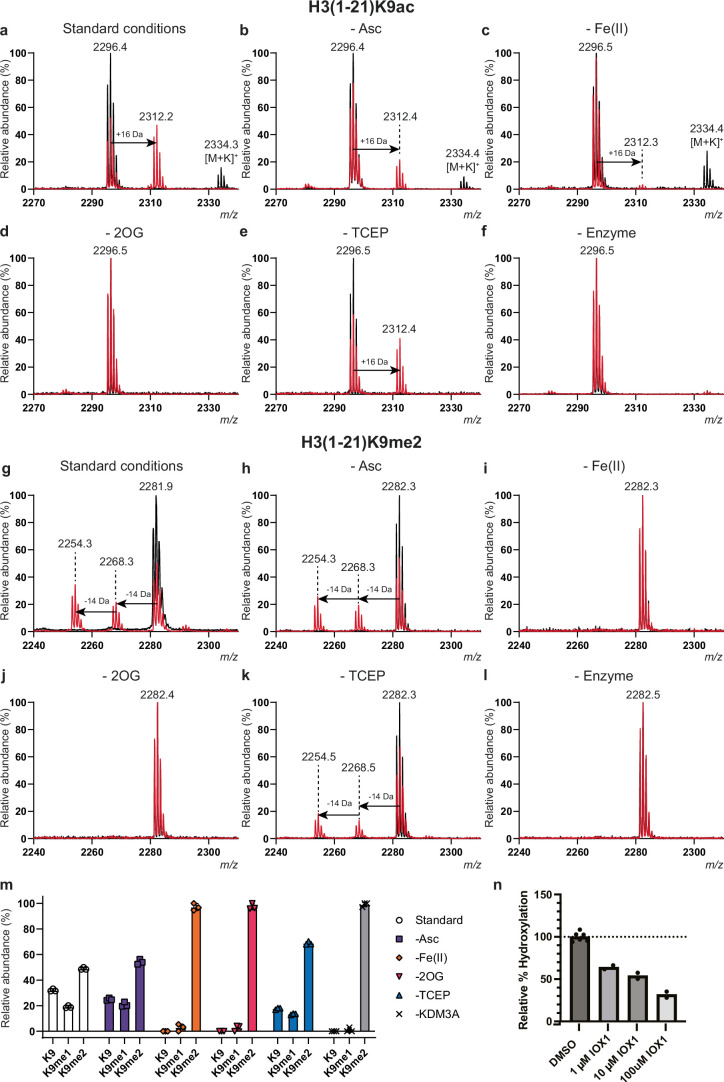
KDM3A catalysed hydroxylation of *N*^ε^-acetyl-lysine 9 or demethylation of *N*^ε^-dimethyl-lysine 9 on histone H3 peptides in the absence of an assay component, or in the presence of the inhibitor IOX1. KDM3A_CD_ reactions of H3(1-21)K9ac (10 µM) or H3(1-21)K9me_2_ (10 µM) under standard conditions, but with the absence of one component. **a,g**, standard conditions, **b,h**, in the absence of l-ascorbate, **c,i**, in the absence of Fe(II), **d,j**, in the absence of 2OG, **e,k**, in the absence of TCEP, and **f,l** in the absence of KDM3A_CD_. Representative MALDI-TOF MS spectra are shown for each peptide (n: 3 independent assays). **m** KDM3A_CD_ catalysed demethylation of H3(1-21)K9me2 in the absence of one assay component. The bar graph shows the relative abundance of substrate and demethylated products (mean ± SD, n = 3 independent assays). Conditions: l-ascorbate (500 µM), Fe(II) (50 µM), 2OG (100 µM), and TCEP (500 µM) were reacted for (60 min, 37 °C). Black t: 0 min, red t: 60 min. **n**, KDM3A_CD_ (0.5 µM) was pre-incubated (15 min) with DMSO or IOX1 (varied concentrations) before addition of H3(1-35)K9ac (5 µM), l-ascorbate (500 µM), Fe(II) (50 µM), 2OG (100 µM) and TCEP (500 µM). The reaction was quenched after 30 min (RT), then analysed by LC-MS. Hydroxylation activity is plotted relative to DMSO (%). Mean, n = 2 (IOX1) or 6 (DMSO) independent assays. Source data

**Extended Data Fig. 5:**
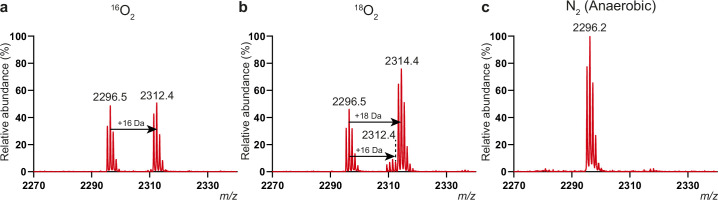
KDM3A catalysed hydroxylation of *N*^ε^-acetyl-lysine 9 under an atmosphere of ^16^O_2_, ^18^O_2_ or N_2_. Oxidation of the H3(1-21)K9ac to H3(1-21)K9acOH was observed under ^16^O_2_ (**a**) or ^18^O_2_ (**b**) atmospheres resulting in mass increases of +16 Da or +18 Da, respectively. No oxidation was observed under anaerobic conditions (N_2_, **c**). These findings demonstrate that O_2_ is required for *N*^ε^-acetyl group oxidation and that it is, at least substantially, the source of the oxygen atom incorporated into the hydroxylated product. Conditions: H3(1-21)K9ac (40 µM), KDM3A_CD_ (2.0 µM), sodium l-ascorbate (1.0 mM), Fe(II) (100 µM), 2OG (200 µM), TCEP (1.0 mM) for 120 min (red line) at 37 °C under ^16^O_2_ (**a**), ^18^O_2_ (**b**) or N_2_ (**c**) atmospheres.

As for the KDM3A_CD_-catalysed demethylation of H3(1–21)K9me2, oxidation of H3(1–21)K9ac was stimulated by Fe(II) addition and was dependent on 2OG (Fig. 1h and Extended Data Fig. 4). KDM3A inhibitors that compete with 2OG and complex Fe(II) will thus inhibit hydroxylation, as demonstrated by its inhibition with IOX1^44^ (Extended Data Fig. 4n). Incubation under an ^18^O_2_ atmosphere gave a product with high (>95%) incorporation of a single ^18^O atom, but no reaction occurred under an anaerobic atmosphere (N_2_), as observed for FIH and other 2OG-dependent protein hydroxylases (Extended Data Fig. 5)^45^. Comparison of the initial rates of demethylation of H3(1–21)K9me2 and hydroxylation of H3(1–21)K9ac showed that, at least with peptides, demethylation is preferred over hydroxylation (Supplementary Fig. 3). In competition studies with equimolar H3(1–21)K9me2 and H3(1–21)K9ac, a reduced H3(1–21)K9me2 demethylation rate was observed, an observation that implies that H3(1–21)K9ac inhibits KDM3A_CD_ demethylase activity (Supplementary Fig. 4). Further investigation using fluorescence intensity assays measuring formaldehyde production^46^ showed that H3(1–21)K9ac and H3(1–21)K9acOH manifest half-maximal inhibitory concentration (IC_50_) values of 23 µM and 34 µM for the demethylation of H3(1–21)K9me2. H3(1–21)K9, the product of H3(1–21)K9me2 demethylation, is also inhibitory (IC_50_ = 5 µM) (Supplementary Fig. 5), hindering more detailed kinetic evaluation.

Given the role of KDM3A in the HIF-mediated hypoxic response^34^, where the formation of stable alcohol products produced by 2OG oxygenase catalysis is crucial^47^, we considered the observation of KDM3A_CD_-catalysed H3(1–21)K9ac hydroxylation to be notable. We therefore investigated whether the ability of KDM3A_CD_ to hydroxylate H3K9ac is unusual by conducting studies with KDM3B, the catalytic domain of which has high sequence identity (∼83%) with KDM3A. KDM3B_CD_ (residues 882–1761) (Supplementary Fig. 1 and Supplementary Table 1) catalysed efficient demethylation of H3K9me2, but no evidence for H3(1–21)K9ac or H3(1–21)K9acK14ac hydroxylation, or H3(1–21)K9acOH oxidation, was observed (Supplementary Fig. 6). Altering the length of the histone peptide to either a shorter H3(1–15)K9ac or a longer H3(1–44)K9ac peptide did not lead to hydroxylation by KDM3B (Supplementary Fig. 7).

Because work with FIH and other 2OG-dependent hydroxylases has shown that full-length enzymes can impact catalytic efficiency, and folded proteins can be more efficient substrates than peptide fragments, in some cases giving a different reaction outcome^48^, we tested the combinations of full-length recombinant KDM3B_FL_ (which was highly active on H3(1–21)K9me2; Supplementary Fig. 6) with peptides and histone H3. We did not detect KDM3B_FL_-catalysed hydroxylation with H3(1–21)K9ac (Supplementary Fig. 6) or with recombinant intact histone H3.2K9ac (Supplementary Fig. 8); however, with both types of substrate, KDM3A_CD_ and KDM3A_FL_ showed hydroxylation activity. We also tested non-histone peptides containing sites of HIF1α acetylation as potential KDM3A_CD_ or KDM3B_CD_ substrates^42,49,50^—that is, HIF1α(522–542)K532ac, HIF1α(664-684)K674ac, and HIF1α(699–719)K709ac—but no evidence for hydroxylation was obtained (Supplementary Fig. 9).

**Extended Data Fig. 6:**
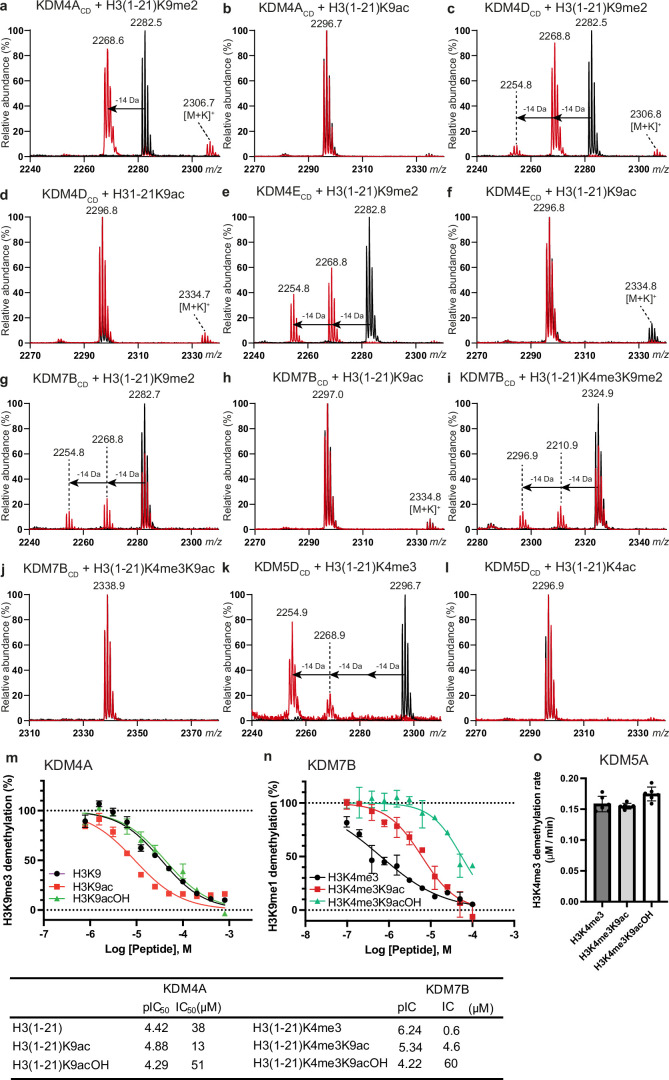
Hydroxylation of histone H3 *N*^ε^-acetyl-lysine 9 is catalysed by KDM3A, but not by related KDMs as determined by MALDI-TOF MS. KDM4A_CD_ (2.0 µM) (**a**,**b**), KDM4D_CD_ (2.0 µM) (**c**,**d**), KDM4E_CD_ (2.0 µM)(**e**,**f**), KDM7B_CD_ (2.0 µM) (**g-j**), or KDM5D_CD_ (2.0 µM) (**k,l**) were incubated with a known *N*^ε^-methylated peptide substrate and the corresponding *N*^ε^-acetylated peptide to test the latter as a potential substrate. **a,c,e,g**: H3(1-21)K9me_2_ (10 µM), **b,d,f,h**: H3(1-21)K9ac (10 µM), **i**: H3(1-21)K4me_3_K9me_2_ (10 µM), **j**: H3(1-21)K4me_3_K9ac (10 µM), **k**: H3(1-21)K4me_3_ (10 µM) or **l**: H3(1-21)K9ac (10 µM). Representative MALDI-TOF MS spectra are shown for each peptide (n = 3, independent assays). A mass shift of −14 Da indicates removal of one methyl group. Standard conditions for MALDI-TOF assays: l-ascorbate (500 µM), Fe(II) (50 µM), 2OG (100 µM), TCEP (500 µM) were reacted for 60 min (37 °C). Black t: 0 min, red t: 60 min. **(m)** KDM4A_CD_ (0.2 µM) or (**n**) KDM7B_CD_ (0.5 µM) were incubated with their corresponding methylated substrates (H3(1-21)K9me3, 10 µM for KDM4A, H3(1-21)K4me3K9me1, 4 µM for KDM7B) in the presence of varying concentrations of H3(1-21) or H3(1-21)K4me3 peptides containing K9, K9ac or K9acOH. The rate of demethylation was measured using the FDH based assay. (**o**) KDM5A_CD_ (0.5 µM) was incubated with H3(1-21)K4me3 containing K9, K9ac or K9acOH (1 µM) as a substrate. The rate of demethylation was measured using the FDH assay under standard conditions (mean ± SD, n = 2 biological assay repeats, 3 technical replicates per assay). Source data

Representatives of the JmjC-KDM4 subfamily that catalyse demethylation at H3K9 (Supplementary Fig. 1 and Supplementary Table 1) were tested for H3(1–21)K9ac hydroxylation activity. The recombinant demethylases KDM4A_CD_ (JMJD2A), KDM4D_CD_ (JMJD2D) and KDM4E_CD_ (JMJD2E)^51^ did not oxidize H3(1–21)K9ac, but did, as expected, efficiently catalyse the demethylation of H3(1–21)K9me2 (Extended Data Fig. 6a–f). KDM7B_CD_ (PHF8), which also acts at H3K9, catalysed demethylation of H3(1–21)K9me2 and H3(1–21)K4me3K9me2, as expected, but no evidence for H3(1–21)K9ac or H3(1–21)K4me3K9ac hydroxylation was found (Extended Data Fig. 6g–j). H3K9acOH, however, inhibited H3K9 demethylation as catalysed by KDM4A (IC_50_ = 51 µM) and KDM7B (IC_50_ = 60 µM), although less efficiently than H3K9ac (KDM4A IC_50_ = 13 µM and KDM7B IC_50_ = 4.6 µM) (Extended Data Fig. 6m,n). Similarly, KDM5D, which acts on H3K4, catalysed the demethylation of H3(1–21)K4me3, but did not catalyse H3(1–21)K4ac hydroxylation (Extended Data Fig. 6k,l). The presence of H3K9acOH/K9ac did not affect the rate of KDM5A-catalysed demethylation of H3K4me3 (Extended Data Fig. 6o).

### KDM3A and KDM3B hydroxylate H3K9ac on histones from cells

**Fig. 2:**
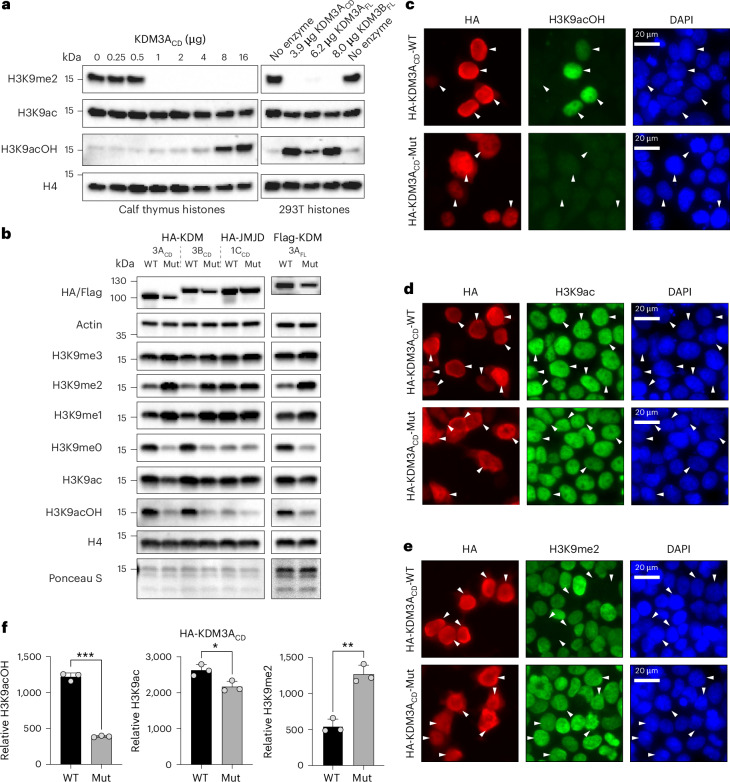
Evidence that KDM3A catalyses H3K9ac hydroxylation in cells. **a**, Western blots of calf thymus histones incubated with different concentrations of recombinant KDM3A_CD_ and of purified HEK293T histones incubated with equimolar KDM3A_CD_, KDM3A_FL_ or KDM3B_FL_, under standard assay conditions. Histones were analysed using PTM-specific antibodies, and anti-histone H4 was used as a loading control. **b**, Western blot of whole-cell lysate from HEK293T cells transiently transfected with KDM active (WT) or inactive (Mut) constructs: HA-tagged C-terminal regions (including C2HC4 and JmjC domain) of KDM3A, KDM3B and JMJD1C, and Flag-tagged full-length KDM3A. Cell lysates were analysed using histone PTM-specific antibodies, and anti-histone H4 and Ponceau S staining were used as loading controls. **c**–**e**, Immunofluorescence of HEK293T cells transfected with KDM active (WT) or inactive (Mut) HA-KDM3A_CD_-WT/Mut. Fixed HEK293T cells were incubated with HA and either H3K9acOH (**c**), H3K9ac (**d**) or H3K9me2 (**e**) antibodies, and co-stained with DAPI (nucleic acid). Scale bars are shown on the DAPI images. White arrowsheads indicate transfected HA-positive cells. Consistent with the western blot analysis, the HA signal of KDM3A Mut was weaker and more diffuse in the nucleus of transfected HEK293T cells, compared to KDM3A WT, an observation suggestive of reduced exogenous KDM3A Mut stability. **f**, Summary of immunofluorescence data (**c**–**e**) between transfected HA-KDM3A_CD_ WT or Mut for PTMs H3K9acOH, H3K9ac and H3K9me2. Data are presented as mean values ± s.d. of three biological replicates (*n* > 2,000 cells per replicate). Statistical significance was determined using a two-tailed *t*-test: H3K9acOH (*P* < 0.001), H3K9ac (*P* = 0.022), H3K9me2 (*P* = 0.001): *P ≤ 0.05; **P ≤ 0.01; ***P ≤ 0.001. Source data

**Extended Data Fig. 7:**
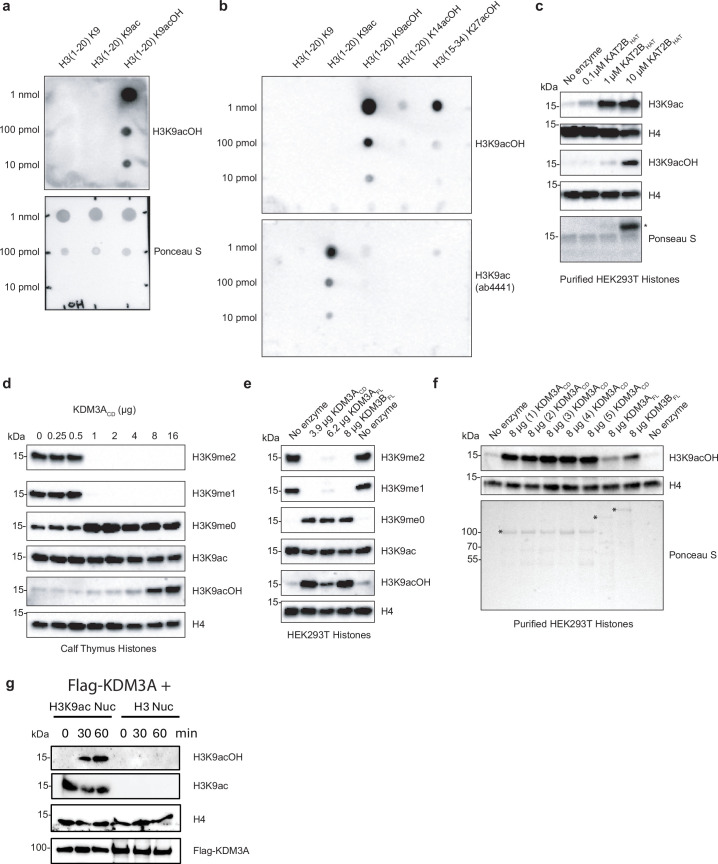
Characterisation of polyclonal anti-H3K9acOH antibody selectivity and western blot analysis. **a**,**b** Dot blots of unmodified-, *N*^ε^-acetyl- or *N*^ε^-hydroxyacetyl-lysine 9 histone H3 peptides incubated with commercial anti-H3K9ac (Abcam, ab4441) or custom anti-H3K9acOH. Ponceau S was used as a peptide loading control. **c** Western blot of purified HEK293T histones incubated with different concentrations of recombinant KAT2B_HAT_ (0-10 µM) under standard HAT assay conditions. Histones were analysed using anti-H3K9ac, anti-H3K9acOH, and anti-Histone H4 and Ponceau S were used as loading controls. The unmodified recombinant histone H3/H4 tetramer was used as a negative control. *Indicates recombinant KAT2B_HAT_. **d** Western blot of calf thymus histones incubated with increasing amounts of KDM3A_CD_ (0-16 µg). Histones were analysed using anti-H3K9me2, anti-H3K9me1, anti-H3K9me0, anti-H3K9ac, and anti-H3K9acOH antibodies. Anti-Histone H4 was used as a loading control. **e** Western blot of catalytic domain, full-length KDM3A and KDM3B incubated with histones from HEK293T cells. Analyses was performed using anti-H3K9me2, anti-H3K9me1, anti-H3K9me0, anti-H3K9ac, and anti-H3K9acOH. Anti-Histone H4 was used as a loading control. **f** Western blot of purified HEK293T histones incubated with different batches of purified recombinant KDM3A_CD_, and recombinant full-length KDM3A_FL_ and KDM3B_FL_. Histones were analysed using anti-H3K9acOH, and anti-Histone H4 was used as a loading control. Ponceau S was used to visualise the recombinant protein used in the assays. *Indicates purified recombinant proteins. **d,e** are supplementary to western blots in Fig. 2a. **g**. Evidence KDM3A catalyses the hydroxylation of H3K9ac on nucleosomes. Recombinant nucleosomes (Nuc, 1.7 µg), with (Active Motif: 81075) and without H3K9ac (Active Motif: 81070), were incubated with purified recombinant FLAG-tagged KDM3A_CD_ (1 µM) in the presence of Fe(II) (200 µM), ascorbate (500 µM) and 2OG (100 µM) in assay buffer [50 mM HEPES (pH7.5), 0.5 mM TCEP] for 0, 30, 60 min at 37 °C in 40 µL reaction. Western blots of nucleosome reactions were probed with anti-H3K9acOH, anti-H3K9ac, anti-H4 and anti-Flag antibodies. Sample loading was standardised to the H4 signal of H3K9ac containing nucleosomes. Source data

To investigate the biological relevance of KDM3A-catalysed H3K9ac oxidation, polyclonal antibodies selective for H3K9acOH were produced. Rabbits were immunized with synthetic H3K9acOH peptides coupled to carrier proteins, and the final sera were affinity-purified using a bead-coupled H3K9acOH peptide. Fraction selectivity was tested on peptides by dot blot analyses (Extended Data Fig. 7a,b). Importantly, the H3K9acOH antibody selectively recognized the H3K9acOH peptide rather than the unmodified H3K9, H3K9ac or H3K14acOH peptides. Modest cross-reactivity was observed with H3K27acOH, potentially due to a common ARKS motif in H3K9 and H3K27^52,53^. A commercial H3K9ac antibody did not detect H3K9acOH peptides, suggesting that these reagents can selectively distinguish between histone H3K9ac and H3K9acOH. Selectivity was investigated by western blots using purified HEK293T cell-derived histones incubated with recombinant lysine acetyltransferase KAT2B_HAT_ (PCAF, V493-E658; Supplementary Fig. 1 and Supplementary Table 1), an H3K9 and K14 acetyltransferase^54^ (Extended Data Fig. 7c). KAT2B_HAT_ treatment increased the H3K9ac signal^55^. Anti-H3K9acOH recognized histones incubated with KDM3A_CD_ (Fig. 2a), but did not substantially recognize histones acetylated with 1 µM KAT2B_HAT_; however, there was some recognition of histones incubated with 10 µM KAT2B_HAT_. Taken together, these results demonstrate that anti-H3K9acOH selectively recognizes H3K9acOH peptides and histones, but manifests some cross-reactivity with H3K27acOH and probably with high levels of H3K9 polyacetylated histones.

We titrated calf thymus histones with KDM3A_CD_ and performed western blot analyses (Fig. 2a). Increasing the KDM3A_CD_ concentrations decreased levels of the established H3K9me2/1 substrates, with a concomitant increase in unmodified H3K9 (Fig. 2a and Extended Data Fig. 7d)^56^. Higher KDM3A_CD_ concentrations correlated with increases in the H3K9acOH signal; the KDM3A_CD_ concentrations required were higher than for H3K9me2/1 demethylation, consistent with the activities observed with peptides (Fig. 1c,d,f and Supplementary Figs. 3 and 4). There was no increase in apparent H3K9ac levels with an increased concentration of recombinant KDM3A_CD_, providing evidence against anti-H3K9ac cross-reactivity for the increased H3K9acOH signal, under the tested conditions. Evidence for increased H3K9acOH levels was observed for histones extracted from HEK293T cells when incubated with five independently purified recombinant KDM3A_CD_ batches (Extended Data Fig. 7e,f) and with recombinant full-length human KDM3A_FL_ (Fig. 2a). Interestingly, studies with recombinant KDM3B_FL_ accrued evidence for H3K9acOH formation when incubated with isolated histones from HEK293T cells (Fig. 2a and Extended Data Fig. 7e). KDM3A_CD_ was also able to catalyse the hydroxylation of H3K9ac in nucleosomes, as demonstrated by western blot analyses showing evidence for an increase in the observed H3K9acOH signal when KDM3A_CD_ was incubated with recombinant nucleosomes containing H3K9ac; this was not observed with H3 unmodified nucleosomes (Extended Data Fig. 7g). In summary, the results using anti-H3K9acOH provide evidence for KDM3A-catalysed H3K9ac hydroxylation on calf thymus histones, purified HEK293T histones and recombinant nucleosomes. Under the tested conditions, hydroxylation of H3K9ac was less efficient than demethylation of H3K9me2/1.

### Overexpression of KDM3A/B increases H3K9acOH levels in cells

To further investigate KDM3-catalysed H3K9ac hydroxylation in cells, we overexpressed the three human KDM3 subfamily members in HEK293T cells by transfection of haemagglutinin and nuclear localization sequence (henceforth abbreviated as HA)-tagged C-terminal regions of catalytically active (wild-type, WT) or inactive (mutant, Mut) human KDM3A(511–1,321) and KDM3B(879–1,761), and the isozyme JMJD1C(1696–2540) (Supplementary Fig. 1 and Supplementary Table 3). Note that the catalytic activity, if any, of JMJD1C is unclear^23^. Consistent with previous reports, western blot analysis of lysates from HEK293T cells overexpressing the C-terminal regions of HA-KDM3A_CD_/3B_CD_-WT, but not HA-JMJD1C_CD_-WT, showed a robust reduction in H3K9me2/1 with a concomitant increase in H3K9^23,56^ (Fig. 2b). In agreement with our in vitro assays with histones, we observed an apparent increase in the H3K9acOH levels by western blots in HEK293T cells overexpressing the C-terminal regions of HA-KDM3A_CD_/HA-KDM3B_CD_-WT, but not with analogous catalytically inactive mutants. Increased H3K9acOH levels were also observed with overexpression of Flag-tagged full-length KDM3A WT (Flag-KDM3A_FL_-WT), albeit with a weaker H3K9acOH signal, possibly due to a lower transfection efficiency of the larger vector (Fig. 2b). A modest increase in global H3K9ac levels was observed with exogenous Flag-KDM3A/B_FL_-WT; this may potentially result from increased endogenous HAT activity on increased unmodified H3K9 resulting from exogenous KDM3A/3B demethylation of H3K9me2/1. This observation is analogous to the H3K27 poly-acetylation resulting from loss of Polycomb-mediated H3K27 methylation^7^. Note that the HA and Flag signals of the KDM3A/3B Mut proteins were consistently weaker than for KDM3A/3B WT, possibly because substitution of the HXD/EX···H motif destabilizes the core JmjC-fold by reducing Fe(II) binding and/or disrupting dimerization^57^.

To investigate whether H3K9ac hydroxylation is specific to KDM3A/B in cells, we transfected HEK293T cells with KDM7B and KDM4D. No evidence was found for Flag-KDM7B_CD_-WT activity producing K9acOH in HEK293T cells (Supplementary Fig. 10). Overexpression of Flag-KDM4D_FL_-WT reduced global H3K9me2 levels, which was comparable to cells overexpressing HA-KDM3A_CD_-WT, but only KDM3A overexpression increased H3K9acOH levels (Supplementary Fig. 10). Although further work is required, this observation suggests that formation of H3K9acOH in cells is KDM3A/3B-dependent.

**Extended Data Fig. 8:**
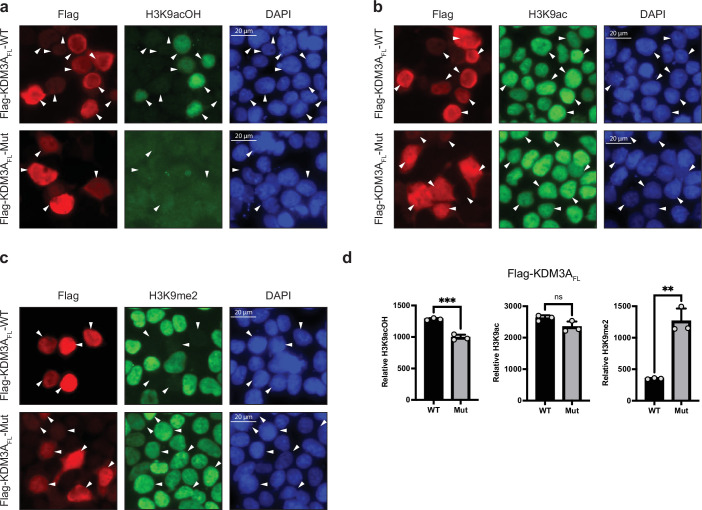
Heterologous full-length KDM3A expression increases H3K9acOH in HEK293T cells. **a**-**c**. Immunofluorescence of HEK293T cells transfected with catalytically active (WT) or inactive (Mut) Flag-tagged KDM3A. Fixed HEK293T cells were incubated with anti-Flag and either anti-H3K9acOH, anti-H3K9ac or anti-H3K9me2, and co-stained with DAPI. A scale bar is included on the DAPI image; white arrows indicate transfected Flag-positive cells. **d**. Summary of immunofluorescence data. Data represents the mean (across 3 wells) of the histone PTM in Flag-positive KDM3A WT / Mut transfected cells. Data are shown as mean values ± S.D of 3 biological replicates (n > 2000 cells per replicate). Statistical significance was determined using two tailed t-test: H3K9acOH (*P* < 0.001), H3K9ac (*P* = 0.064), H3K9me2 (*P* = 0.001): * = *P* ≤ 0.05; ** = *P* ≤ 0.01; and *** = *P* ≤ 0.001. Source data

We then used immunofluorescence assays to analyse heterologously expressed KDM3A activity on histones in single cells. HEK293T cells were transfected with HA-KDM3A_CD_-WT and Mut (H1120Y); at 24 h post-transfection, the cells were fixed, permeabilized, incubated with anti-HA, as well as anti-H3K9me2, anti-H3K9ac or anti-H3K9acOH, and co-stained with 2-(4-amidinophenyl)-1*H*-indole-6-carboxamidine (DAPI) (Fig. 2c–e). H3K9me2 was depleted in cells transfected with HA-KDM3A_CD_-WT, whereas H3K9acOH and H3K9ac levels were increased. There was no observed change in histone PTMs in cells transfected with HA-KDM3A_CD_-Mut compared to non-transfected cells. Similarly, immunofluorescence studies with Flag-KDM3A_FL_-WT and -Mut showed H3K9me2 was only depleted in cells transfected with Flag-KDM3A_FL_-WT; H3K9acOH levels increased, but to a lower extent than with the HA-KDM3A_CD_-WT (Fig. 2f and Extended Data Fig. 8). H3K9ac levels did not substantially change in Flag-KDM3A_FL_-WT transfected cells in comparison to Flag-KDM3A_FL_-Mut. These differences could be due to a combination of factors, including lower expression, reduced heterologously expressed full-length KDM3A stability, more efficient nuclear localization of the HA-tagged C-terminal region of KDM3A, or different protein interaction partners/target loci. In summary, consistent with the biochemical studies, cellular studies with anti-H3K9acOH provide evidence for the presence of H3K9acOH in bulk histones in HEK293T cells overexpressing either the C-terminal region or full-length KDM3A.

### HDAC inhibitor treatment in cells increases global H3K9acOH

**Fig. 3:**
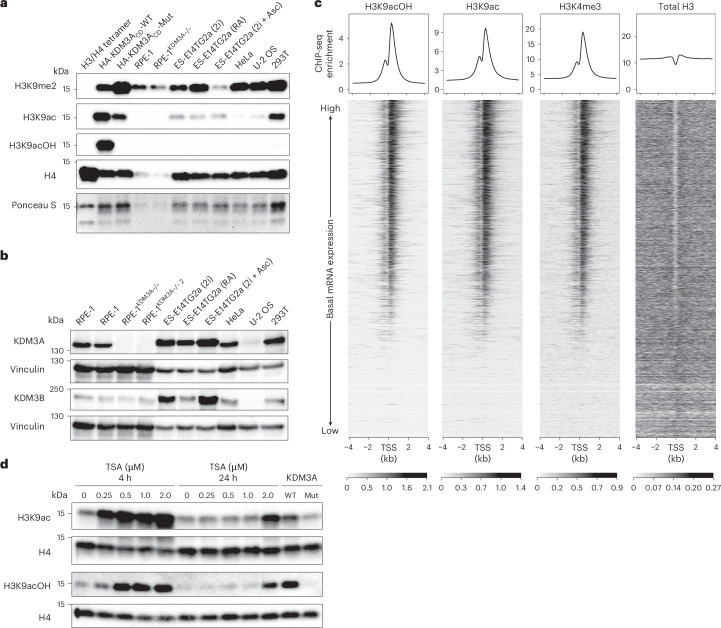
Characterization of H3K9acOH, KDM3A and KDM3B activities in mammalian cell lines. **a**, Western blot of purified histones and cell lysates from mammalian cell lines. **b**, Western blot of cell lysates from mammalian cell lines. **a**,**b**, RPE-1^(CrWT)^ has wild-type *KDM3A* and RPE-1^(Cr22.1)^ has a homozygous *KDM3A* deletion^58^. ES-E14TG2a cells were cultured either in 2i medium^59^, 1 µM retinoic acid (RA) (without 2i), or 2i medium + 345 µM l-ascorbic acid (2i + Asc) for 48 h. Recombinant histone H3/H4 tetramer and purified histones from HEK293T cells transfected with KDM active (WT) or inactive (Mut) HA-KDM3A_CD_-WT/Mut were used as controls. **c**, H3K9acOH co-localizes with H3K9ac and H3K4me3 at the TSSs of actively expressed genes. The average ChIP-seq signal enrichment of H3K9acOH, H3K9ac, H3K4me3 and total H3 in HEK293T cells treated with DMSO for 4 h is shown. ChIP-seq profiles were normalized to *Drosophila* spike-in DNA controls and plotted across the TSS ± 4 kb of all genes, ranked according to basal mRNA expression in HEK293T cells. H3K9acOH and H3K9ac co-localize at the TSS. **d**, Western blot of lysates from HEK293T cells treated with varied TSA concentrations (4 h and 24 h). HEK293T cells transfected with KDM active (WT) or inactive (Mut) HA-KDM3A_CD_-WT/Mut were used as controls. A reduction in K9ac is observed for TSA treatment after 24 h, which may, at least in part, be due to the short half-life of TSA^65^. In **a**,**b**,**d**, the purified histones and cell lysates were analysed using histone PTM-specific antibodies, anti-KDM3A anti-KDM3B; anti-histone H4 and anti-vinculin antibodies were used as loading controls. Source data

We performed western blot analysis on purified histones and lysates from mammalian cells with different endogenous *KDM3A*/*3B* expression levels, including non-transformed hTERT-immortalized RPE-1 cells with wild-type *KDM3A* or homozygous *KDM3A* deletion^58^, and mouse ES-E14TG2a ESCs (Fig. 3a,b). As both *Kdm3a*/*3b* are required for ESC viability, we analysed ES-E14TG2a cells grown in 2i medium^59^ and after retinoic acid-induced differentiation^28^. ES-E14TG2a cells were treated with vitamin C (l-ascorbate, Asc), as *Kdm3a*/*3b* mediate l-ascorbate induced loss of H3K9me2 (and probably H3K9me1) in ESCs^60^. As expected, we observed increased global H3K9me2 levels after retinoic acid addition^61^ and a decrease in H3K9me2 levels after l-ascorbate addition^60^; we did not, however, readily detect H3K9acOH under these conditions (Fig. 3a). Indeed, we did not detect substantial levels of H3K9acOH by western blots in any tested cell lines and conditions, including in hypoxia (Supplementary Fig. 10). Accordingly, we could not determine whether global H3K9acOH levels correlate with *KDM3A*/*3B* expression levels or other histone PTMs. These observations suggest that H3K9acOH, if present endogenously in the tested cell lines, is below the detection level with our H3K9acOH antibody, at least with bulk histone western blot analyses.

To investigate the potential genome-wide occupancy of H3K9acOH, we carried out a chromatin immunoprecipitation followed by sequencing (ChIP-seq) with H3K9acOH, H3K9ac and H3K4me3 antibodies in HEK293T cells. The average apparent H3K9acOH ChIP-seq signal was found to be localized around the promoter region at the transcriptional start site (TSS), with greater enrichment observed in the promoters of highly expressed genes (*r* = −0.78, *P* < 2.2 × 10^−16^ by Spearman rank correlation) (Fig. 3c). H3K9acOH enrichment patterns correlated with H3K9ac and H3K4me3, PTMs that are known to be associated with active chromatin^62,63^ (Fig. 3c).

**Extended Data Fig. 9:**
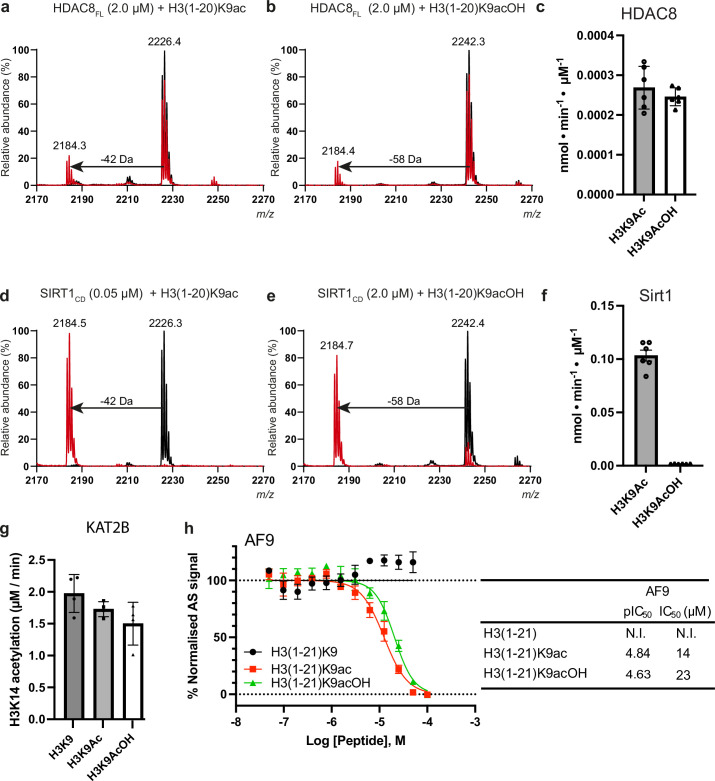
HDAC, HAT and YEATS domain activity on histone H3 on *N*^ε^-acetyl- and *N*^ε^-hydroxyacetyl-lysine 9 peptides. **a,b,d,e** Representative MALDI-TOF MS spectra of recombinant HDAC8_FL_ (**a,b** 2.0 µM) and SIRT1_CD_ (**d** 0.05 µM, **e** 2.0 µM) incubated with H3(1–20)K9ac (**a,c)** and H3(1–20)K9acOH. **b,d**. peptides. Mass shifts of –42 Da or –58 Da correspond to the removal of an *N*^ε^-acetyl or *N*^ε^-hydroxyacetyl-lysine 9 group. Representative spectra are shown. **c,f** Bar charts showing specific activities of recombinant human HDAC8_FL_ and SIRT1_CD_. Conditions: HDAC8_FL_ (2.0 µM) or SIRT1_CD_ (H3(1-20)K9ac: 0.05 µM, H3(1–20)K9acOH: 2.0 µM enzyme, peptide (10 µM) were incubated for 45 min (37 °C). Mean value ± S.D, n = 2 biological repeats with n = 3 technical repeats. Charge state of labelled ions: [M + H]^+^. Black t: 0 min, red t: 60 min. **g**. KAT2B_HAT_ (0.2 µM) was incubated with peptide substrate (20 µM) and AcCoA (50 µM) over 15 min (RT), with quenching of time points every 2 min. Activities were calculated as shown in the bar chart (n = 3 technical, n = 2 biological repeats; errors: mean ± S.D. **h**. H3(1-21)K9acOH peptide can displace biotin-H3(1-21)K9ac binding to the AF9 YEATS domain with similar potency to H3(1-21)K9ac as shown by an AlphaScreen displacement assay results, mean value ± S.D, n = 2 biological repeats with n = 3 technical repeats. Source data

Treatment of HEK293T cells with trichostatin A (TSA), a class I/II HDAC inhibitor^64^, increased both global H3K9ac and H3K9acOH levels in a dose- and time-dependent manner (Fig. 3d). TSA may indirectly increase H3K9acOH levels by promoting H3K9 hyperacetylation^65^, which is then hydroxylated to give H3K9acOH, as catalysed by endogenous KDM3A/3B. Alternatively or additionally, TSA may inhibit H3K9acOH hydrolysis by endogenous HDACs. To explore the latter possibility, we investigated human HDAC8 and SIRT1 (Supplementary Fig. 1c,d), which are functionally involved in the HIF-mediated hypoxic response^66,67^. Purified Zn(II)-dependent class I HDAC8_FL_ (M1-V377) had little or no substrate preference for H3(1–20)K9ac and H3(1–20)K9acOH peptides, whereas the class III HDAC SIRT1_CD_ (E82-S747) showed >40-fold reduction in the rate of deacylation of H3(1–20)K9acOH compared to H3(1–20)K9ac under the conditions tested (Extended Data Fig. 9). These observations are consistent with previous findings, with the yeast class III HDAC Hst2 (a Sir2 paralogue) being ~100-fold less active with H3K14acOH(9–19) compared to H3K14ac(9–19)^68^, whereas purified human HDAC8 has a preference for deacylating H3K14acOH over H3K14ac, as shown in studies with peptides^69^. The presence of H3K9acOH, however, did not affect the rate of KAT2B_HAT_-catalysed acetylation of H3K14 (Extended Data Fig. 9g). We explored whether H3K9acOH is recognized by AF9, an epigenetic mark ‘reader’ protein that recognizes Kac and lysine crotonylation^70,71^. In an AlphaScreen-based displacement assay, H3(1–20)K9acOH was able to bind to the purified AF9 YEATS domain, albeit with a lower affinity than H3(1–20)K9ac, (H3K9ac IC_50_ = 14 µM; H3K9acOH IC_50_ = 23 µM, Extended Data Fig. 9h). Although these results are with isolated proteins, our biochemical findings show the potential for KacOH engagement with a wide range of chromatin regulatory proteins, in a manner manifesting at least subtle differences compared to Kac.

### Characterization of KDM3A-induced H3K9acOH in HEK293T cells

**Fig. 4:**
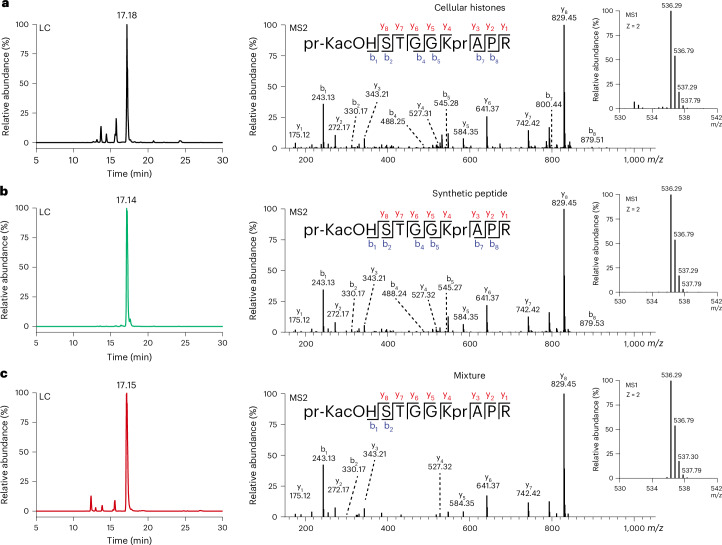
MS evidence for H3K9acOH in cell-extracted histones. Isolated histones from HEK293T cells transfected with HA-KDM3A_CD_-WT were propionylated, trypsin digested and analysed using HPLC-MS/MS. **a**–**c**, LC, MS1 and MS2 spectra show hydroxyacetylated (+58.009 Da) histone H3(9–17) peptide (pr-KacOH STGGKAPR) from cell extracts (**a**), synthetic peptide (**b**) and a mixture of cell extracts and synthetic peptide (**c**). The data shown are representative of three biological replicates.

**Extended Data Fig. 10:**
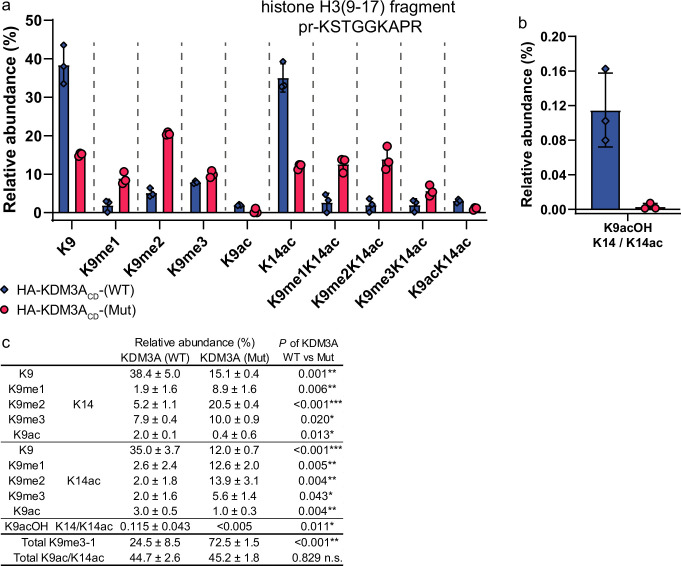
Relative abundances of modifications on histone H3(9-17) from HEK293T cells as observed by LCMS/MS. **a,b**, HEK293T cells were transfected with catalytically active (WT) or inactive (Mut) HA-KDM3A_CD_. Histones were extracted after 24 h and propionylated, then digested with trypsin; the fragmented histone peptides were analysed by LC-MS/MS. Note that all peptide masses contain N-terminal propionylation, and free lysines were propionylated. Relative abundances of extracted ions of histone H3 K9-R17 fragments with corresponding PTMs are displayed as a bar graph (**a**,**b**) with corresponding values tabulated (**c**). Values represent the percentage mean peak area of total displayed histone H3(K9-R17) peak area. Total K9acOH constitutes H3(K9-R17) fragments with K9acOH and with or without K14ac. Data is shown as mean values ± SD, n = 3 (KDM3A) biological repeats. Statistical significance was determined using two-tailed *t*-test: * = *P* ≤ 0.05; ** = *P* ≤ 0.01; and *** = *P* ≤ 0.001. Source data

To further investigate H3K9acOH formation in cells, histones isolated from HEK293T cells transfected with HA-KDM3A_CD_-WT/Mut were propionylated, then hydrolysed by trypsin digestion and analysed by MS/MS fragmentation following reported procedures (Fig. 4, Extended Data Fig. 10, Supplementary Fig. 11 and Supplementary Table 4)^72–74^. A mass corresponding to a histone H3(9–17) peptide fragment containing hydroxyacetyl-lysine at K9 (pr-KacOH STGGKprAPR-OH; +58.009-Da shift relative to the unmodified K9 peptide) was observed in the HA-KDM3A_CD_-WT sample using a targeted peptide search. This peptide manifested a chromatographic elution profile and MS/MS fragmentation patterns comparable to those observed for a synthetic (pr-KacOH STGGKprAPR-OH) peptide standard. A mixture of the cellular histone sample and the standard co-eluted with an identical MS/MS profile, supporting the presence and the identity of this PTM in cells (Fig. 4). Note that we did not accrue clear MS evidence for the presence of K9acOH-modified peptides in untreated HEK293T or HA-KDM3A_CD_-Mut cells and that analyses of PTMs on histone H3 may be complicated by additional modifications not identified through targeted peptide searches.

Qualitative analysis of MS/MS data for the relative abundance of H3(9–17) modifications indicates that most (~70%) of the peptides are mono-, di- or tri-methylated at H3K9 from HEK293T cells treated with the control dimethyl sulfoxide (DMSO; Extended Data Fig. 10a), in line with studies^5^. Consistent with the antibody studies, we observed reduced relative levels of H3K9me2 in HEK293T cells overexpressing HA-KDM3A_CD_-WT compared to HA-KDM3A_CD_-Mut, with a concomitant increase in unmodified H3K9 and H3K9ac levels in the former cells. Importantly, we observed evidence for the accumulation of H3K9acOH on histones purified from HEK293T cells overexpressing HA-KDM3A_CD_-WT, and the relative abundance change was consistent with antibody analyses on the purified histones (Extended Data Fig. 10b). The relative abundance of the doubly modified H3K9acOH/K14ac peptide was comparable to the singly modified H3K9acOH peptide (Extended Data Fig. 10b,c). Furthermore, there was also probably an indirect KDM3A_CD_-WT-mediated decrease in the relative abundance of H3K9me3. Interestingly, such trends in the changes to Kme/Kac levels have been observed on deletion of the H3K9me2/1 histone methyltransferase Kmt1c (G9a) in mouse ESCs^8,75^.

## Discussion

**Fig. 5:**
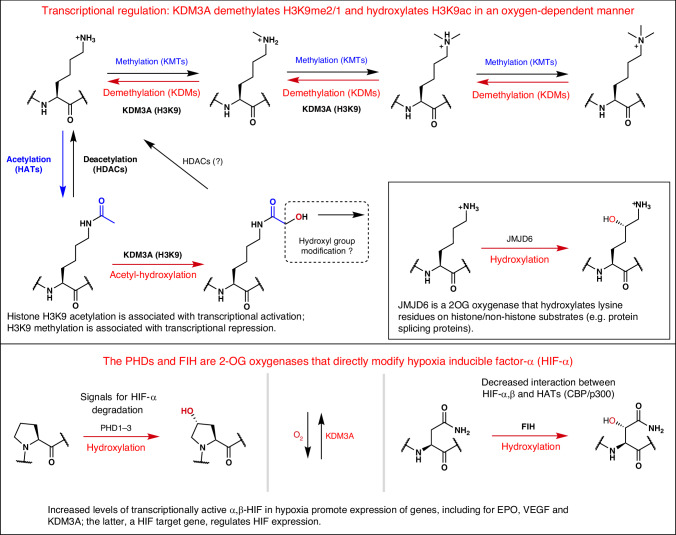
KDM3A-catalysed acetyl-hydroxylation links roles of 2-oxoglutarate oxygenases in the hypoxic response and histone modifications. KDM3A is a JmjC KDM acting on H3K9me1/2 and, as shown here, hydroxylates H3K9ac. KDM3A activity regulates HIF-αβ-enabled expression and *KDM3A* is a HIF target gene, so KDM3A levels rise in hypoxia. The JmjC subfamily 2OG oxygenase FIH catalyses HIF-α Asn-hydroxylation, causing reduced interaction of αβ-HIF with the histone acetyltransferases CBP/p300, so decreasing HIF-αβ promotes transcription. Catalysis by the HIF-α prolyl-hydroxylases (PHD1–3), which are not JmjC subfamily 2OG oxygenases, signals for HIF-α degradation in an O_2_-availability-limited manner. 2OG oxygenase reactions are shown in red, each of which is coupled to conversion of O_2_/2OG to succinate/CO_2_.

There is extensive evidence for functional links and competition between lysine *N*^ε^-acetylation and methylation at the same histone lysine residues. H3K9 acetylation is associated with transcriptional activity^62^, and H3K9 di- and tri-methylation correlates with transcriptional repression^3,63^. Lysine *N*^ε^-acetylation and methylation are reversed by HDACs and the O_2_ requiring JmjC KDMs, respectively. Our work provides evidence for an even more direct link between histone acetylation and O_2_, that is, that H3K9ac is a substrate for KDM3A, and potentially KDM3B, giving a chemically stable H3K9acOH product. The results are of interest in part because of the critical roles of KDM3A^34^ and histone acetylation in the HIF-mediated hypoxic response^15,16,68,69^, including with respect to the clinical relevance of HIF-2α upregulation in renal cell carcinoma/von Hippel–Lindau disease^76^, as well as the therapeutic upregulation of HIF-α for the treatment of anaemia and, potentially, acute myeloid leukaemia^77,78^ (Fig. 5). HDAC inhibitors, used for cancer treatment, may influence H3K9acOH levels by increasing H3K9ac availability for KDM3A-mediated hydroxylation.

Since the discovery of histone acetylation and methylation^79–81^, the number and type of identified histone PTMs have substantially increased, as enabled by the use of PTM-specific antibodies and high-resolution MS^5^. We are aware of the challenges in making definitive analytical assignments of PTMs, including oxygenase-catalysed hydroxylation in cells and in vivo, especially in regions subject to multiple PTMs, such as the N-terminal tail of histone H3^5,82,83^. Nonetheless, our cell-based studies, employing an H3K9acOH-selective antibody and MS-based proteomics, provide clear evidence for the presence of H3K9acOH, at least in cultured cells. Future work can explore the in vivo presence and roles of H3K9acOH in health and disease.

Cellular studies show that KDM3A, but apparently not KDM3B or JMJD1C, can be strongly upregulated by hypoxia in a HIF-mediated manner^21,30^. The expression of some HIF target genes is also regulated by KDM3A via a mechanism proposed to involve a reduction in H3K9 methylation in promoter regions of (some) HIF target genes^34^. Our studies with isolated histone fragments/histones and KDM3A provide definitive evidence for KDM3A-catalysed formation of H3K9acOH in a manner dependent on Fe(II), 2OG and O_2_ (Fig. 1). We did not acquire evidence for KDM3B-catalysed H3K9acOH formation in our work with peptides, but did so with full-length KDM3B and histones in studies with antibodies. Further validation of the H3K9acOH-forming activity of KDM3B is required, although, as precedented with work on some other JmjC oxygenases, the apparent discrepancy between biochemical and cellular studies may, at least in part, reflect different reactivities with different substrates^36,84^.

The high level of incorporation of an O_2_-derived oxygen atom in the hydroxylated H3K9acOH product is analogous to that observed with the HIF-α and other studied protein hydroxylases^45^. By contrast, in the case of KDM and arginine demethylase (RDM) catalysis, at least in the studied contexts^37,46,85^, the nascent hemiaminal intermediate is unstable, fragmenting to give a demethylated product; that is, the O_2_-derived histone ‘mark’ is lost as formaldehyde, which is subsequently detoxified by oxidation (a functional role for formaldehyde cannot be ruled out). Chemically stable protein hydroxylations, catalysed by the PHDs and FIH, play key roles in the HIF-mediated hypoxic response by regulating protein–protein interactions^14,45^. Thus, PHD-catalysed HIF-α prolyl hydroxylation substantially promotes binding of HIF-α with the von Hippel–Lindau protein/elongin B/C complex^86^, and FIH-catalysed HIF-α asparaginyl-hydroxylation is reported to reduce its interaction with the histone acetyltransferase CBP/p300^15,16^ (Fig. 5). Although we did not see global changes in H3K9acOH levels in hypoxia via western blots, given the chemical stability of H3K9acOH, exploring its role in relation to histone acetylation/deacetylation-mediated regulation of transcription during the hypoxic response, including at the individual gene level, is thus of considerable interest (Fig. 5).

The results identify H3K9acOH as a product of KDM3A catalysis, thus extending the known substrate selectivities of the JmjC enzymes and, more generally, 2OG oxygenases. Some JmjC-KDMs also have *N*^ω^-methylarginine-residue demethylation activity^37,40^ and with (likely) unnatural *N*^ε^-alkylated substrate analogues, they can catalyse the formation of stable hydroxylated products^36,37,84,87^. It therefore seems very likely that there are other JmjC 2OG oxygenase substrates and products to be discovered. Despite their nomenclature as lysine demethylases/KDMs, our work highlights the need to maintain an open mind regarding the reactions catalysed by the JmjC-KDMs.

It is possible that H3K9acOH is a precursor to other PTMs; for example, it could undergo further oxidation to aldehyde or acid products, a process similar to that observed with 2OG oxygenases acting on small molecules and nucleic acids such as 5-hydroxymethylcytosine^88^. Indeed, we observed evidence for the potential of KDM3A to catalyse the oxidation of H3K9acOH to an aldehyde. H3K9acOH also provides a handle for other types of PTM; for example, further acetylation or glycosylation are possible, with the latter precedented by C4 prolyl and C5 lysyl hydroxylations that enable glycosylations in non-histone proteins^89,90^. KDM3-catalysed H3K9acOH formation also has the potential to alter interactions of other chromatin-associated proteins acting on histones, for example, interactions with acetyl-lysine binders such as bromodomains or the YEATS domains^71^, as we have shown by studies with AF9. Although its biological relevance remains to be explored^68,69^, consistent with earlier reports, we found that H3K9acOH differentially affects catalysis by two hypoxia-linked HDACs; that is, SIRT1 activity is reduced with H3K9acOH compared to H3K9ac, while H3K9acOH was a substrate for HDAC8. These observations may be relevant with respect to HDAC inhibitor treatment (for example, TSA), where we observed effects on global H3K9ac and H3K9acOH levels. H3K9acOH also has the potential to indirectly influence the rate of demethylation by KDMs (directly at the H3K9 site as well as at neighbouring H3 sites) and H3 acetylation by HATs, including KAT2B, which catalyses lysine-acetylation of HIF1α^91^. Although our studies have focused on histone Kac hydroxylation, it is possible that non-histone Kac proteins, of which there are many^92^, are also substrates for KDM3A and/or other 2OG oxygenases, including in non-eukaryotic organisms.

The third member of the human KDM3 subfamily, JMJD1C, is required for male gametogenesis in mice^93^ and is of interest as a leukaemia and prostate cancer drug target^94,95^. JMJD1C has not been assigned as a KDM despite considerable sequence conservation with the catalytic domains of KDM3A/B. JMJD1C, however, has atypical active-site features (PDB 5FZO), and we did not find evidence that it catalyses H3K9acOH formation. One possibility is that JMJD1C has unusual co-substrate requirements, as supported by studies on 2OG oxygenases, including the JmjC hydroxylase FIH, showing that they can accept co-substrates other than 2OG^96^.

It is possible that KacOH is generated by enzymes other than KDM3A/KDM3B, including other 2OG oxygenases or acetyltransferase-catalysed reactions. In the latter regard, it is interesting that glycolic acid (HOCH_2_CO_2_H) is a component of the human diet as it is produced in all green plants from glycerate during photorespiration^97^ and could function as a source of KacOH. Finally, work towards studying oxygenase-catalysed production of hydroxyacetyl groups in non-protein biomolecules is also of interest. There is a striking example of this with the metallo-β-lactamase fold oxygenase cytidine monophosphate (CMP)-*N*-acetylneuraminic acid (Neu5Ac) hydroxylase (CMAH)^98^. CMAH catalyses hydroxylation of an *N*-acetyl group (CMP-Neu5Ac) to give *N*-glycolylneuraminic acid (Neu5Gc) in deuterostomes, but is absent in humans due to an inactivating mutation, the presence of which has been linked to altered susceptibility to infections^98^.

We hope that our results will encourage others to search for H3K9acOH and other hydroxyac(et)ylated lysine residues in different contexts, including at histone positions other than H3K9 and in non-histone proteins^99^, with in vivo studies clearly being of importance.

## Methods

### General materials and methods

The following chemicals and reagents were used. Ammonium iron(II) sulfate (09719-50 G), sodium l-ascorbic acid (95209 or 11140-50 G), l-ascorbic acid 2-phosphate (A8960), 2-oxoglutarate acid sodium salt (K2010), 2-oxoglutaric acid (75890-25 G), Ponceau S solution (P7170), retinoic acid (R2625), α-cyano-4-hydroxycinnamic acid (CHCA, 476870-10 G), piperidine in *N*,*N*-dimethylformamide (DMF; 20%, 80645-2L), acetic anhydride-^2^H_6_ (175641-1) and DMSO-^2^H_6_ were from Sigma-Aldrich. Fmoc-d-Lys(ac)-OH (A432506-1g) was from Ambeed Inc. Diisopropylethylamine (005027), pentafluorophenol (001354), ethyl 2-cyano-2-(hydroxyamino)acetate (043278), dicyclohexylcarbodiimide (128900) and *tert*-butoxyacetic acid (023523) were from Fluorochem. Tetrahydrofuran (10292182), acetonitrile (ACN) (10407440), formic acid (A117-50) and trifluoroacetic acid (TFA) (T/3258/PB05) were from Fisher Scientific. Fmoc-Lys(HCl)-OH was from Fluorochem (M03421) or Sigma-Aldrich (17290-5 G). Tris(2-carboxyethyl)phosphine hydrochloride (TCEP, BIT0122) and ß-nictotinamide adenine dinucleotide hydrate (NAD^+^, BIB3011) were from Apollo Scientific. Trichostatin A (SM36) was from Cell Guidance Systems, DMF (43465) from Alfa Aesar and Polysorbate 20 (233360010) from Acros Organics. TLC Silica gel 60 F254 plates were sourced from Merck.

NMR spectra were acquired using a Bruker 500-MHz (11.75 T) machine with 5-mm Norell NMR tubes. ^2^H-NMR spectra were ^1^H-decoupled and referenced to naturally abundant DMSO-^2^H_6_ (*δ* 2.50 ppm). Chemical shifts are in *δ* (ppm), coupling constants (*J*) in hertz and peaks are annotated as/or are combinations of broad (b), singlet (s), doublet (d), triplet (t), quartet (q) or multiplet (m). Low-resolution mass spectra were acquired in the negative or positive ion modes using an Agilent Infinity II 1260 UPLC+MSD XT machine equipped with a Thames Restek Raptor C18 column (2.7 µm, 100 × 3.0 mm). High-resolution small-molecule MS data were acquired with a Waters Acquity UPLC+Xevo G2-XS machine in negative or positive ion modes. Fmoc-amino-acid purification was achieved using a Biotage Selekt machine equipped with a Biotage Sfär C18 D–Duo 100-Å 30-µm column (CV: 45 ml, 25 ml min^−1^). Masses of peptides were analysed using MALDI–TOF Bruker rapifleX or LC-MS Agilent Infinity II 1260 UPLC+MSD XT machines with a Thames Restek Raptor C18 column (2.7 µm, 100 × 3.0 mm). Purities were determined using an Agilent Technologies 1220 Infinity LC machine with a Phenomenex bioZen 3-µm Peptide PS-C18 LC column (150 × 4.6 mm), with the following linear gradient employed: 0–3 min (2% (vol/vol) B), 3–16 (2–25% (vol/vol) B), 16–17 (25–98% (vol/vol) B), 17–19 (98%(vol/vol) B), 19–20 (98–2% (vol/vol) B), 20–21 (2% (vol/vol) B). Exceptions were as follows: HIF1α(699–719)K709t: 0–3 min (2–10% B), 3–20 (10–70% (vol/vol) B), 20–21 (70–98% (vol/vol) B), 21–23 (98% (vol/vol) B), 23–24 (98–2% (vol/vol) B), 24–25 (2% (vol/vol) B); HIF1α(522–542)K532ac: 0–3 min (2–5% (vol/vol) B), 3–20 (5–50% (vol/vol) B), 20–21 (50–98% (vol/vol) B), 21–23 (98% (vol/vol) B), 23–24 (98–2% (vol/vol) B), 24–25 (2% (vol/vol) B). Solvent A: 0.1% (vol/vol) TFA in water. Solvent B: 0.1% (vol/vol) TFA in acetonitrile. Greiner BIO-ONE, Microplate 384, black, F-bottom, non-binding, PS µClear plates were used for formaldehyde dehydrogenase (FDH) assays (Greiner #781906). MALDI–TOF assays were conducted using SW AlphaPlate-384 (PerkinElmer, 6008359) plates.

### Plasmids

DNA sequences encoding for human KDM3A (1–1,321 and 511–1,321 aa), human KDM3B (879–1,761 aa), human JMJD1C (1,696–2,540 aa of NCBI variant 1), human KDM4D (1–523 aa) and human KDM7B (1–489 aa of NCBI variant 2) were polymerase chain reaction (PCR)-amplified from plasmid DNA and inserted into the pCR8/GW/TOPO plasmid (ThermoFisher). DNA sequences encoding for the inactive mutants (KDM3A (H1120Y), KDM3B (H1560A and D1562A)) were amplified from plasmid DNA, and JMJD1C (H2336A and E2338A), KDM4D (H192A and E194A) and KDM7B (H247A and D249A) were generated by site-directed mutagenesis^100^. All entry clones were sequence-verified and transferred to a Gateway-compatible pCMV-HA-NLS or pcDNA5-1×Flag or 3×Flag destination vector using LR Clonase II (ThermoFisher).

### Antibodies

#### Antibodies from commercial sources

The antibodies used were actin (Sigma, A1978), Flag (Sigma, F1804), Flag (Sigma, F7425), HA (sc-7392), HA (CST, 3724S), HIF1α (BD Biosciences, 610959), H3K9ac (Abcam, ab4441, GR3290365-1, GR3229436-1, GR3253211-1, H3K9acOH fraction: R63, P1, F1.3 & F1.4, R43, P1, and F1.4, generated in this study), H3K9me3 (Abcam, ab8898), H3K9me2 (Abcam, ab1220), H3K9me1 (EpiCypher, 13-0014), H3K9me0 (Active Motif, 61399), histone H4 (Abcam, ab177840), KDM3A (Proteintech, 12835-1-AP), KDM3B (CST, 2621S), vinculin (Sigma, V9131), anti-rabbit immunoglobulin-G (IgG) (Vector, PI-1000-1) and anti-mouse IgG (Vector, PI-2000-1).

#### H3K9acOH polyclonal antibody production

H3K9acOH polyclonal antibodies were produced by DC Biosciences. In brief, two rabbits were immunized with both H3(5–13)K9acOH and H3(4–13)K9acOH peptides coupled to KLH and bovine serum albumin (BSA) carrier proteins (90-day protocol using Freund’s adjuvant). H3K9acOH-specific antibodies were affinity-purified from 20 ml of the final sera using the H3(4–13)K9acOH peptide coupled to agarose beads.

#### Cell lines

The cell lines used were as follows: HEK293T (ATCC, CRL-3216), HeLa (ATCC, CRM-CCL-2), RPE-1, RPE-1^(CrWT)^^58^, RPE-1^(Cr22.1)^ and RPE1^(Cr22.2)^^58^, and U-2 OS (ATCC, HTB-96). Cells were grown in Dulbecco’s modified Eagle medium (DMEM; Sigma, D6429) containing 10% fetal bovine serum (FBS; Sigma, F7524), 1% penicillin G-streptomycin (Sigma, P0781) and 1% l-glutamine (Sigma, G7513). ES-E14TG2a (ATCC, CRL1821) cells were cultured on dishes coated with 0.2% gelatin (Sigma, G1393) in serum-free 2i/LIF (2i) medium^59^ composed of 1:1 DMEM/F-12, GlutaMAX Supplement (ThermoFisher, 31331028) and neurobasal medium (ThermoFisher, 12348017), N-2 supplement (ThermoFisher, 17502048), B-27 supplement (ThermoFisher, 17504044), sodium pyruvate (ThermoFisher, 11360039), 2-mercaptoethanol (ThermoFisher, 31350010), MEM non-essential amino acids (ThermoFisher, 11140035), pen–strep (ThermoFisher, 15140122), GlutaMAX (ThermoFisher, 35050061), 3 μM GSK-3 inhibitor (Selleckchem, S2924), 2 μM MEK inhibitor (Selleckchem, S1036) and ESGRO recombinant mouse LIF (Millipore, ESG1106). ES-E14TG2a cells were split into fresh medium every two days using ESGRO Complete accutase (Millipore, SF006). ES-E14TG2a cells were treated with medium comprising 1 μM retinoic acid (RA; without 2i) or 2i medium + 345 μM l-ascorbic acid 2-phosphate (AA) for 48 h. HEK293T cells were treated with 0.04% DMSO (Sigma, D2650) or various concentrations of TSA (Cell Guidance Systems, SM36) for 4 or 24 h. Cell lines were regularly tested for the presence of *Mycoplasma* using a PCR-based method^101^.

#### Dot blot analyses

Lyophilized peptides were resuspended in H_2_O (Sigma, W4502) and quantified by NMR. Peptides were normalized by concentration and purity, then added directly to a nitrocellulose blotting membrane (Sigma, GE10600003). The membrane was blocked in phosphate buffered saline (PBS) with 0.05% (vol/vol) TWEEN 20 (PBS-T) and 5% (wt/vol) milk (Sigma, 70166), then incubated with histone PTM-specific antibodies diluted in PBS-T with 5% (wt/vol) BSA (Sigma, A7906). Peptides were detected using anti-rabbit or anti-mouse IgG in PBS-T and 5% (wt/vol) milk, chemiluminescent horseradish peroxidase (HRP) substrate (ThermoFisher, 34577) and the ChemiDoc MP Imaging System (Bio-Rad). Ponceau S was used as a loading control.

#### Cellular histone demethylase assays

HEK293T cells (~1.2 million) were seeded onto a 60-mm tissue culture dish. Next day, the cells were transfected with 6.5 µg of plasmid using a 3:1 ratio of FuGENE HD transfection reagent (Promega). The cells were collected at 48 h post-transfection and stored at −80 °C. Whole-cell lysates were analysed by western blotting.

#### Western blot assays

Frozen cell pellets were thawed and resuspended in TOPEX lysis buffer: 50 mM Tris pH 7.5, 300 mM NaCl, 0.5% Triton X-100, 1% sodium dodecyl sulfate (SDS), 1 mM dithiothreitol, protease inhibitor cocktail (Sigma, 05892791) and 33.3 U ml^−1^ benzonase nuclease (Sigma, 70746), then sonicated with ten cycles consisting of 30 s on/30 s off using a Bioruptor Pico instrument (Diagenode). Whole-cell lysate protein concentrations were determined using the BCA protein assay kit (ThermoFisher, 23227). Whole-cell lysate (10 µg), purified histones (1 µg) or recombinant H3/H4 tetramer (0.5 µg) were loaded onto a polyacrylamide gel (Bio-Rad, 4569036) and transferred onto a nitrocellulose blotting membrane (Sigma, GE10600003). For KDM3A/3B analyses, whole-cell lysates were loaded onto a 6% polyacrylamide gel. The membrane was blocked in PBS-T with 5% (wt/vol) milk (Sigma, 70166), then incubated with primary antibodies diluted in PBS-T with 5% (wt/vol) BSA (Sigma, A7906). Proteins were detected using anti-rabbit or anti-mouse IgG in PBS-T and 5% (wt/vol) milk, chemiluminescent HRP substrate (ThermoFisher, 34577) and a ChemiDoc MP imaging system (Bio-Rad). A PageRuler Plus Prestained Protein Ladder (ThermoFisher, 26619) was used for the sizing of proteins. After detection of histone PTMs, membranes were incubated with 1 mM sodium azide in 5% (wt/vol) milk for at least 3 h, then re-probed with anti-histone H4 as loading control. All western blot figures in this manuscript are representatives of least three independent biological replicates.

#### Formaldehyde dehydrogenase assays

Formaldehyde dehydrogenase assays were conducted as described in ref. ^102^. In brief, a peptide stock solution was diluted in assay buffer (Tween20 (0.01%), HEPES (50 mM) in MilliQ (pH 7.5, with adjustment using KOH) (12.5 µl)), followed by the addition of the KDM3A mixture (KDM3A, 0.6 µM; FDH, 2.0 µM; in buffer) (12.5 µl) in a 384-well plate black clear-bottom plate. The mixture was incubated for 10 min at room temperature. The peptide mixture (H3(1–21)K9me2 (20 µM) peptide, sodium l-ascorbate (1,000 µM), (NH_4_)_2_Fe(II)(SO_4_)_2_ (100 µM), 2-oxoglutaric acid (200 µM), β-nicotinamide adenine dinucleotide hydrate (500 µM) in buffer) (25 µl) was then added. The plate was subjected to orbital shaking for 15 s, then analysed using a BMG Labtech PHERAstar FSX (Firmware v. 1.30, software v. 5.41) machine equipped with an optical module (FI 350 460, 2106C1) every 30 s for 30 min at 25 °C. Data were processed using BMG Labtech MARS software (v. 3.32), analysed by Microsoft Excel and GraphPad Prism (v. 9.5.0) using the function log(inhibitor versus response – Variable slope) (four parameters). Images were produced using Adobe Illustrator CS6.

For KMD4A assays the following mixtures were used. KDM4A mixture: KDM4A (0.4 µM), FDH (0.5 µM) in buffer. Peptide mixture: H3(1–21)K9me3 (20 µM) peptide, sodium l-ascorbate (200 µM), (NH_4_)_2_Fe(II)(SO_4_)_2_ (20 µM), 2-oxoglutaric acid (200 µM), β-nicotinamide adenine dinucleotide hydrate (500 µM) in buffer. For KDM7B, the following mixtures were used. KDM7B mixture: KDM7B (2.0 µM), FDH (1.0 µM) in buffer. Peptide mixture: H3(1–21)K4me3K9me1 (4 µM) peptide, sodium l-ascorbate (1,000 µM), (NH_4_)_2_Fe(II)(SO_4_)_2_ (100 µM), 2-oxoglutaric acid (400 µM), β-nicotinamide adenine dinucleotide hydrate (500 µM) in buffer. Measurements were taken every 45 s for 30 min. The plate was analysed every 60 s for 79 min.

### Antibody-based detection of histone modifications

#### In vitro histone demethylase or hydroxylase assays

Histones (5 µg) from calf thymus (Sigma, H9250) or purified from HEK293T cells were incubated with different concentrations of recombinant KDM3A^(511–1317)^, KDM3A_FL_ (Active Motif, 31456), KDM3B_FL_ (Active Motif, 31429) in a 100-µl reaction containing 25 mM HEPES pH 7.5, 300 mM NaCl, 5% glycerol, 1 mM 2-oxoglutarate, 1 mM sodium l-ascorbate and 50 µM (NH_4_)_2_Fe(II)(SO_4_)_2_ for 2 h at 37 °C. For nucleosome assays, the following conditions were used: [H3K9ac-nucleosome] (Active Motif, 81075) (0.2 µM) and [H3K9-nucleosome] (Active Motif, 81070) (0.2 µM), l-ascorbate (500 µM) and 50 µM (NH_4_)_2_Fe(II)(SO_4_)_2_ (200 µM), 2-oxoglutarate (100 µM), incubated at 37 °C and quenched at appropriate timepoints using aqueous ethylenediaminetetraacetic acid (EDTA; 30 mM). Histones/nucleosomes (0.5 µg) were loaded onto a polyacrylamide gel (Bio-Rad, 4569036) and analysed by western blot using histone PTM-specific antibodies. Anti-histone H4 was used as a loading control.

#### In vitro KAT2B histone acetyltransferase assays

Purified histones (5 µg) from HEK293T cells were incubated with different concentrations of recombinant human KAT2B^(493–658)^ in a 100-µl reaction volume, containing 50 mM Tris pH 8.0, 150 mM NaCl, 10% glycerol, 0.1 mM EDTA, 20 µM acetyl-CoA (Sigma, A2056) and 1 mM TCEP for 2 h at 37 °C, then 0.5 µg of the histones were loaded onto a polyacrylamide gel (Bio-Rad, 4569036) and analysed by western blot using histone PTM-specific antibodies. Anti-histone H4 and Ponceau S were used as loading controls.

#### Cellular immunofluorescence assays

HEK293T cells (20,000) were seeded into the wells of a CellCarrier-96 black, optically clear-bottom, TC-treated microplate (Perkin Elmer), pre-coated with poly-l-lysine (Sigma, P4707) to improve cell adherence. Next day, 100 ng of plasmid DNA was transfected using a 3:1 ratio of FuGENE HD (Promega). The cells were fixed 24 h post-transfection, then stained. In brief, cells were fixed with paraformaldehyde solution 4% (vol/vol) in PBS (sc-281692), permeabilized with 0.5% Triton X-100 and blocked with 3% FBS (Sigma, F7524). The cells were incubated with anti-Flag or anti-HA, and anti-H3K9me2, anti-H3K9acOH or anti-H3K9ac, Alexa Fluor 594 (Thermo Fisher, A11032), Alexa Fluor 488 (Thermo Fisher, A11034) and co-stained with DAPI (Sigma, D9542). The cells were imaged and analysed using an Operetta high content imaging system and Harmony 3.5 software (Perkin Elmer).

#### ChIP-sequencing

Cells were grown on 15-cm plates to ~80–90% confluency on the day of collection. Chromatin-bound proteins were crosslinked to DNA by the addition of 37% formaldehyde to a concentration of 1% (wt/vol) followed by gentle rocking on ice (10 min). The reaction was quenched by the addition of glycine (125 mM) solution, followed by gentle rocking at room temperature (10 min). Cells were washed twice with PBS before being physically scraped into 5 ml of PBS. This suspension was centrifuged (4 °C, 800 r.p.m., 10 min). The supernatant was discarded and the pelleted cells were resuspended in 500 µl of ChIP SDS lysis buffer (1% SDS, 10 mM EDTA, 50 mM Tris pH 8.1, supplemented with Complete, EDTA-free protease inhibitor cocktail (Roche Diagnostics) (2× concentration) before incubation on ice (10 min). The lysates were diluted with ChIP dilution buffer (500 µl, 0.01% SDS, 1.1% Triton X-100, 1.2 mM EDTA, 16.7 mM Tris pH 8.1, 167 mM NaCl) and transferred to Bioruptor Plus TPX tubes suitable for sonication. Chromatin fragmentation was accomplished by 30 min of sonication using a Bioruptor Plus machine set to ‘HIGH’ mode (15 s on/15 s off). The sonicated chromatin was centrifuged (13,000 r.p.m., 4 °C, 10 min) and the supernatant collected. A 20-µl aliquot of sonicated chromatin was taken for quantification and the remainder was snap-frozen for storage at −80 °C. Fresh elution buffer (220 µl, 0.1 M NaHCO_3_, 1% SDS) was added to an aliquot, and NaCl solution (12.5 µl, 4 M) was added to each sample; the mixture was then shaken overnight (Eppendorf ThermoMixer F1.5, 65 °C, 1,400 r.p.m.). Proteins were digested with Proteinase K (Thermo Fisher, 2 µl) and shaken (1,400 r.p.m., 45 °C, 4 h). For RNA degradation, RNase A (Thermo Fisher, 1.0 µl) was added and the mixture was shaken (1,400 r.p.m., 37 °C, 30 min). Samples were then mixed with phosphate buffered (PB) binding buffer (Qiagen, 1.0 ml) and sodium acetate solution (10 µl, 3 M, pH 5.2). DNA was purified using MinElute PCR purification columns (Qiagen) and eluted with nuclease-free water (20 µl). Chromatin fragmentation was assessed by Agilent 2200 TapeStation System automated electrophoresis and D1000 DNA ScreenTape analysis, and ~200 bp was considered acceptable. The concentration of purified DNA was quantified using a Qubit 2.0 fluorometer (Invitrogen) and dsDNA Broad Range assay kit (Thermo Fisher). A predetermined amount of chromatin (as detailed in Supplementary Table 5) was taken from each sample and diluted with ChIP dilution buffer (0.01% SDS, 1.1% Triton X-100, 1.2 mM EDTA, 16.7 mM Tris, 167 mM NaCl, pH 8.1) to give a final volume of 1.0 ml in 2.0-ml Eppendorf DNA LoBind safe-lock microcentrifuge tubes (Thermo Fisher). This fixed amount of human chromatin was combined with the antibody of interest according to Supplementary Table 5. *Drosophila melanogaster* chromatin (Active Motif, 10 ng) and *Drosophila*-specific histone variant H2Av antibody (Active Motif, 1 µg) were added to each reaction as a spike-in control for downstream normalization. For ChIP-seq determining H3K9acOH, the *Drosophila* chromatin (5 ng) and antibody (0.5 µg) were reduced. These chromatin/antibody reactions were rotated overnight (4 °C), and antibody-bound DNA was separated using magnetic Protein G Dynabeads (Thermo Fisher). Beads (50 µl) were added to each sample along with ChIP dilution buffer (500 µl) and incubated on an end-over-end rotator (4 °C, 2 h). After the supernatant was removed, using a magnetic rack, samples were washed using low-salt wash buffer (500 µl, 0.1% SDS, 1% Triton X-100, 2 mM EDTA, 20 mM Tris pH 8.1, 150 mM NaCl), high-salt wash buffer (500 µl, 0.1% SDS, 1% Triton X-100, 2 mM EDTA, 20 mM Tris pH 8.1, 500 mM NaCl), LiCl wash buffer (500 µl, 1% Igepal, 1 mM EDTA, 10 mM Tris, 250 mM LiCl, 1% sodium deoxycholate, pH 8.1) and two rounds of TE wash (500 µl, 10 mM, 1 mM EDTA, Tris pH 8.0) using a sample rotary machine (4 °C, 5 min). Fresh elution buffer was added to the beads (120 µl) and shaken (1,400 r.p.m., room temperature, 15 min). The supernatant was collected, the elution process was repeated, and the eluates were pooled. The final immunoprecipitated (IP) material and input controls were de-crosslinked and purified as described above. All libraries were prepared and sequenced (single-end) by the US NCI CCR Genomics Core following Illumina recommended protocols for the NextSeq 500 75 cycle high-output platform.

#### Analysis of ChIP-seq data

ChIP-seq reads were aligned to the *D. melanogaster* BDGP6 genome with BWA (0.7.5a-r405). The output SAM files were converted to BAM using SAMtools view (0.1.19), and the *Drosophila* mapped reads were counted with SAMtools flagstat for later normalization^103^. *Drosophila* reads were filtered out and BAM files sorted with SAMtools before alignment to the human GRCh37 genome. Human mapped files were converted to BAM, counted with SAMtools flagstat, filtered with SAMtools, and any reads mapping to the Duke Encode blacklist regions were excluded using Bedtools (2.17.0)^104^. Finally, the *Drosophila* mapped reads were quality-checked for overlap by aligning with the human GRCh37 genome. The final normalized read counts for each ChIP-seq sample were calculated by dividing the total number of human mapped reads by the total number of *Drosophila* mapped reads. This provided a scaling factor for normalization of each sample. Ngs.plot.r was used to visualize the ChIP-seq signal across TSSs ±4 kb by generating heatmaps and average distribution/profile line plots^105^. The following parameters were used: -G hg19, -L 10000, -IN 1, -GO none, -D refseq. ChIP-seq data were compared to existing, publicly available RNA-seq of HEK293T cells in control conditions (GEO GSE158834)^106^.

### MS-based assays

#### KDM assays using MALDI–TOF MS

MALDI–TOF assays were conducted following a procedure with minor adaptations^2^. Experiments were conducted in HEPES buffer (50 mM) (at pH 7.5, adjusted using KOH). The peptide mixture ((+)-sodium-l-ascorbate (1.0 mM), (NH_4_)_2_Fe(II)(SO_4_)_2_ (100 µM), 2-oxoglutaric acid (200 µM), H3(1–21)K9me2 (20 µM), (TCEP (1,000 µM) in buffer) (5 µl) was added to the protein mixture (KDM3A (0.3 µM) in buffer) (5 µl) at 37 °C. At the appropriate time points, the samples were quenched with formic acid solution (formic acid (2% (vol/vol)) in H_2_O) (5.0 µl). For time-course experiments, a 50-µl final volume reaction mixture was incubated at 37 °C. At appropriate time points, an aliquot (5.0 µl) was removed and quenched with formic acid solution (5.0 µl), then the quenched sample (1.0 µl) was spotted on a MALDI–TOF target plate (Bruker MSP 96 target polished steel BC part no. 8280800) followed by the addition and mixing of MALDI matrix (CHCA (saturated, 10 mg ml^−1^) in TFA, acetonitrile and water (0.1:50:50)) (1.0 µl). The mixture was air-dried, then analysed using a Bruker Daltonics microflex instrument (flexControl, v. 3.4, build: 169.1) or Bruker rapifleX machines in positive-ion reflectron mode. The instrument was operated and data analysed using flexAnalysis software (v. 3.4, build: 79). Data were processed using Microsoft Excel and GraphPad Prism (v. 9.5.0), and the final graphic images were produced using Adobe Illustrator CS6.

#### Labelling experiments under different atmospheric conditions as determined using MALDI–TOF MS

Labelling experiments under controlled ^16^O_2_, ^18^O_2_ (^18^O_2_, 97 at% ^18^O, Merck) or N_2_ environments, with KDM3A and H3(1–21)K9ac, were performed using a Schlenk line set-up as described in ref. ^107^. Before the experiment, all solutions and solids were transferred into an anaerobic chamber (Belle Technology, O_2_ concentration <2 ppm) and equilibrated for 2 h. Stock solutions of sodium l-ascorbate (500 mM), 2OG (100 mM) and TCEP (100 mM) were prepared in MilliQ-grade H_2_O and Fe(II)SO_4_ (400 mM) in 20 mM HCl. Solutions were diluted to the final working concentrations of sodium l-ascorbate (10 mM), 2OG (2 mM), TCEP (10 mM) in HEPES buffer (50 mM in H_2_O, pH 7.5) and FeSO_4_ (1 mM in H_2_O). The final reaction mixture (200 ml) contained the following: KDM3A (2 mM), H3(1–21)K9ac (40 mM), sodium l-ascorbate (1 mM), 2OG (200 mM), TCEP (1 mM) and FeSO_4_ (100 mM). The anaerobic sample mixture was transferred into a J Young valve containing a pear-shaped tube sealed with a rubber septum. This gas-tight tube was removed from the anaerobic chamber and connected to the Schlenk line set-up connected to either ^16^O_2_, ^18^O_2_ (97 at% ^18^O, Merck) or N_2_. Residual O_2_ was removed from the Schlenk line system by repeated purging with argon, followed by application of vacuum. To create a mild vacuum in the sample-containing glass tube, the pressure in the Schlenk line set-up was adjusted to 700 mbar and the J Young valve was opened. The Schlenk line set-up including the sample was filled with either ^16^O_2_, ^18^O_2_ or N_2_ and the sample left to equilibrate for 5 min. The J Young valve of the ^16^O_2_-, ^18^O_2_- or N_2_-containing sample was closed and the sample removed from the Schlenk line set-up and incubated for 2 h at 37 °C. To quench the reaction, the sample was transferred into an anaerobic chamber and 20–50 ml of formic acid (10% (vol/vol)) was added though the rubber septum with a syringe, before MALDI–TOF MS analysis as described already.

#### KDM3A/B assay using intact histone H3.2K9ac substrate as analysed using LC–MS

Experiments were conducted in buffer consisting of HEPES (50 mM) (adjusted to pH 7.5 using KOH) in H_2_O. The peptide mixture ((+)-sodium-l-ascorbate (1.0 mM), (NH_4_)_2_Fe(II)(SO_4_)_2_ (100 µM), 2OG (200 µM), histone H3.2K9ac (20 µM), TCEP (1,000 µM) in buffer) (10 µl) was added to the protein mixture (KDM3A (0.3 µM) in buffer) (10 µl) at 37 °C (Eppendorf ThermoMixer C) and mixed. The reaction was quenched with aqueous formic acid (5% (vol/vol) in H_2_O). The samples were diluted by adding MilliQ-grade H_2_O to a final peptide concentration of ~5 μM. Peptide analysis was performed by LC–MS using an Agilent 1290 Infinity II LC system connected to an Agilent 6550 accurate mass iFunnel quadrupole time of flight (QTOF) mass spectrometer. Samples (4 ml) were injected and loaded onto a ZORBAX RRHD Eclipse Plus C18 column (Agilent). Solvent A consisted of LC–MS-grade water containing 0.1% (vol/vol) formic acid, and solvent B consisted of acetonitrile containing 0.1% (vol/vol) formic acid. Peptides were separated using a stepwise gradient (0 min—1% (vol/vol) solvent B, 2.0 min—1% (vol/vol) solvent B, 5.0 min—30% (vol/vol) solvent B, 10 min—95% (vol/vol) solvent B, 11.0 min—95% (vol/vol) solvent B, 12.0 min—5% (vol/vol) solvent B). This was followed by a 3-min post run with 99% solvent A to re-equilibrate the column; all flow rates were 0.2 ml min^−1^. The mass spectrometer was operated in positive-ion mode with a drying gas temperature of 280 °C, drying gas flow rate of 13 l min^−1^, nebulizer pressure of 40 p.s.i.g., sheath gas temperature of 350 °C, sheath gas flow rate of 12 l min^−1^, capillary voltage of 4,000 V and nozzle voltage of 1,000 V. All acquired data were analysed using Agilent MassHunter Qualitative Analysis (v. B.07.00) software. Data were processed using Microsoft Excel and GraphPad Prism (v. 9.5.0), and the final graphic image was produced using Adobe Illustrator CS6.

#### HDAC8 and SIRT1 MALDI–TOF MS assays and analysis

The HDAC8 protein mixture (2 µM HDAC8(1–377, HDAC8_FL_) in buffer (HEPES pH 7.8, 137 mM NaCl, 3.7 mM KCl)) and the substrate mixture (10 µM H3(1–20)K9ac or H3(1–20)K9acOH peptide, 2.5 mM sodium l-ascorbate, 10 µM (NH4)_2_Fe(SO_4_)_2_ in buffer) were mixed and incubated at 37 °C for 45 min. The SIRT1 assay enzyme mixture (0.05 or 2 µM SIRT1 in buffer (50 mM HEPES pH 7.6, 50 mM NaCl)) and substrate mixture (10 µM H3(1–20)K9ac or H3(1–20)K9acOH peptide, 50 µM NAD^+^ (Sigma, N1511) in buffer) were mixed and incubated at 37 °C for 45 min.

The enzyme mixture and substrate mixture (peptide and cofactor) were prepared separately at double the final concentrations used for reaction. The substrate mixture was added to an equal volume of the enzyme mixture to initiate reaction. For each time point, 10 µl of the reaction mixture was withdrawn and quenched with 10 µl of 2% (vol/vol) HCOOH in water. Time-course assays were carried out with *n* = 3 independent assay repeats, with each assay replicate having *n* = 3 technical replicates. For the negative control, 5 µl of the enzyme mixture was pre-quenched with 10 µl of 2% (vol/vol) formic acid (HCOOH) before addition of the substrate mixture (5 µl). Each time point was spotted onto a 96-spot MALDI target plate and mixed in a 1:1 ratio (vol/vol) with a saturated solution of CHCA dissolved in 50% (vol/vol) acetonitrile and 0.01% (vol/vol) 1:1 aqueous TFA. The dried spots were analysed using a Bruker microflex LRF machine (Bruker Daltonics). Data were analysed using flexAnalysis v3.4 (Bruker Daltonics).

#### Histone analysis using MS

Derivatization and digestion of histones were performed as described in ref. ^72^. In brief, histones were dissolved in 20 μl of 50 mM NH_4_HCO_3_ (pH 8.0). The derivatization reagent was prepared by mixing the sample with 5 µl of acetonitrile, followed by the addition of 5 µl of propionic anhydride and 14 µl of ammonium hydroxide (to give pH 8.0) for 15 min at 37 °C. This reaction was performed twice. Histones were digested with trypsin for 2 h at room temperature (enzyme:sample ratio of 1:20) in 50 mM NH_4_HCO_3_. Synthetic peptides were added after the digestion of intact histones. Samples were desalted before nano-LC–MS/MS analysis using in-house packed stage tips. Stage tips were prepared using HLB resin (Waters). The stage tips were equilibrated with 50 µl of water + 0.1% (vol/vol) TFA, and the sample was loaded after adding 1% (vol/vol) TFA to the solution. The tips were washed twice with 30 µl of water + 0.1% (vol/vol) TFA, and elution was performed by using 30 µl of 60% (vol/vol) acetonitrile + 0.1% (vol/vol) TFA. The samples were then dried before MS analysis.

Histone peptides were resuspended in 10 µl of water + 0.1% (vol/vol) TFA, then analysed as reported in ref. ^73^. In brief, a nano-LC machine was linked to a 75-µm ID × 25-cm Reprosil-Pur C_18_-AQ (3 µm; Dr Maisch) nano-column packed in-house using a Dionex RSLC Ultimate 3000 machine (Thermo Scientific). The HPLC gradient used was as follows: 2% (vol/vol) to 28% (vol/vol) solvent B (A = 0.1% (vol/vol) formic acid; B = 80% (vol/vol) acetonitrile, 0.1% formic acid) over 45 min, from 28% (vol/vol) to 80% (vol/vol) solvent B in 5 min, 80% (vol/vol) B for 10 min at a flow rate of 300 nl min^−1^. The nano-LC machine was coupled to an Orbitrap Fusion Lumos mass spectrometer (Thermo Scientific). The data-independent acquisition (DIA) method was used as described in ref. ^74^, consisting of a full-scan MS spectrum (*m*/*z* 300−1,100) at 120,000 resolution (*m*/Δ*m* for the ion at 200 *m*/*z*), followed by 16 MS/MS analysis with windows of 50 *m*/*z* using HCD fragmentation and 15,000 resolution. For the analysis of PTMs, peptide abundance was normalized to total peptides. DIA data obtained from the nano-LC–MS/MS runs were searched using EpiProfile 2.0^108^ or manually extracted using Skyline software.

#### KAT2B MALDI–TOF MS assays and analysis

The KAT2B enzyme mixture (0.4 µM His-KAT2B) and substrate mixture (40 µM H3(1–21), H3(1–21)K9ac or H3(1–21)K9acOH peptide, 100 µM acetyl coenzyme A) were prepared in 50 mM HEPES, 50 mM NaCl, pH 7.5. The substrate mixture (60 µl) was added to the KAT2B mixture (60 µl) to initiate reaction at room temperature. For each time-point analysis, 10 µl of the reaction mixture was withdrawn and quenched with 10 µl of 2% (vol/vol) HCOOH in water. Time-course assays were carried out with *n* = 2 independent assay repeats, each with *n* = 2 technical replicates. For the negative control, 5 µl of the enzyme mixture was pre-quenched with 10 µl of 2% (vol/vol) formic acid (HCOOH) before addition of the substrate mixture (5 µl). Each time point was spotted onto a 96-spot MALDI target plate and mixed in a 1:1 ratio (vol/vol) with a saturated solution of CHCA dissolved in 50% (vol/vol) acetonitrile and 0.01% (vol/vol) 1:1 aqueous TFA. The dried spots were analysed using a Bruker Microflex LRF machine (Bruker Daltonics). Data were analysed using flexAnalysis v3.4 software (Bruker Daltonics).

#### Protein production and isolation of recombinant proteins

Recombinant histone lysine demethylases KDM4A (M1-L359)^1^, KDM4D (M1-L358)^2^, KDM4E (M1-Q337)^3^, KDM5A (M1-L801)^109^ and KDM7B (M37-N483)^5^ were produced and purified following procedures. Recombinant full-length KDM3A (M1-S1321, Active Motif, 31456) and KDM3B (M1-S1761, Active Motif, 31429) were from commercial sources. FDH (M1-399) from *Pseudomonas putida* was produced as described in ref. ^110^.

#### Production and purification of recombinant KDM3A

DNA sequences encoding for human KDM3A(T515-S1317) were cloned into the pFB-CT10HF-LIC vector via ligation-independent cloning (LIC)^111^. Exponentially growing Sf9 insect cells (2 × 10^6^ cells ml^−1^) were infected with high-titre baculovirus stock and incubated for 70 h at 27 °C and 90 r.p.m. Cells were collected by centrifugation (800*g*, 15 min, 4 °C), resuspended in PBS, centrifuged (800*g*, 15 min, 4 °C), and stored at −80 °C. The thawed cells were resuspended in lysis buffer (50 mM HEPES pH 7.4, 200 mM NaCl, 20 mM imidazole, 5% (vol/vol) glycerol and 0.5 mM TCEP) supplemented with 1:2,000 Complete, EDTA-free protease inhibitor cocktail (Roche Diagnostics). The cells were lysed by sonication (2 min, amplitude 35%, on ice) and the insoluble lysates were removed by centrifugation (36,000*g*, 60 min, 4 °C). The soluble supernatant was combined with 7.5 ml of Ni(II)-charged immobilized metal affinity chromatography (IMAC) Sepharose and loaded onto a gravity flow column. The column was washed with lysis buffer (the protein was eluted with lysis buffer containing 300 mM imidazole). The eluted protein was pooled, concentrated to ~5 ml, then purified by gel filtration (GF) using an AKTA Xpress system with an S200 16/600 column and GF buffer (20 mM HEPES pH 7.4, 500 mM NaCl, 5% (vol/vol) glycerol, 0.5 mM TCEP). Fractions containing KDM3A^(515–1317)^ were pooled and concentrated, and the purity and identity was confirmed by SDS–polyacrylamide gel electrophoresis (PAGE) and by MS.

#### Production of recombinant KDM3B and JMJD1C

DNA sequences encoding for KDM3B(Q879-S1716) and JMJD1C(L1760-N2540) were cloned into the pFB-LIC-Bse vector using LIC. Sf9 insect cells (2 × 10^6^ cells ml^−1^) were infected with baculovirus stock, incubated for 48 h (27 °C, 100 r.p.m. in non-baffled flasks), collected (900*g*, 10 min), then stored (−80 °C). The cell pellet was thawed, suspended in buffer (10 mM imidazole, 0.5 mM TCEP, 50 mM HEPES (pH 7.5), 300 mM NaCl and 5% (vol/vol) glycerol in water) supplemented with protease inhibitor cocktail set VII (Calbiochem) and sonicated (35% amplitude, 5 s on, 10 s off for a total of 3 × 3 min). Insoluble material was separated by centrifugation (55,000*g*). Proteins were isolated by nickel-affinity chromatography (4 °C) following a stepwise imidazole gradient with wash buffer (10 or 20 mM imidazole, 0.5 mM TCEP, 50 mM HEPES (pH 7.5), 300 mM NaCl and 5% (vol/vol) glycerol in water) followed by elution with elution buffer (250 mM imidazole, 0.5 mM TCEP, 50 mM HEPES (pH 7.5), 300 mM NaCl and 5% (vol/vol) glycerol in water). Fractions containing the desired protein were combined and concentrated, then incubated with tobacco etch virus (TEV) protease (4 °C, 16 h) and purified using size-exclusion chromatography (SEC; Superdex 200, GE/Amersham Biosciences) with GF buffer (0.5 mM TCEP, 20 mM HEPES (pH 7.6), 300 mM KCl and 5% (vol/vol) glycerol in water). Selected fractions based on SDS–PAGE and LC–MS data were combined, concentrated (50-kDa molecular weight cutoff (MWCO)) and stored (­80 °C).

#### Production of recombinant KDM5D

A pFB-LIC-Bse vector encoding for the KDM5D (M1-D775) gene was transformed into baculovirus and Sf9 cells (2 × 10^6^ cells l^−1^). Cells infected with the virus stock were grown with shaking (27 °C, 90 r.p.m., 60 h), collected by centrifugation, washed with PBS and then subjected to another round of centrifugation, then stored (−80 °C). After thawing, the cells were suspended (100 ml) in lysis buffer (TCEP (500 µM), imidazole (20 mM), NaCl (200 mM), HEPES (50 mM, pH 7.4) in water) followed by sonication (35% power amplitude, 2 min), and the suspension was centrifuged. The supernatant was loaded onto a Ni(II)-charged IMAC Sepharose (GE Healthcare) column, washed (100 ml) with wash buffer A (TCEP (500 µM), glycerol (5% (vol/vol)), imidazole (20 mM), NaCl (500 mM), HEPES (50 mM, pH 7.4) in water), wash buffer B (50 ml) (TCEP (500 µM), imidazole (40 mM), NaCl (1,000 mM), HEPES (50 mM, pH 7.4) in water), wash buffer C (50 ml) (TCEP (500 µM), imidazole (60 mM), NaCl (500 mM), HEPES (50 mM, pH 7.4) in water) and eluted (25 ml) with elution buffer (TCEP (500 µM), imidazole (300 mM), NaCl (500 mM), HEPES (50 mM, pH 7.4) in water). Fractions containing the desired protein were combined and concentrated by centrifugation, then further purified using gel GF (Superdex 200 column) using GF buffer (TCEP (500 µM), glycerol (5% (vol/vol)), NaCl (500 mM), HEPES (20 mM, pH 7.4) in water). The purity of the fractions was determined by SDS–PAGE analysis, and selected fractions were pooled, concentrated and stored (−80 °C).

#### Production of recombinant KAT2B (PCAF)

The plasmid pNIC-Bio3 (SGC Oxford) encoding for human KAT2B (V493-E658) was transformed into Rosetta 2(DE3)pLysS Singles competent cells (Novagen). Auto Induction Media Terrific Broth (AIM-TB, Formedium) (2 × 1 l) was inoculated with a TB starter culture (1 ml l^−1^). The cells were grown for 4 h at 37 °C and 200 r.p.m., then for 48 h at 18 °C and 200 r.p.m., then collected by centrifugation (18,600*g*, 20 min, 4 °C) and stored at −80 °C. The cells were thawed and resuspended in 50 ml of lysis buffer (10 mM HEPES pH 7.6, 500 mM NaCl, 5% (vol/vol) glycerol, 0.5 mM TCEP, 20 mM imidazole) supplemented with DNase I (Roche, 10104159001). The cells were lysed using a Cell Disruptor (Constant Systems), then the insoluble lysates were removed by centrifugation (39,800*g*, 20 min, 4 °C). The soluble fraction was then passed through 0.45-µm and 0.2-µm filters, then loaded onto a 2-ml Ni(II)-charged IMAC Sepharose (GE Healthcare) column. The column was washed with lysis buffer, and the proteins were eluted with an imidazole gradient. Eluted fractions were analysed by SDS–PAGE, and His-KAT2B_HAT_-containing fractions were pooled. TEV protease (a gift from I. Pettinati) was added (2 µl of 90 mg ml^−1^) to the mixture for His-tag cleavage, and the solution was buffer-exchanged using dialysis tubing (Snakeskin, ThermoFisher) overnight into 20 mM HEPES pH 7.6, 500 mM NaCl, 5% (vol/vol) glycerol. The dialysed protein solution was loaded onto a 2-ml Ni(II)-charged IMAC Sepharose gravity column, and incubated for 30 min. The flow-through was collected and the resin washed with lysis buffer. Eluted fractions were analysed by SDS–PAGE, and KAT2B_HAT_-containing fractions were pooled, buffer-exchanged into lysis buffer, and concentrated using Amico ultracentrifugal filters (Merck).

#### Production of recombinant SIRT1

The SIRT1.1 (Addgene 13735) plasmid encoding for N-terminal 6× His-tagged human SIRT1(E82-S747, SIRT_CD_) was transformed into Rosetta 2(DE3)pLysS Singles competent cells (Novagen). 2YT medium (2 × 1 l) was inoculated with a 2YT starter culture (1 ml l^−1^). The cells were grown at 37 °C and 200 r.p.m. At an optical density at 600 nm (OD_600_) of ~0.6, the cultures were cooled to 25 °C; after 60 min, expression was induced with isopropyl-β-d-1-thiogalactopyranoside (IPTG; 0.5 mM). The cells were incubated at 25 °C and 200 r.p.m. for 12 h, then collected by centrifugation (18,600*g*, 20 min, 4 °C) and stored at −80 °C. The cells were thawed and resuspended in 15 ml of lysis buffer (50 mM Tris pH 8.0, 250 mM NaCl, 20 mM imidazole, 0.5 mM TCEP) supplemented with DNase I (Roche) and protease inhibitor cocktail (Roche, 11836170001). They were lysed by sonication (using a Vibra-Cell VXC500 instrument) on ice with six cycles (60% amplitude) of 30 s on (0.5 s on/0.5 s off)/30 s off, and insoluble lysates were removed by centrifugation (39,800*g*, 20 min, 4 °C). The soluble fraction was then passed through a 0.2-µm filter and loaded onto a 2-ml Ni(II)-charged IMAC Sepharose (GE Healthcare) gravity column. The column was washed with lysis buffer; protein was then eluted with a 20–500 mM imidazole gradient. Eluted fractions were analysed by SDS–PAGE, and SIRT1_CD_-containing fractions were pooled. SIRT1 was further purified by SEC using a HiLoad 16/600 Superdex 200 pg column (GE Healthcare) at 1 ml min^−1^ with 50 mM Tris pH 8.0, 250 mM NaCl and 5% glycerol. SIRT1_CD_-containing fractions were analysed by SDS–PAGE, pooled, and concentrated with Amicon ultracentrifugal filters.

#### Production of recombinant HDAC8_FL_ (HDAC8, KDAC8)

DNA encoding for the CD of human HDAC8 was cloned into the pNIC28-Bsa4 vector (Addgene 26103). Plasmid HDAC8A-c100 (SGC Oxford) encoding for N-terminal 6× His-tagged HDAC8 (M1V377) was transformed into *Escherichia coli* BL21(DE3)-R3-pRARE2 competent cells. TB medium (4 × 1 l), supplemented with 0.4% (vol/vol) glycerol and 200 µM ZnCl_2_ was inoculated with starter culture (15 ml l^−1^). The cells were grown at 37 °C and 180 r.p.m. until an OD_600_ of ~ 2.5 was reached, then incubated at 18 °C; after 30 min, expression was induced with IPTG (0.1 mM). Cultures were maintained overnight at 18 °C and 180 r.p.m., then collected by centrifugation. The cells were thawed and resuspended in 160 ml of lysis buffer (50 mM HEPES pH 7.5, 250 mM NaCl, 10 mM imidazole, 5% (vol/vol) glycerol, 0.5 mM TCEP) supplemented with 5 U ml^−1^ benzonase nuclease (Novagen), 0.1 mg ml^−1^ lysozyme and protease inhibitors. The cells were lysed by sonication, and insoluble lysates were removed by centrifugation (40,000*g*, 45 min, 4 °C). The soluble fraction was incubated with 5 ml of Ni^2+^-charged IMAC Sepharose (Cytiva) for 30 min at 4 °C, then centrifuged (500*g* for 5 min). The resin was resuspended in 100 ml of lysis buffer and added to a gravity column, then further washed with 50 ml of wash buffer (50 mM HEPES pH 7.5, 250 mM NaCl, 20 mM imidazole, 5% (vol/vol) glycerol). HDAC^(1–377)^ was eluted with elution buffer (50 mM HEPES pH 7.5, 250 mM NaCl, 250 mM imidazole, 5% (vol/vol) glycerol, 0.5 mM TCEP in H_2_O). Eluted fractions were analysed by SDS–PAGE, and HDAC8_FL_-containing fractions were pooled. TEV protease was added to enable His-tag cleavage. The solution was buffer-exchanged using 3-K MWCO dialysis tubing overnight at 4 °C into 25 mM HEPES pH 7.5, 127 mM NaCl, 3 mM KCl, 5% (vol/vol) glycerol and 0.5 mM TCEP. The dialysed protein solution was loaded onto a 0.5-ml Ni^2+^-charged IMAC Sepharose gravity column (GE Healthcare) and the flow-through was collected. The eluted protein was analysed by SDS–PAGE, and HDAC_FL_-containing fractions were pooled, then concentrated using 10-K MWCO Amicon ultracentrifugal filters (Millipore).

#### Histones

Histones from mammalian cell lines were purified, precipitated and resuspended in H_2_O (Sigma, W4502) using a histone purification kit (Active Motif, 40026) following the manufacturer’s recommended conditions. Histones from calf thymus (Sigma, H9250), recombinant histone H3/H4 tetramer (Active Motif, 81169) and recombinant histone H3.2K9ac (Active Motif, 31253) were purchased.

#### Peptides used in this research

The peptides H3(4–13) K9acOH: KQTARKSTGGC, H3(5–13) K9acOH: CQTARKSTGG, H3(1–20): ARTKQTARKSTGGKAPRKQL, H3(1–20) K9ac: ARTKQTARKSTGGKAPRKQL, H3(1–20) K9acOH: ARTKQTARKSTGGKAPRKQL, H3(1–20) K14acOH: ARTKQTARKSTGGKAPRKQL, H3(15–34) K27acOH: Ac-APRKQLATKAARKSAPATGG were synthesized with a C-terminal acid, purified by HPLC (>80%) and quality-control-tested by DC Biosciences.

Peptides H3(1–21)K9me1, H3(1–21)K9me2, H3(1–21)K9me3, H3(1–21)K9ac, H3(1–21)K4me3K9me2, H3(1–21)K4me3 and H3(1–21)K4me_3_K9ac were purchased from GL-Biochem as C-terminal amides. Peptides H3(1–15K9ac), H3(1–44)K9ac, H3(1–21)K9ac-^2^H_3_ and H3(1–21)K9acOH were synthesized with C-terminal amides and purified in-house (Supplementary Information). The concentrations of peptide stock solutions for histone demethylation assays were determined by standard ^1^H-NMR, correlating an assigned peptide resonance signal with that of an internal standard (sodium trimethylsilane propionate).

#### Peptide synthesis and purification

In-house-produced peptides were synthesized by solid-phase peptide synthesis using a Gyros Protein Technologies (PurePep Chorus) synthesizer using the Rink amide resin. Coupling of Fmoc monomers was achieved with diisopropylcarbodiimide and Oxyma Pure (Novabiochem) in DMF. Deprotection of the Fmoc group was conducted using piperidine in DMF (20%). The peptide was released from acid-labile protecting groups and the solid support using a solution of TFA:H_2_O:triisopropylsilane (95:2.5:2.5 vol/vol/vol). The solution was filtered, then the mixture was added to cooled (−24 °C) diethyl ether to give a white precipitate. The mixture was centrifuged, the supernatant decanted, and the solid was washed twice more with diethyl ether. The solid was air-dried, dissolved in water, and purified using an Agilent HPLC machine equipped with a C18 column using a linear gradient of (TFA (0.1% (vol/vol)) in acetonitrile) in (TFA (0.1% (vol/vol)) in H_2_O). The fractions were pooled based on UV (210 nm) analysis, analysed by MALDI–TOF MS in positive-ion and reflectron mode, and lyophilized to yield a white solid. Peptide mass confirmation and corresponding LC–MS plots are provided in Supplementary Table 6 and Supplementary Fig. 19, respectively.

### Reporting Summary

Further information on research design is available in the Nature Portfolio Reporting Summary linked to this Article.

## Online content

Any methods, additional references, Nature Portfolio reporting summaries, source data, extended data, supplementary information, acknowledgements, peer review information; details of author contributions and competing interests; and statements of data and code availability are available at 10.1038/s41557-026-02112-x.

## Supplementary information

Supplementary Information Supplementary Figs. 1–11, Tables 1–5, Synthesis of amino-acid monomers, NMR spectra, peptide HRMS and purity, HPLC traces of peptides, uncropped western blot images from Supplementary Fig. 10, references.

Reporting Summary

## Source data

Source Data Fig. 1 Raw MS data for Fig. 1h.

Source Data Fig. 2 Uncropped western gel blots for Fig. 2a,b and immunofluorescence data analysis for Fig. 2f.

Source Data Fig. 3 Uncropped western gel blots for Fig. 3a,b,d.

Source Data Extended Data Fig. 4 Raw data for bar charts in Extended Data Fig. 4m,o.

Source Data Extended Data Fig. 6 Raw data for graphs in Extended Data Fig. 6m,n, and bar chart in Extended Data Fig. 6o.

Source Data Extended Data Fig. 7 Uncropped western gel blots for Extended Data Fig. 7c–g.

Source Data Extended Data Fig. 8 Immunofluorescence data analysis of Extended Data Fig. 8d.

Source Data Extended Data Fig. 9 Raw data for graphs Extended Data Fig. 9c,f,g,h.

Source Data Extended Data Fig. 10 Raw data for graph.

## Data Availability

The MS proteomic data for histone are available through the Proteomics IDEntification database (PRIDE, accession no. PXD057969). ChIP-sequencing data have been deposited in the NCBI Gene Expression Omnibus (GEO) database (accession no. GSE282321). Immunofluorescence data have been deposited in the Newcastle University data repository (data.ncl.ac.uk) at 10.25405/data.ncl.30853637. Source data are provided with this paper.
