# Supplementary material for: KDM3A catalyses the oxidation of acetyl-lysine to hydroxyacetyl-lysine on histone H3K9
Source: Nat Chem. 2026 Apr 15;18(5):823–34. doi: 10.1038/s41557-026-02112-x (PMC13149330; doi:10.1038/s41557-026-02112-x)
Supplement: Supplementary file 1 — Supplementary Figs. 1–11, Tables 1–5, Synthesis of amino-acid monomers, NMR spectra, peptide HRMS and purity, HPLC traces of peptides, uncropped western blot images from Supplementary Fig. 10, references. [file 41557_2026_2112_MOESM1_ESM.pdf]

nature chemistry

Supplementary information https://doi.org/10.1038/s41557-026-02112-x

KDM3A catalyses the oxidation of acetyl-lysine to hydroxyacetyl-lysine on histone H3K9

In the format provided by the authors and unedited

Table of contents

1 Supplementary Figures 2

2 Supplementary Tables 13

3 Synthesis of amino acid monomers 17

4 NMR spectra 20

5 Overview of peptides used in this work 24

6 Analytical HPLC UV traces of peptides 25

7 Uncropped western blot images 35

8 References 37

1 Supplementary Figures

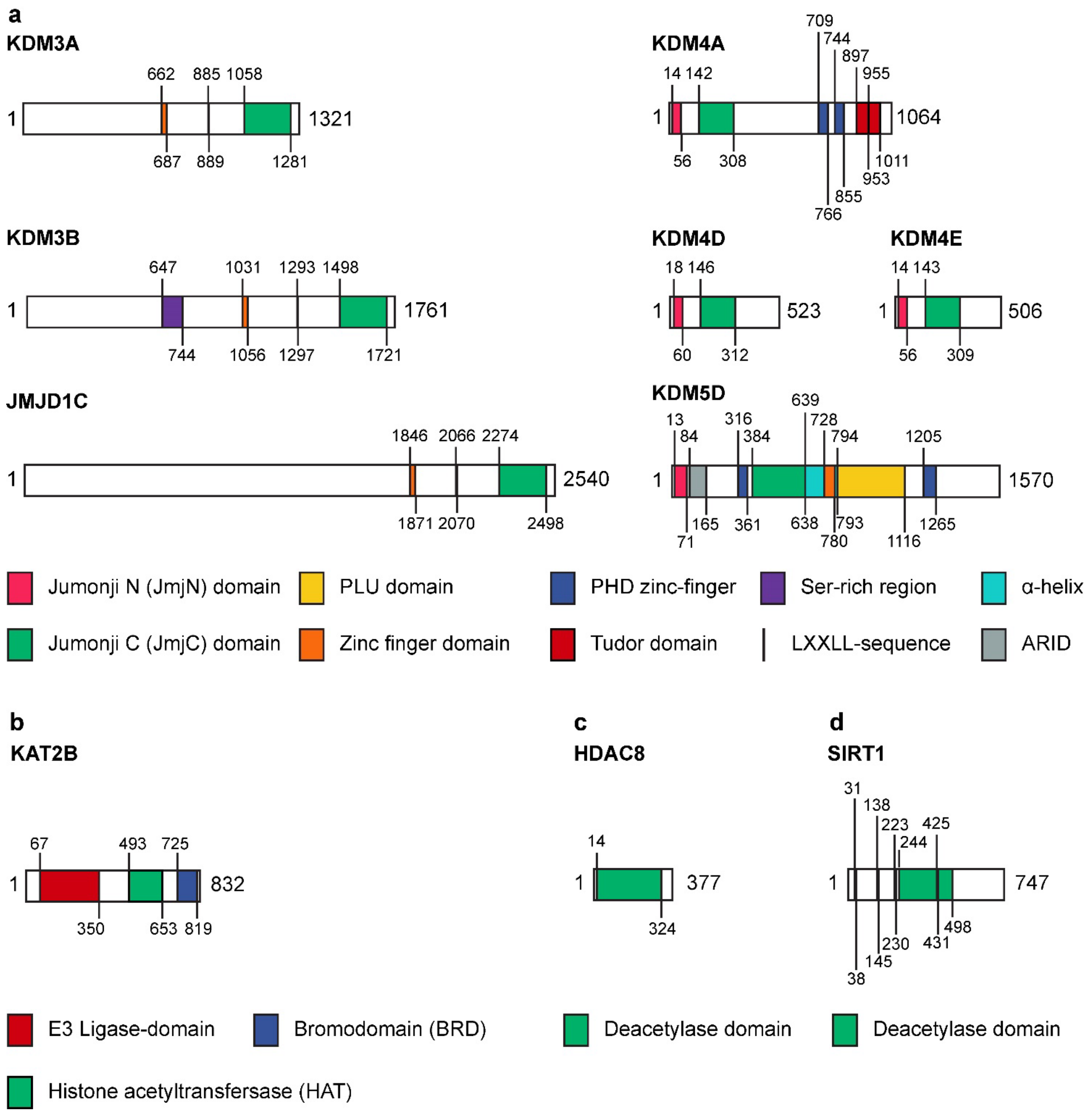

Supplementary Fig. 1 | Structural domains of human enzymes used in this work. a. Jumonji C domain-containing histone lysine demethylases (JmjC-KDMs), **b.** histone lysine acetyltransferase (HAT) **c, d.** histone lysine deacetylases (HDAC). Catalytic domains associated with the installation or removal of histone lysine PTMs are coloured green.

a

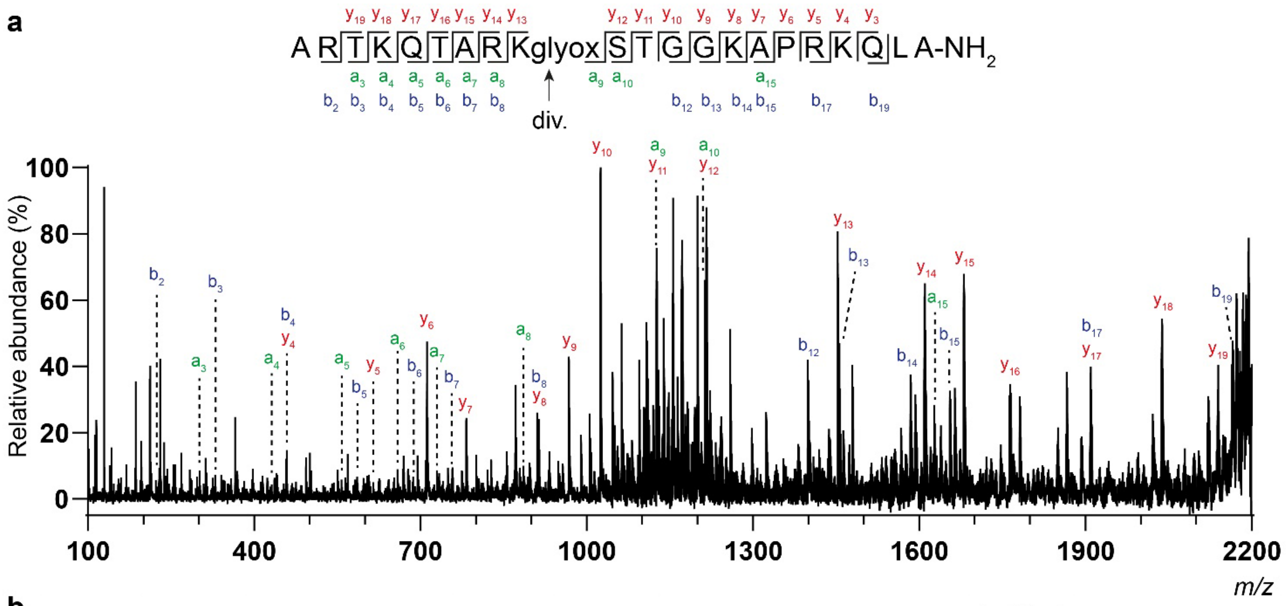

b

Ion		Ala 1	Arg 2	Thr 3	Lys 4	Gln 5	Thr 6	Ala 7	Arg 8	Lys(glyox) (div.) 9	Ser 10	Thr 11
a	Calculated	44.050	200.151	301.198	429.293	557.352	658.400	729.437	885.538	1125.933	1212.965	1314.012
	Found			301.328	429.423	557.576	658.596	729.640	885.778	1125.912	1212.958	
b	Calculated	72.044	228.146	329.193	457.288	585.347	686.394	757.432	913.533	1153.928	1240.960	1342.007
	Found		228.266	329.340	457.454	585.517	686.560	757.608	913.757			
y	Calculated	89.075	202.159	330.218	458.313	614.414	711.466	782.504	910.599	967.620	1024.641	1125.689
	Found				458.468	614.526	711.651	782.693	910.805	967.830	1024.869	1125.912
		Ala	Leu	Gln	Lys	Arg	Pro	Ala	Lys	Gly	Gly	Thr

Ion		Gly 12	Gly 13	Lys 14	Ala 15	Pro 16	Arg 17	Lys 18	Gln 19	Leu 20	Ala 21
a	Calculated	1371.034	1428.055	1556.150	1627.187	1724.240	1880.341	2008.436	2136.495	2249.579	2319.636
	Found				1627.265						
b	Calculated	1399.029	1456.050	1584.145	1655.182	1752.235	1908.336	2036.431	2164.490	2277.574	2347.631
	Found	1399.064	1456.095	1584.201	1655.250		1908.439		2164.672		
y	Calculated	1212.721	1453.116	1609.217	1680.254	1781.302	1909.361	2037.456	2138.503	2294.604	2365.641
	Found	1212.958	1453.134	1609.256	1680.312	1781.387	1909.436	2037.576	2138.642		
		Ser	Lys(glyox) (div.)	Arg	Ala	Thr	Gln	Lys	Thr	Arg	Ala

Supplementary Fig. 2| Identification of acetylhydrazine derivatized *N*ε-glyoxylyl-lysine 9 on histone H3 peptide from an KDM3A catalysed reaction using tandem MS (MS/MS). Recombinant KDM3A$_{CD}$ was incubated with H3(1-21)K9acOH for 60 min, acetyl-hydrazine was then added and the mixture was incubated for 60 min. MS/MS spectra were collected using MALDI-TOF/TOF MS. **a**, MS/MS spectrum of H3(1-21)K9glyox(div.) and **b**, table of calculated and observed MS/MS fragments of a-, b-, and y-ions. Calculated ion fragments highlighted in red are those that match the experimentally observed ion fragments (within ±1 Da tolerance). Conditions: KDM3A$_{CD}$ (2.0 µM), H3(1-21)K9acOH (10 µM), L-ascorbate (500 µM), Fe(II) (50 µM), 2OG (100 µM), and TCEP (500 µM) were reacted for (60 min, 37 °C), and acetyl-hydrazine (60 min, 37 °C). a-, b- and y-ions are in green, blue and red, respectively.

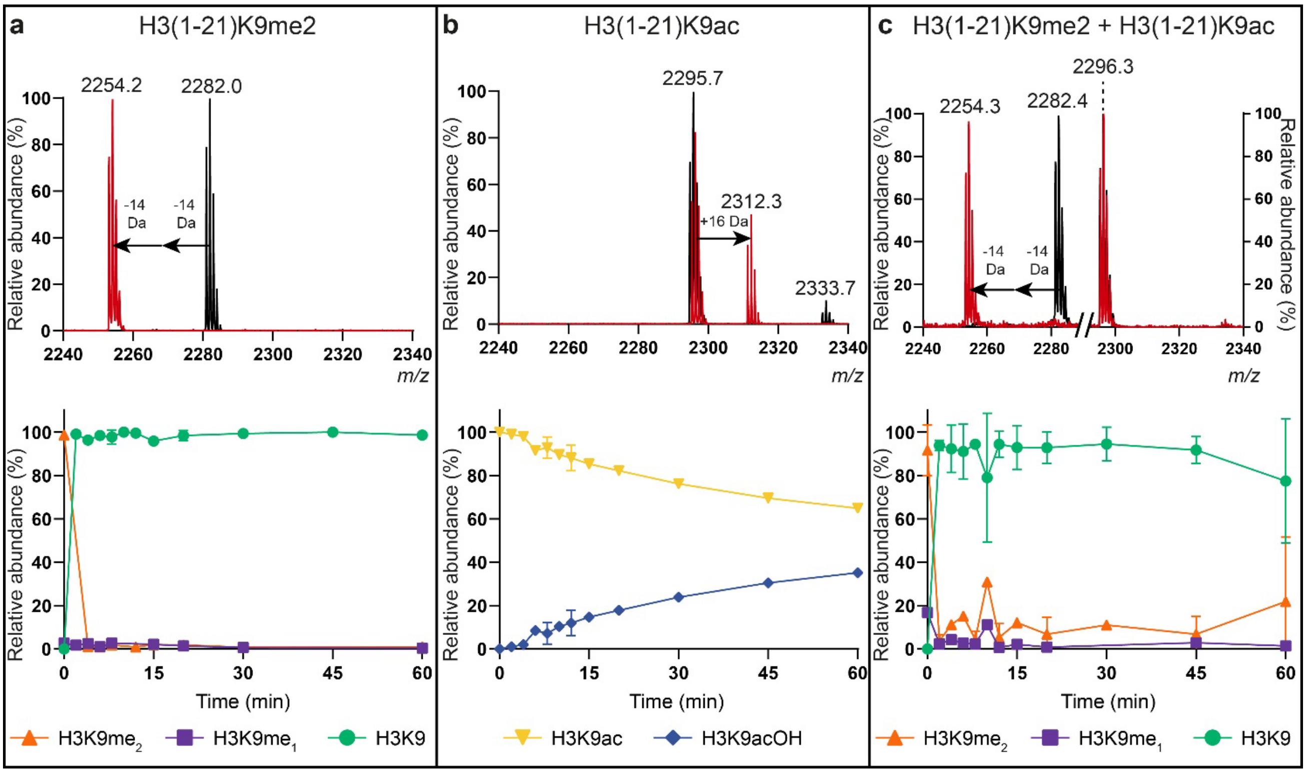

Supplementary Fig. 3 | KDM3A competition assays with H3(1-21)K9me2 and H3(1-21)K9ac. In the presence of KDM3A_{CD} (0.5 µM), (**a**) H3(1-21)K9me2 is demethylated and (**b**) H3(1-21)K9ac is hydroxylated. **c**, With both substrates in the same assay mixture, efficient demethylation of H3(1-21)K9me2 is observed; hydroxylation of H3(1-21)K9ac is not observed. Conditions: KDM3A_{CD} (0.5 µM), peptide (10 µM), L-ascorbate (500 µM), Fe(II) (50 µM), 2OG (100 µM), and TCEP (500 µM) were incubated at 37 °C. Overlaid MALDI-TOF spectra for 0 min (black) and 60 min (red) are shown, together with the corresponding time courses for each condition, plotted as mean ± SD (n = 3 technical replicates). Assays were repeated twice, with a representative dataset shown in **a**–**c**.

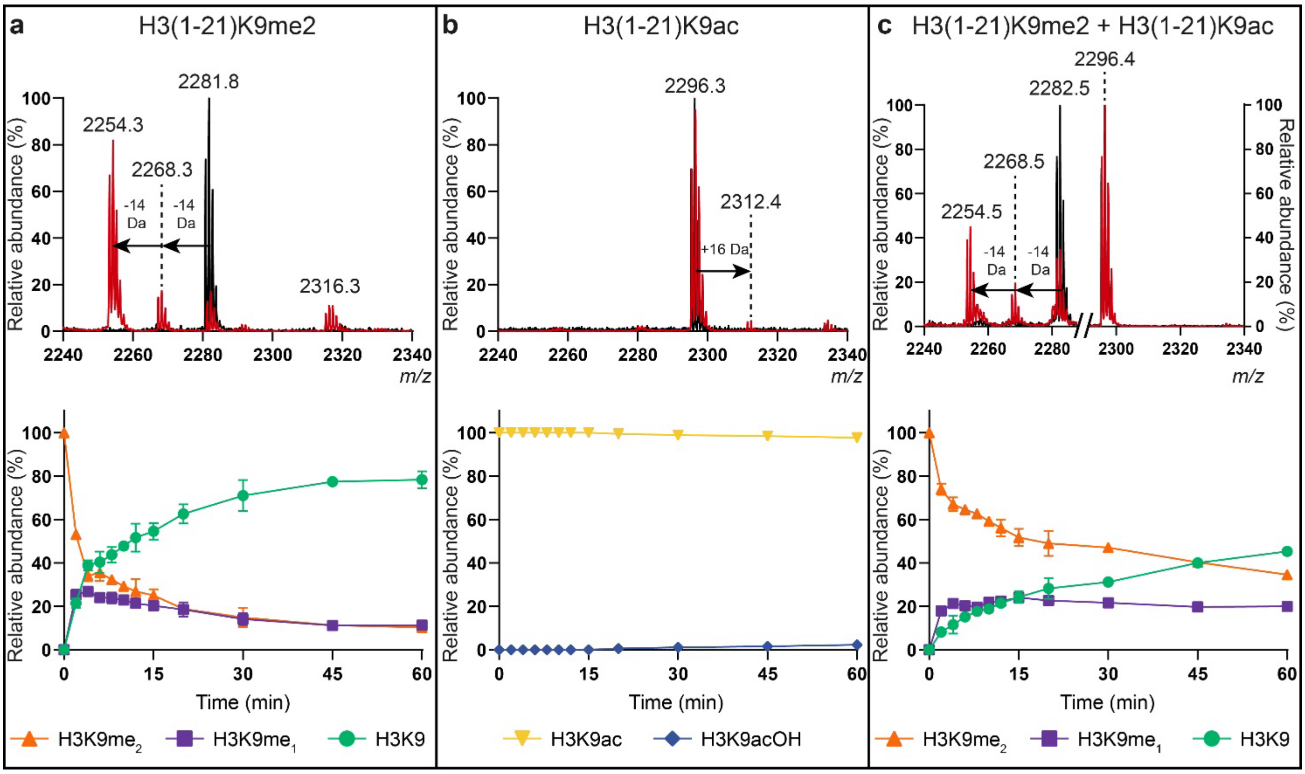

Supplementary Fig. 4 | KDM3A assays for inhibition of H3(1-21)K9me2 demethylation by H3(1-21)K9ac. a, With a reduced concentration of KDM3ACD (reduced from 0.5 µM, as in **Supplementary Fig. 8**, to 0.1 µM), a decreased rate of demethylation is observed for H3(1-21)K9me2 (10 µM), as expected. **b**, A low level of hydroxylation of H3(1-21)K9ac (10 µM) is observed after 60 min incubation under the same conditions. **c**, A decrease in the demethylation rate of H3(1-21)K9me2 (10 µM) is observed when equimolar amounts of H3(1-21)K9ac (10 µM) is added to the reaction mixture. No hydroxylation of H3(1-21)K9ac is detected. Conditions: KDM3ACD (0.1 µM), peptide (10 µM), L-ascorbate (500 µM), Fe(II) (50 µM), 2OG (100 µM), and TCEP (500 µM) were reacted at 37 °C. Overlaid MALDI-TOF spectra for 0 min (black) and 60 min (red) are shown, together with the corresponding time courses for each condition, plotted as mean ± SD (n = 3 technical replicates). Assays were repeated twice, with a representative dataset shown in **a**-**c**.

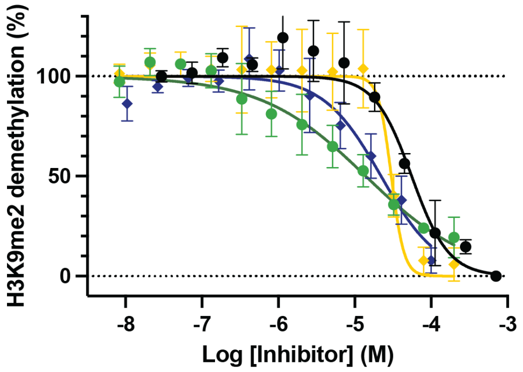

	pIC_{50} (M)	IC_{50} (μM)
NOG	4.49 ± 0.26	32.7
H3(1-21)K9	5.29 ± 0.46	5.1
H3(1-21)K9ac	4.63 ± 0.09	23.5
H3(1-21)K9acOH	4.46 ± 0.15	34.4

Supplementary Fig. 5 | Inhibition of KDM3A catalysed demethylation of H3(1-21)K9me2 by acetylated and non-acetylated H3K9 peptides, and by *N*-oxalylglycine (NOG) using the formaldehyde dehydrogenase (FDH) assay. Standard conditions: $KDM3A_{CD}$ (0.15 µM), formaldehyde dehydrogenase (0.5 µM), $H3(1\text{-}21)K9me_2$ (10 µM), L-ascorbate (500 µM), Fe(II) (50 µM), 2OG (100 µM), and ß-nicotinamide adenosine dinucleotide (NAD^+) (500 µM) were incubated at 37 °C. Mean ± SD, n = 2 technical replicates per assay, with assays performed in triplicate.

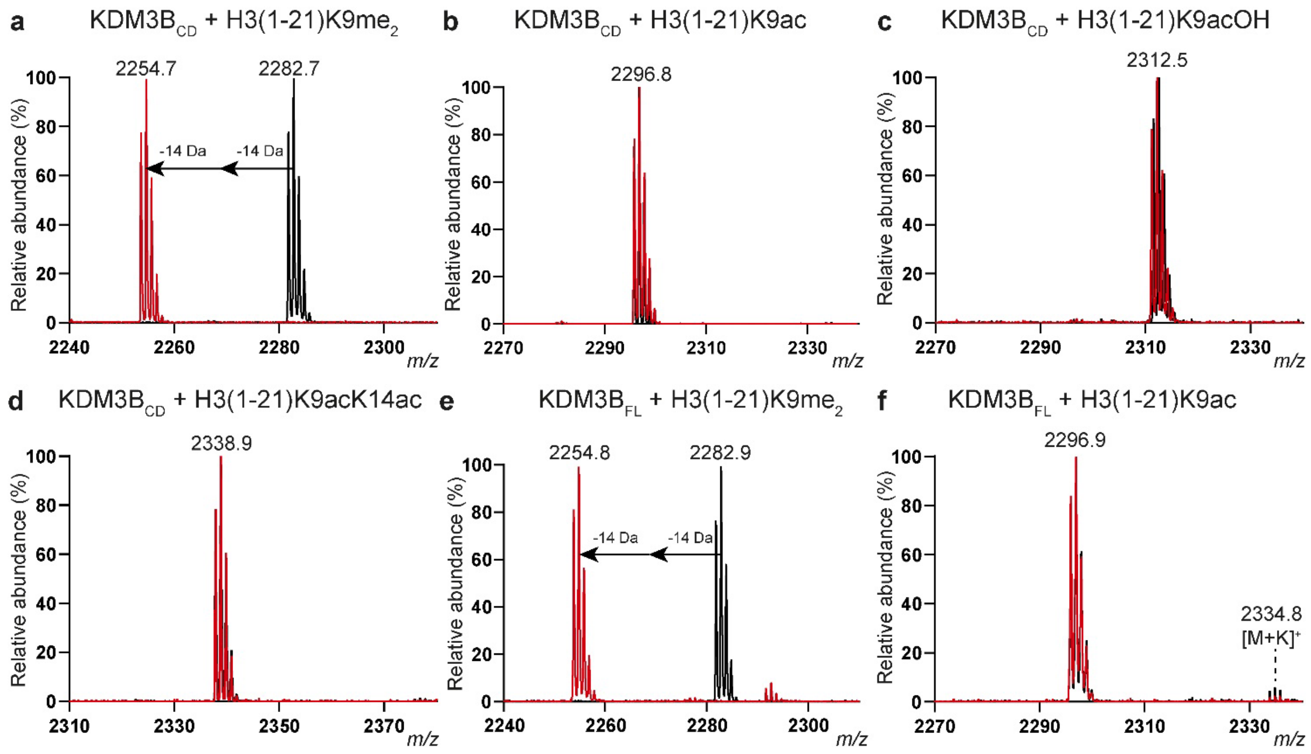

Supplementary Fig. 6 | Catalytic domain or full length recombinant KDM3B catalysed demethylation of H3(1-21)K9me$_2$, but no oxidation is observed with the other tested histone H3 lysine substrates. Recombinant KDM3B$_{CD}$ (catalytic domain only) or KDM3B$_{FL}$ (full length) was incubated with histone H3 peptides. Products were analysed using MALDI-TOF MS. Representative spectra are shown for each peptide (n = 2–3 data points). A mass shift of -14 Da indicates demethylation while a +16 Da shift indicates hydroxylation. Conditions: KDM3B$_{CD/FL}$ (0.5 µM), peptide (10 µM), L-ascorbate (500 µM), Fe(II) (50 µM), 2OG (100 µM), and TCEP (500 µM) were reacted at 37 °C (60 min). Black t: 0 min, red t: 60 min.

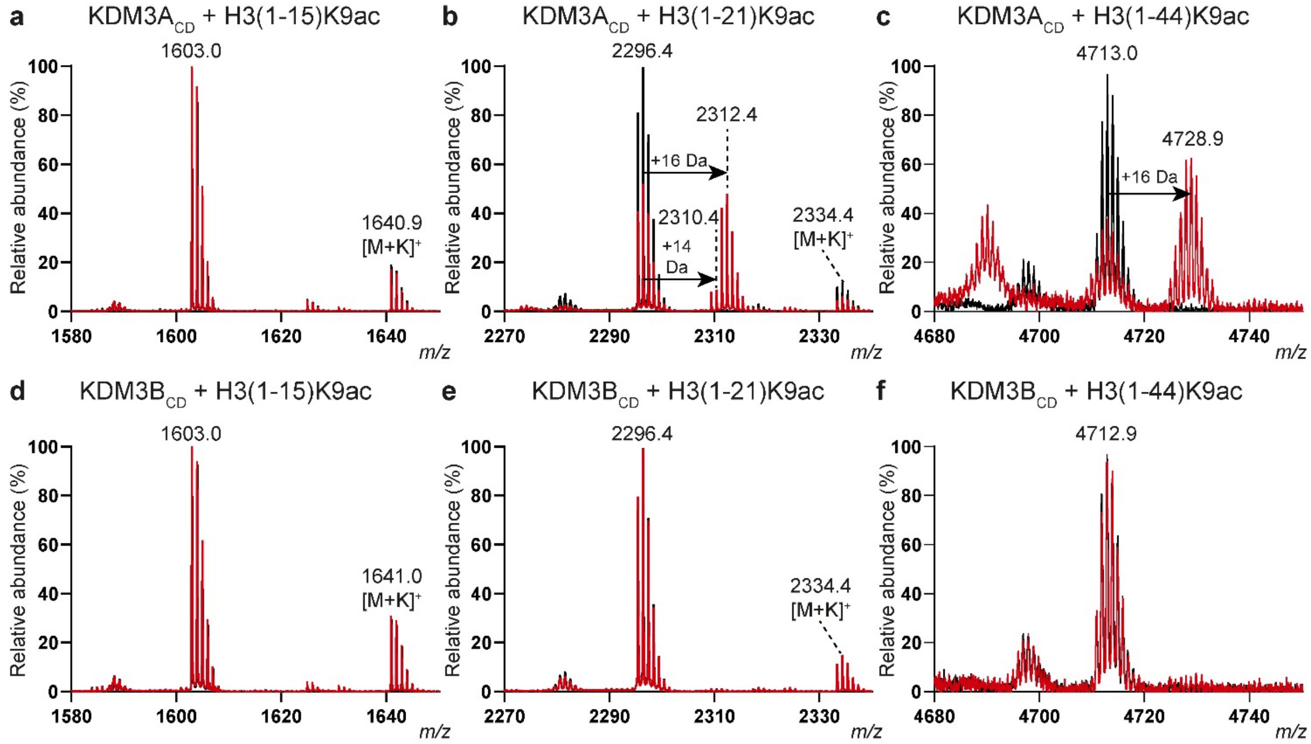

Supplementary Fig. 7 | Screening of hydroxylation of H3K9ac with different lengths of histone H3 catalysed by KDM3A or KDM3B. Recombinant KDM3ACD or KDM3BCD were incubated with modified N^{ε}-lysines 9 in histone H3 with analysis by MALDI-TOF MS. Representative spectra are shown for histone H3 peptides: **a,d** H3(1-15)K9ac; **b,e** H3(1-21)K9ac and **c,f** H3(1-44)K9ac with **a–c** KDM3ACD or **d–f** KDM3BCD. A mass shift of +16 Da indicates hydroxylation. Standard conditions: KDM3ACD/3BCD (0.5 µM), peptide (10 µM), L-ascorbate (500 µM), Fe(II) (50 µM), 2OG (100 µM), and TCEP (500 µM) (60 min, 37 °C). Black t: 0 min, red t: 60 min. (n: 3 data points per screen, two independent assays).

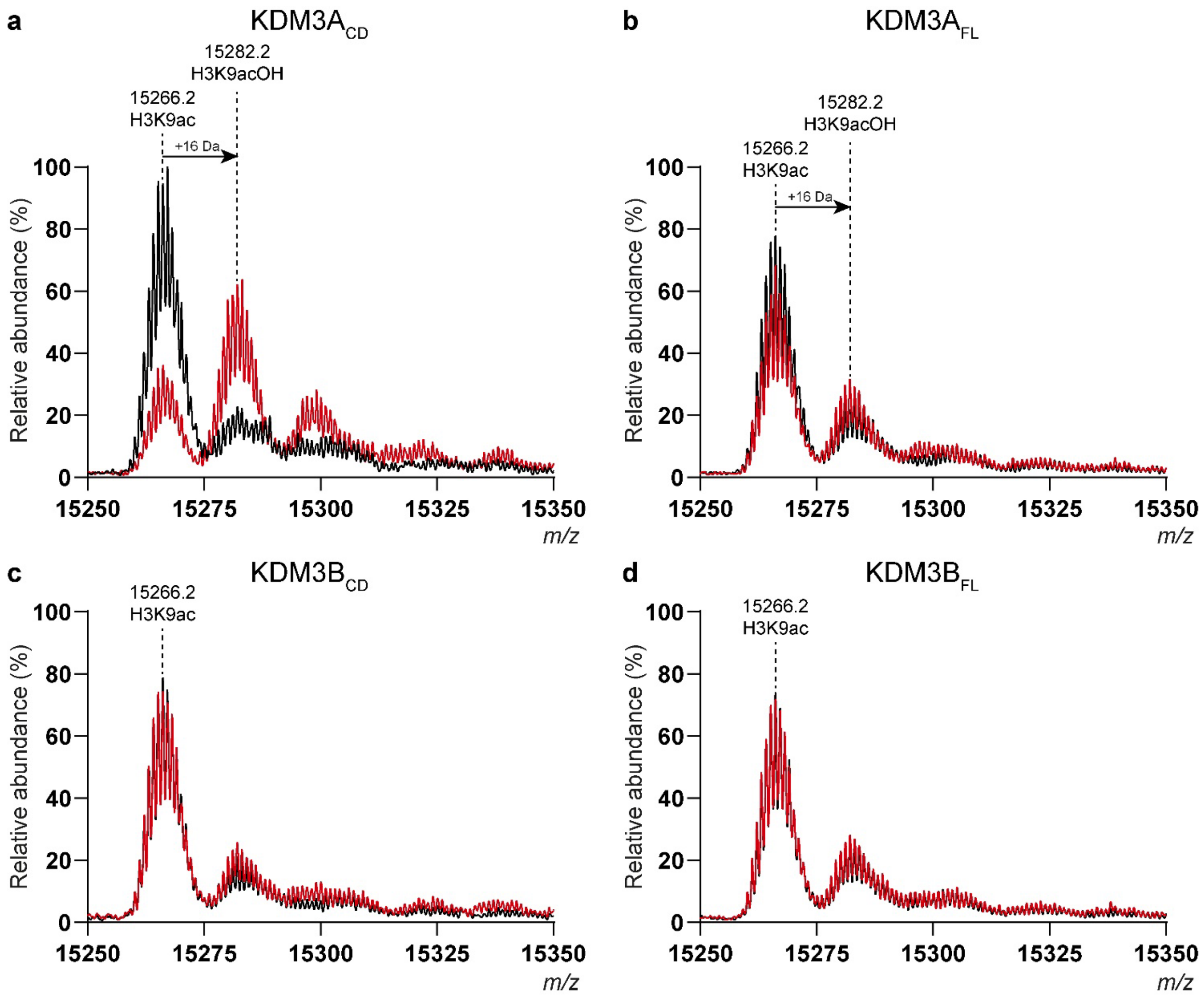

Supplementary Fig. 8 | Evidence for KDM3A catalysed oxidation of recombinant intact histone H3.2K9ac as determined using LC-MS. Intact histone H3.2K9ac was incubated with: **a** KDM3A$_{CD}$; **b** KDM3A$_{FL}$; **c**: KDM3B$_{CD}$; or **d** KDM3A$_{FL}$ enzyme. Conditions: KDM3A$_{CD}$ (0.5 µM), recombinant histone H3K9ac (10 µM), L-ascorbate (500 µM), Fe(II) (50 µM), 2OG (100 µM), and TCEP (500 µM) were incubated (60 min, 37 °C). Black: 0 min, red: 60 min. KDM3A$_{CD}$ and KDM3B$_{CD}$ n= 3 technical replicates; KDM3A$_{FL}$ and KDM3B$_{FL}$ (n= 2 technical replicates).

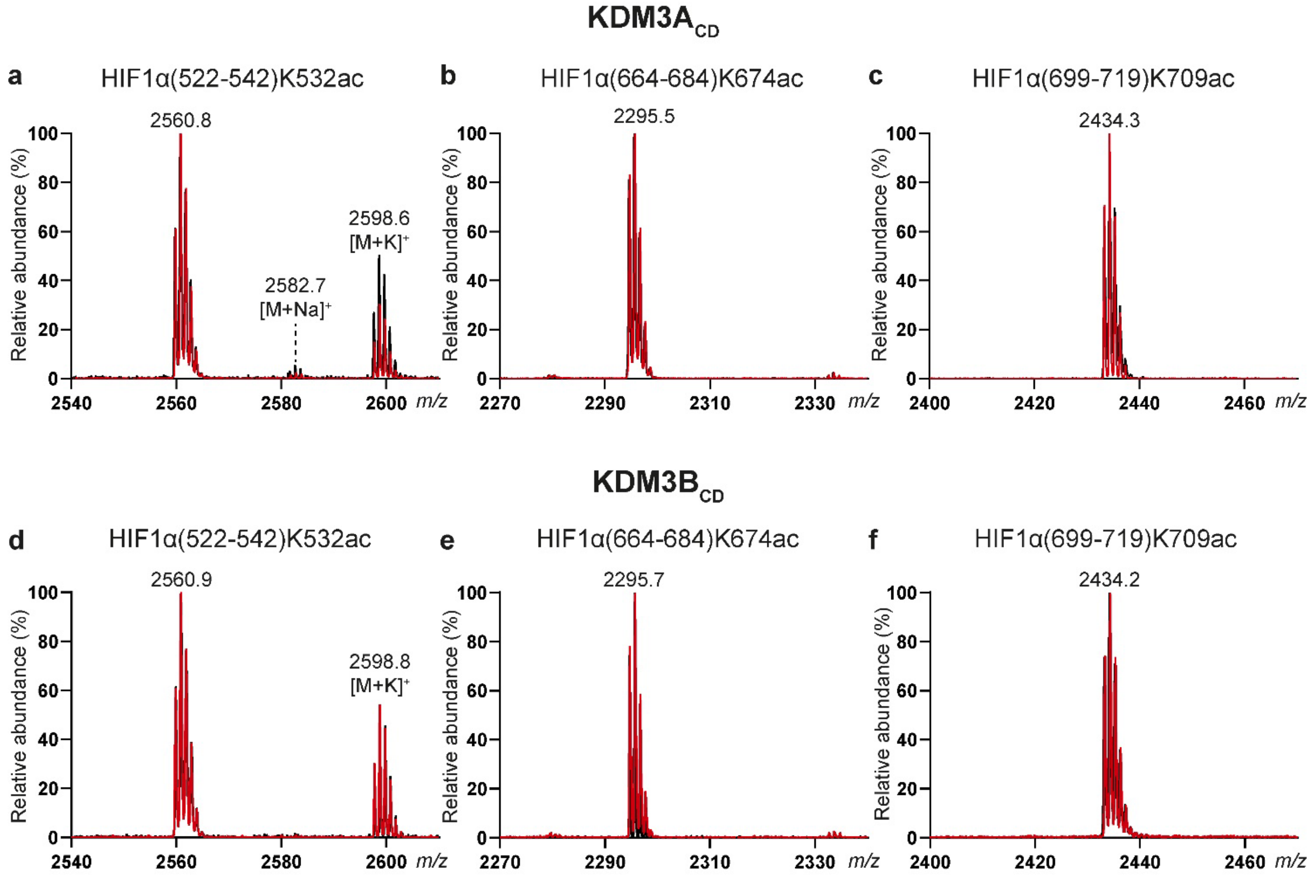

Supplementary Fig. 9 | Screening of histone H3 peptides containing modified N^{ε}-lysines as potential substrates for KDM3A. Recombinant KDM3A$_{CD}$ was incubated with modified N^{ε}-lysines in HIF1α peptides. Substrates used are given above the spectra. Reactions were analysed using MALDI-TOF MS. Representative MS spectra are shown for each peptide (n: 3 data points, two independent assays). A mass shift of +16 Da indicates hydroxylation. Conditions: KDM3A$_{CD}$ (0.5 µM), peptide (10 µM), L-ascorbate (500 µM), Fe(II) (50 µM), 2OG (100 µM), and TCEP (500 µM) (60 min, 37 °C). Black t: 0 min, red t: 60 min.

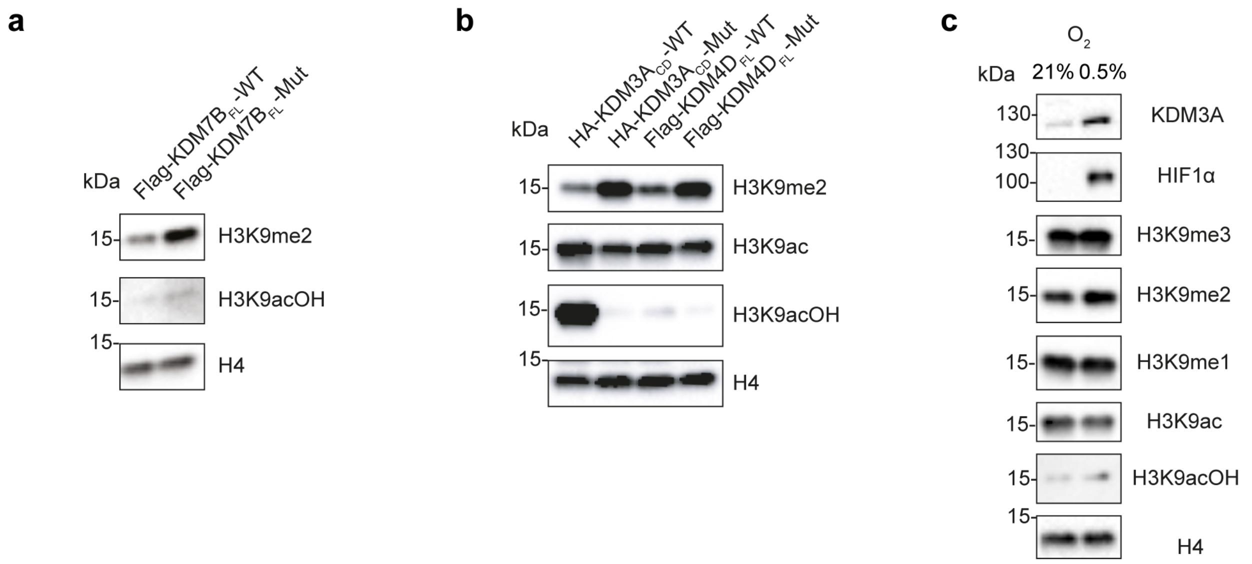

Supplementary Fig. 10 | Western blots of whole cell lysates from HEK293T cells transfected or grown in hypoxia. Western blot of whole cell lysate from HEK293T cells transiently transfected with KDM active (WT) or inactive (Mut) constructs: **a.** Flag-tagged human KDM7B (Flag-KDM7B$_{CD}$-WT/Mut), **b** Flag-tagged human KDM4D (Flag-KDM4D$_{FL}$-WT/Mut)**,** HA-tagged human KDM3A$_{CD}$ (HA-KDM3A$_{CD}$-WT/Mut). Cell lysates were analysed using anti-histone H3K9me2, H3K9ac, H3K9acOH, H4 antibodies. **c.** Western blots of HEK293T cell lysates grown under normoxia (21% O_2) or hypoxia (0.5% O_2) for 24 h. Cell lysates were analysed using histone PTM specific antibodies, KDM3A and HIF1α antibodies; anti-Histone H4 was used as loading controls.

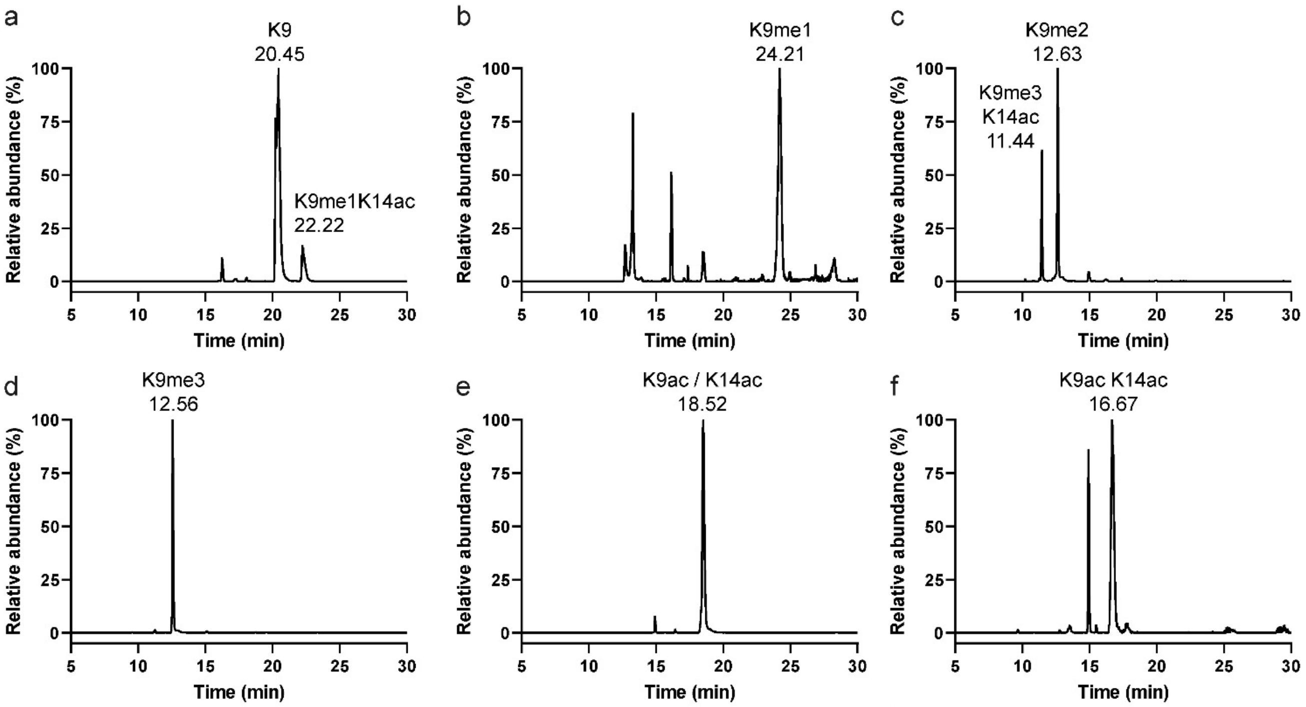

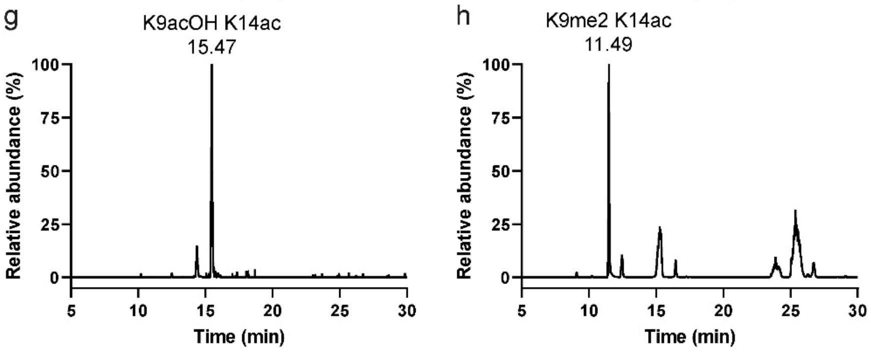

i

K9	K14	*m/z*
K9pr	K14pr	535.3032
K9me1, pr	K14pr	542.3109
K9me2	K14pr	521.3058
K9me3	K14pr	528.3136
K9ac or K9pr	K14pr or K14ac	528.2954
K9ac	K14ac	521.2877
K9acOH	K14ac	529.2853
K9me1, pr	K14ac	535.3033
K9me2	K14ac	514.2978
K9me3	K14ac	521.3061

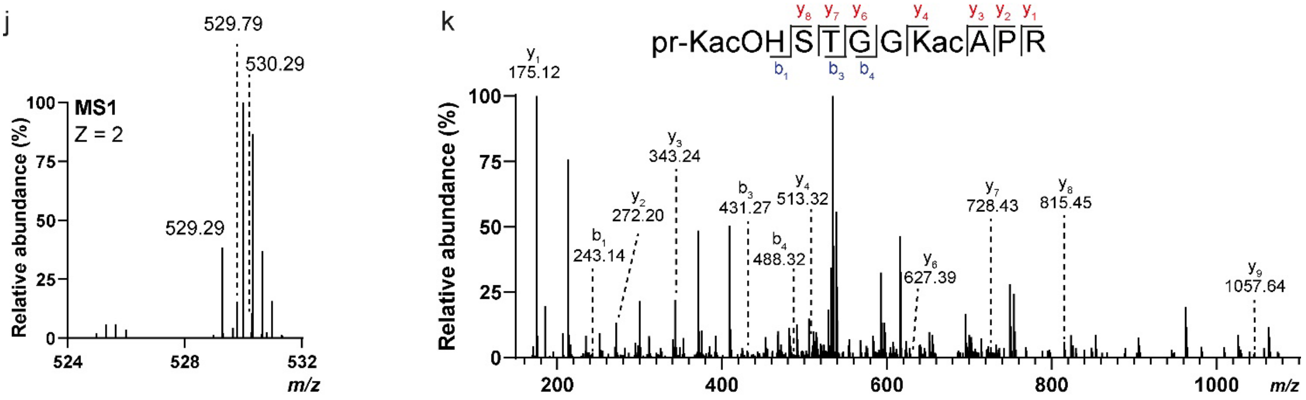

Supplementary Fig. 11 | LC-MS/MS of histone H3(9-17) ion fragments from HEK293T cell extracts overexpressing HA-KDM3A$_{CD}$-WT. Histones were extracted from HEK293T cells transfected with HA-KDM3A$_{CD}$-WT, then propionylated, then digested with trypsin. Histone H3(9-17) fragments were analysed using LC-MS/MS with mass ion extracted retention spectra from: (**a**) K9; (**b**) K9me1; (**c**) K9me2 and K9me3 K14ac; (**d**) K9me3; (**e**) K9ac or K14ac; (**f**) K9ac K14ac; (**g**) K9acOH K14ac; (**h**) and K9me2 K14ac. **i** Masses of extracted ions (MS1, z = 2) corresponding to spectra (**a**–**h**). **j** MS1 and (**k**) MS2 of H3(9-17) K9acOH K14ac fragment. Note that peptide fragments are propionylated at the N-terminus. Representative data from three biological repeats are shown.

2 Supplementary Tables

Supplementary Table 1 | Overview of recombinant Jumonji C domain-containing histone lysine demethylases (JmjC-KDMs), histone lysine deacetylases (HDACs) and histone lysine acetyltransferase (HAT) constructs used in this work. SGC: Structural Genomic Consortium.

	Synonym	Name in paper	Residues		Vector	Tag	Reference
			First	Last			
**Histone lysine demethylases**							
KDM3A	JMJD1A	KDM3A$_{CD}$	T515	S1317	pFB-CT10HF-LIC	*C*-term 10xHis/ FLAG	Rose *et al.*[1]
		KDM3A$_{FL}$	M1	P1321	Not specified	*N*-term FLAG	Active Motif 31456
KDM3B	JMJD1B	KDM3B$_{CD}$	L879	S1761	pFB-LIC-Bse	No	SGC
		KDM3B$_{FL}$	M1	S1761	Not specified	*N*-term FLAG	Active Motif 31429
JMJD1C	KDM3C	JMJD1C$_{CD}$	R1760	N2540	pFB-LIC-Bse	No	
KDM4A	JMJD2A	KDM4A$_{CD}$	M1	L359	pNIC28-Bsa4	*N*-term 6xHis	Ng *et al.*[2]
KDM4D	JMJD2D	KDM4D$_{CD}$	M1	L358	pNIC28-Bsa4	*N*-term 6xHis	Belle *et al.*[3]
KDM4E	JMJD2E	KDM4E$_{CD}$	M1	Q337	pNIC28-Bsa4	*N*-term 6xHis	Rose et al.[4]
KDM5A	JARID1A	KDM5A$_{CD}$	M1	L801	pFB-LIC-Bse	*N*-term 6xHis	Johansson *et al.*[5]
KDM5D	JARID1D	KDM5D$_{CD}$	M1	D775	pFB-LIC-Bse	*N*-term 6xHis	Johansson *et al.*[5]
KDM7B	PHF8	KDM7B$_{CD}$	M37	N483	pNH-TrxT	No	Rose *et al.*[1]
**Histone lysine acetyl transferase**							
KAT2B	PCAF	KAT2B$_{HAT}$	V493	E658	pNIC-Bio3	No	
**Histone lysine deacetylases**							
SIRT1	Sirtuin 1	SIRT1$_{CD}$	E82	S747	pQE-80	*N*-term 6xHis	Hallows *et al*[6] Addgene #13735
HDAC8	KDAC8	HDAC8$_{FL}$	M1	V377	pNIC28-Bsa4	No	
**YEATS domain**							
AF9	MLLT3, YEATS3	AF9	M1	S149	Not specified	*N*-term 6xHis	EpiCypher 15-0071

Supplementary Table 2 | Tandem MS (MS/MS) fragment ions for N^{ε}-acetyl-lysine 9 and N^{ε}-hydroxyacetyl-lysine 9 containing H3(1-21) peptides. Recombinant KDM3A$_{CD}$ was incubated with H3(1-21)K9ac (60 min). MS/MS spectra were obtained using MALDI-TOF/TOF MS. Calculated MS/MS a-, b-, and y-ions fragments of: (**a**) H3(1-21)K9ac and (**b**) H3(1-21)K9acOH are given, together with ion fragments experimentally observed for the substrate H3(1-21)K9ac (0 min) and product H3(1-21)K9acOH (60 min incubation with KDM3A$_{CD}$). Conditions: KDM3A$_{CD}$ (0.5 µM), H3(1-21)K9ac (10 µM), L-ascorbate (500 µM), Fe(II) (50 µM), 2OG (100 µM), and TCEP (500 µM) (60 min, 37 °C). Corresponding MS/MS spectra are given in **Supplementary Fig. 3**. Calculated ion fragments in red are those that match the experimentally observed ion fragments (within ±1 Da tolerance).

a

		Ala	Arg	Thr	Lys	Gln	Thr	Ala	Arg	Lys(ac)	Ser	Thr
Ion		1	2	3	4	5	6	7	8	9	10	11
a	Calculated	44.049	200.151	301.198	429.293	557.352	658.399	729.437	885.538	1055.643	1142.675	1243.723
	Found			301.464	429.584	557.729	658.814	729.864	885.970	1056.169		1244.295
b	Calculated	72.044	228.145	329.193	457.288	585.347	686.394	757.432	913.533	1083.638	1170.670	1271.718
	Found		228.381	329.479	457.614	585.704	686.793	757.847	914.008	1084.137	1171.200	1272.265
y	Calculated	89.071	202.155	330.214	458.309	614.410	711.462	782.500	910.594	967.616	1024.637	1125.685
	Found			330.480	458.611	614.760	711.862	782.921	911.056	968.095	1025.138	1126.208
		Ala	Leu	Gln	Lys	Arg	Pro	Ala	Lys	Gly	Gly	Thr

		Gly	Gly	Lys	Ala	Pro	Arg	Lys	Gln	Leu	Ala
Ion		12	13	14	15	16	17	18	19	20	21
a	Calculated	1300.744	1357.766	1485.861	1556.898	1653.951	1810.052	1938.147	2066.205	2179.289	2249.343
	Found	1301.314		1486.650	1557.557						
b	Calculated	1328.739	1385.761	1513.856	1584.893	1681.946	1838.047	1966.142	2094.200	2207.284	2277.337
	Found	1329.321	1386.368	1514.495	1585.552	1682.633	1838.780	1966.895	2094.973		
y	Calculated	1212.717	1382.823	1538.924	1609.961	1711.009	1839.067	1967.162	2068.210	2224.311	2295.348
	Found	1213.264	1383.426	1539.574	1610.636	1711.707	1839.755	1967.909	2068.986		
		Ser	Lys(ac)	Arg	Ala	Thr	Gln	Lys	Thr	Arg	Ala

b

		Ala	Arg	Thr	Lys	Gln	Thr	Ala	Arg	Lys(acOH)	Ser	Thr
Ion		1	2	3	4	5	6	7	8	9	10	11
a	Calculated	44.049	200.151	301.198	429.293	557.352	658.399	729.437	885.538	1071.638	1158.67	1259.718
	Found		200.343	301.429	429.541	557.676	658.751	729.794	885.97	1072.088		1260.207
b	Calculated	72.044	228.145	329.193	457.288	585.347	686.394	757.432	913.533	1099.633	1186.665	1287.713
	Found		228.358	329.445	457.563	585.647	686.729	757.774	913.933	1100.053	1187.113	1288.182
y	Calculated	89.071	202.155	330.214	458.309	614.41	711.462	782.5	910.594	967.616	1024.637	1125.685
	Found		202.337	330.464	458.569	614.712	711.803	782.855	910.98	968.022	1025.058	1126.12
		Ala	Leu	Gln	Lys	Arg	Pro	Ala	Lys	Gly	Gly	Thr

		Gly	Gly	Lys	Ala	Pro	Arg	Lys	Gln	Leu	Ala
Ion		12	13	14	15	16	17	18	19	20	21
a	Calculated	1316.739	1373.761	1501.856	1572.893	1669.946	1826.047	1954.142	2082.2	2195.284	2265.337
	Found				1573.452						
b	Calculated	1344.734	1401.756	1529.851	1600.888	1697.941	1854.042	1982.137	2110.195	2223.279	2293.332
	Found	1345.229	1402.277	1530.391	1601.488		1854.664	1982.791	2110.861		
y	Calculated	1212.717	1398.818	1554.919	1625.956	1727.003	1855.062	1983.157	2084.205	2240.306	2311.343
	Found	1213.179	1399.333	1555.473	1626.532	1727.596	1855.663	1983.801	2084.865		
		Ser	Lys(acOH)	Arg	Ala	Thr	Gln	Lys	Thr	Arg	Ala

Supplementary Table 3 | Overview of cellular constructs used in this work. HA: Hemagglutinin and nuclear localisation sequence (HA-NLS).

Protein	Synonym	Name in paper	Residues First	Residues Last	Tag	Mutation	Reference
		**Histone lysine demethylases**					
KDM3A	JMJD1A	HA-KDM3A$_{CD}$-WT	511	1321	HA-NLS		
		HA-KDM3A$_{CD}$-Mut	511	1321	HA-NLS	H1120Y	
		Flag-KDM3A$_{FL}$-WT	1	1321	Flag		
		Flag-KDM3A$_{FL}$-Mut	1	1321	Flag	H1120Y	
KDM3B	JMJD1B	HA-KDM3B$_{CD}$-WT	879	1761	HA-NLS		
		HA-KDM3B$_{CD}$-Mut	879	1761	HA-NLS	H1560A & D1562A	
JMJD1C	KDM3C	HA-JMJD1C$_{CD}$-WT	1696	2540	HA-NLS		
		HA-JMJD1C$_{CD}$-Mut	1696	2540	HA-NLS	H2336A & E2338A	
KDM4D	JMJD2D	Flag-KDM4D$_{FL}$-WT	1	523	3×Flag		
		Flag-KDM4D$_{FL}$-Mut	1	523	3×Flag	H192A & E194A	
KDM7B	PHF8	Flag-KDM7B$_{CD}$-WT	1	489	3×Flag		
		Flag-KDM7B$_{CD}$-Mut	1	489	3×Flag	H247A & D249A	

Supplementary Table 4 | Mass spectrometry MS2 fragmentation of histone H3(9-17)K9acOH and H3(9-17)K9acOH K14ac observed from HEK293T cell extracts overexpressing HA-KDM3A$_{CD}$-WT. Histones were extracted from HEK293T cells transfected with HA-KDM3A$_{CD}$-WT, propionated and digested using trypsin. Histone fragments were analysed using HPLC-MS/MS observing b- and y-ions. Note that peptide fragments are propionylated at the N-terminus. Representative data from three biological repeats are shown. Calculated ion fragments in red for those that match the experimentally observed ion fragments (within ±0.1 Da tolerance).

		KacOH 1	Ser 2	Thr 3	Gly 4	Gly 5	Lys14pr 6	Ala 7	Pro 8	Arg 9
b-ion	Calculated	243.14	330.17	431.22	488.24	545.26	729.38	800.42	897.47	1053.57
	Found	243.13	330.17		488.25	545.28		800.44	897.51	
y-ion	Calculated	175.12	272.17	343.21	527.33	584.35	641.37	742.42	829.45	1071.58
	Found	175.12	272.17	343.21	527.31	584.35	641.37	742.42	829.45	
		Arg 1	Pro 2	Ala 3	Lys(pr) 4	Gly 5	Gly 6	Thr 7	Ser 8	KacOH 9

		K9acOH 1	Ser 2	Thr 3	Gly 4	Gly 5	K14ac 6	Ala 7	Pro 8	Arg 9
b-ion	Calculated	243.14	330.17	431.22	488.24	545.26	715.37	786.40	883.45	1039.56
	Found	243.14		431.27	488.32					
y-ion	Calculated	175.12	272.17	343.21	513.31	570.34	627.36	728.41	815.44	1057.57
	Found	175.12	272.20	343.24	513.32		627.39	728.43	815.45	1057.64
		Arg 1	Pro 2	Ala 3	Lys(pr) 4	Gly 5	Gly 6	Thr 7	Ser 8	KacOH 9

Supplementary Table 5 | Antibodies and chromatin amount used for chromatin immunoprecipitation studies.

Target	Source	Identifier	Volume of Ab / IP	Chromatin / IP
**H3K9acOH**	In house	H3K9acOH	30.8 µL	50 µg
**H3K9ac**	Abcam	ab4441	8 µL	5 µg
**H3K4me3**	CST	9751S	15 µL	5 µg
**Total H3**	Abcam	ab1791	15 µL	5 µg
**Drosophila spike-in (histone variant H2Av)**	Active Motif	61686	1.0 µL	10 ng of spike-in Drosophila chromatin

3 Synthesis of amino acid monomers

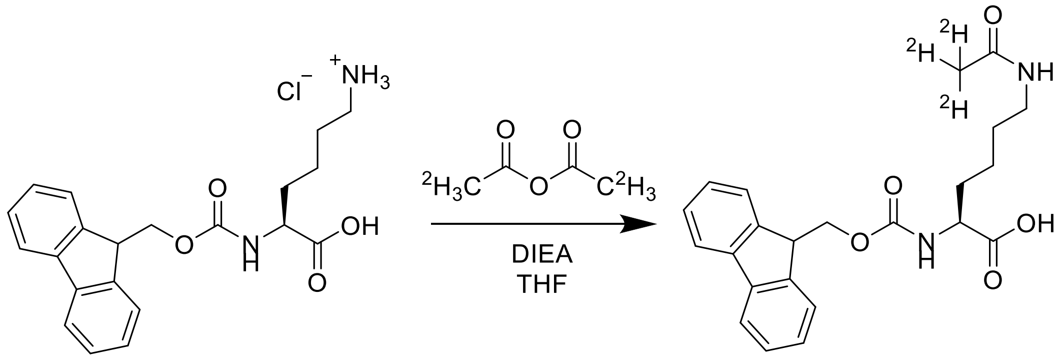

(*S*)-2-[Fluorenylmethyloxycarbonyl]amino-6-[acetyl-$^2H_3$]amino-hexanoic acid [7]

Fmoc-Lys(ac-$^2H_3$)-OH

The desired compound was produced following a reported procedure with minor modifications[7]. A suspension of Fmoc-Lys(HCl)-OH (797 mg, 1.97 mmol, 1.0 equiv.) in anhydrous THF (10 mL, 0.2 M) was cooled to 0 °C under a nitrogen atmosphere with mechanical stirring. Acetic anhydride-[$^2H_6$] (208 µL, 2.2 mmol, 1.12 equiv.) was added dropwise, followed by the dropwise addition of diisopropylethylamine (DIEA) (490 µL, 2.8 mmol, 1.42 equiv.). The ice-bath was removed and the mixture was then allowed to gradually warm to room temperature with stirring for 2 h. After TLC analysis indicated completion of reaction, the solution was quenched with aqueous $KHSO_4$ (0.1 M, 25 mL) and ethyl acetate was added (~100 mL). The organic layer was separated, washed with aqueous $KHSO_4$ (0.1 M, 25 mL), then brine (25 mL), dried (Na_2SO_4), filtrated, and concentrated. The crude material was purified by reverse phase (R_f-C_{18}) chromatography using a Biotage machine. (3CV 5% B, 5% to 100% B over 20 CV. A= [0.01%$_{v/v}$ TFA in MilliQ], B= [0.01%$_{v/v}$ TFA in MeCN]. Fractions were concentrated *in vacuo* then lyophilized to yield a white solid (267 mg, 1.83 mmol, 33%). IR υ_{max}: 3332, 2944, 1736, 1691, 1590, 1550, 1526, 1211, 1188, 1086, 1032, 757, 741, 620, 595, 531 cm^{-1}. ^{1}H-NMR (500 MHz in DMSO-$^2H_6$) δ (ppm): 12.56 (bs, 1×H, COO**H**), 7.90 (d, 2×H, *J*= 7.5 Hz, Fmoc-C$_4$**H**), 7.80 (bt, 1×H, *J*= 5.5 Hz, $^\varepsilon$CH$_2$N**H**COC^{2}H$_3$), 7.74 (d, 2×H, *J*= 7.5, 2×Fmoc-C$_1$**H**), 7.62 (d, 1×H, *J*= 8.0, N**H**$^\alpha$CH), 7.43 (t, 2×H, *J*= 7.5 Hz, 2×Fmoc-C$_3$**H**), 7.34 (dt, 2×H, *J*= 7.5 1.0 Hz, 2×Fmoc-C$_2$**H**), 4.33-4.18 (m, 3×H, OC**H**$_2$C**H**), 3.95-3.88 (m, 1×H, NH$^\alpha$C**H**COOH), 3.01 (q, 2×H, *J*= 6.5 Hz, $^\delta$CH$_2$$^\varepsilon$C**H**$_2$NH), 1.78-1.49 (m, 2×H, $^\alpha$CH$^\beta$C**H**$_2$$^\gamma$CH$_2$), 1.49-1.20 (m, 4×H, $^\beta$CH$_2$$^\gamma$C**H**$_2$$^\delta$C**H**$_2$$^\varepsilon$CH$_2$). ^{2}H-NMR (76.8 MHz in DMSO-H$_6$) δ (ppm): 3.31 (s, NHCOC**^{2}H**$_3$). ^{13}C-NMR (126 MHz in DMSO-^{2}H$_6$) δ (ppm): 174.0 ($^\alpha$CH**C**OOH), 168.9 (NH**C**OC^{2}H$_3$), 156.2 (O**C**ONH), 143.8 (C-**C**-CHCH$_2$), 140.7, (**C**-C-CHCH$_2$), 127.6 (Fmoc-C$_3$), 127.1 (Fmoc-C$_2$), 125.3 (Fmoc-C$_1$), 120.1 (Fmoc-C$_4$), 65.6 (CH**C**H$_2$NH$^\alpha$CH), 53.8 (NH$^\alpha$**C**HCOOH), 46.7 (**C**HCH$_2$NH$^\alpha$CH), 38.2 ($^\delta$CH$_2$$^\varepsilon$**C**H$_2$NH), 30.4 ($^\alpha$CH$^\beta$**C**H$_2$$^\gamma$CH$_2$), 28.7 ($^\gamma$CH$_2$$^\delta$**C**H$_2$$^\varepsilon$CH$_2$), 23.1 ($^\beta$CH$_2$$^\gamma$**C**H$_2$$^\delta$CH$_2$), 21.9 (NHCO**C**^{2}H$_3$). ESI-MS (in MeOH) as [M-H]$^-$ found: 412.2, calculated: 412.2. High resolution ESI-MS (in MeOH) as [M-H]$^-$ found: 412.1955,

calculated from $C_{23}H_{22}{}^{2}H_{3}N_{2}O_{5}^{-}$: 412.1952. Analytical data are consistent with those reported values[7]. NMR spectra can be found in **Supplementary Fig. 12-14** [8].

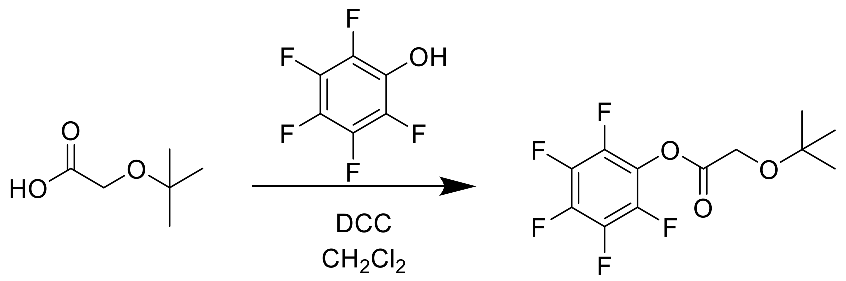

Perfluorophenyl-2-(tert-butoxy)-acetate

A solution of 2-(tert-butoxy)-acetic acid (1076 mg, 8.14 mmol, 1.1 equiv.) in dichloromethane (17 mL) was cooled to 0 °C, with mechanical stirring under a N_2 atmosphere. A solution of pentafluorophenol (1371 mg, 7.4 mmol, 1.1 equiv.) in dichloromethane (10 mL) was added dropwise followed by dicyclohexylcarbodiimide (1671 mg, 8.14 mmol, 1.1 equiv. in dichloromethane (10 mL, 0.2 M final concentration). The mixture was stirred at 0 °C for 60 min until TLC analysis indicated reaction completion. The mixture was passed through at Celite® pad, washed with dichloromethane (80 mL), and the supernatant was concentrated. Purification using a 50 g column (Biotage): 0% B for 1 CV followed by gradient of 0 to 15% B over 11 CV. [A: cyclohexane] + [B: ethyl acetate]. Selected fractions were pooled, then concentrated to give a colourless oil (1918 mg 87%). R_f: ~0.5 at 20% v/v B in A. ^{1}H-NMR (400 MHz in $CDCl_3$-$^2H_1$) δ (ppm): 4.39 (s, 2×H, C**<u>H</u>**$_2$C(CH$_3$)$_3$), 1.29 (s, 9×H, CH$_2$C(C**<u>H</u>**$_3$)$_3$). ^{19}F-NMR (376 MHz in $CDCl_3$-$^2H_1$) δ (ppm): -152.5 (d, 2×F, *J*= 17 Hz), -157.6 (t, 1×F, *J*= 21.5 Hz), -162.1 (m, 2×F). NMR spectra can be found in **Supplementary Fig. 15, 16**.

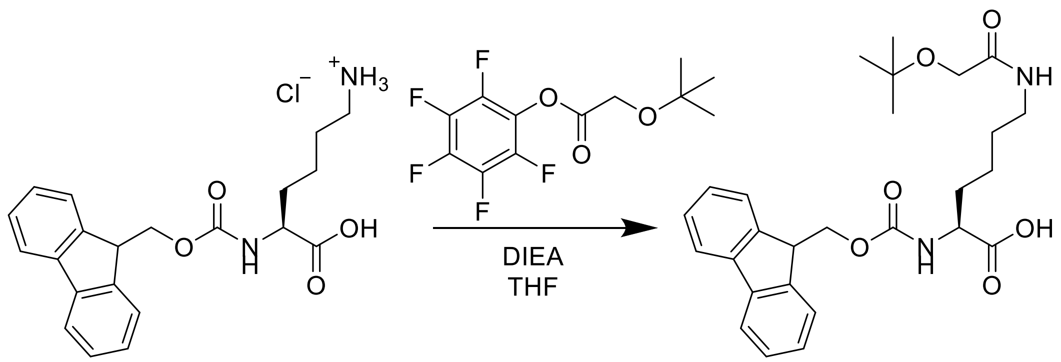

(*S*)-2-[Fluorenylmethyloxycarbonyl]amino-6-[2-(tertbutoxy)-acetyl]amino-hexanoic acid

Fmoc-Lys(acO^{t}Bu)-OH

To a solution pentafluorophenyl 2-(tert-butoxy)-acetate (1902 mg, 6.38 mmol, 1.2 equiv.) in anhydrous THF (53 mL, 0.1 M), was added Fmoc-Lys(HCl)-OH (2142 mg, 5.31 mmol, 1.0 equiv.), followed by slow dropwise addition of diisopropylethylamine (1.3 mL, 7.44 mmol, 1.4 equiv.). The mixture was stirred at room temperature for 17 h, when the white suspension had cleared and ESI-MS analysis indicated reaction completion. Ethyl acetate (100 mL) was added and the organic layer was washed with $NaHSO_4$ solution (50 mL, 1 M, pH 2.0), brine (50 mL), dried over sodium sulphate, then filtered. The resultant solution was concentrated, then loaded on a 50 g column (Biotage): $0\%_{v/v}$ B for 1 CV followed by gradient of 0 to $10\%_{v/v}$ B over 16 CV. [A: 0.1% acetic acid in CH_2Cl_2] + [B: $0.1\%_{v/v}$ acetic acid in MeOH]. Selected fractions were pooled, then concentrated to give a white solid (2503 mg 98%). IR υ_{max}: 3330, 2936, 1715, 1530, 1449, 1187, 1087, 759, 739, 621, 583, 542 cm^{-1}. ^{1}H-NMR (376 MHz in DMSO-$^{2}H_6$) δ (ppm): 12.57 (bs, 1×H, $COO\underline{\mathbf{H}}$), 7.90 (d, 2×H, *J*= 7.5 Hz, Fmoc-$C_4\underline{\mathbf{H}}$), 7.74 (d, 2×H, *J*= 7.5, 2×Fmoc-$C_1\underline{\mathbf{H}}$), 7.64 (d, 1×H, *J*= 8.0, $N\underline{\mathbf{H}}^{\alpha}CH$), 7.52 (bt, 1×H, *J*= 5.5 Hz, $^{\varepsilon}CH_2N\underline{\mathbf{H}}COC$), 7.43 (t, 2×H, *J*= 7.5 Hz, 2×Fmoc-$C_3\underline{\mathbf{H}}$), 7.34 (dt, 2×H, *J*= 7.5 1.0 Hz, 2×Fmoc-$C_2\underline{\mathbf{H}}$), 4.33-4.26 (m, 2×H, $OC\underline{\mathbf{H}}_2CH$), 4.26-4.18 (m, 1×H, $OCH_2C\underline{\mathbf{H}}$),3.95-3.87 (m, 1×H, $NH^{\alpha}C\underline{\mathbf{H}}COOH$), 3.74 (s, 2×H, $NHCOC\underline{\mathbf{H}}_2OC(CH_3)_3$), 3.15-3.04 (m, 2×H, $^{\delta}CH_2{}^{\varepsilon}C\underline{\mathbf{H}}_2NH$), 1.76-1.56 (m, 2×H, $^{\alpha}CH^{\beta}C\underline{\mathbf{H}}_2{}^{\gamma}CH_2$), 1.49-1.22 (m, 4×H, $^{\beta}CH_2{}^{\gamma}C\underline{\mathbf{H}}_2{}^{\delta}C\underline{\mathbf{H}}_2{}^{\varepsilon}CH_2$), 1.16 (s, 9×H, $NHCOCH_2OC(C\underline{\mathbf{H}}_3)_3$). ^{13}C-NMR (126 MHz in DMSO-$^{2}H_6$) δ (ppm): 174.5 ($^{\alpha}CH\underline{\mathbf{C}}OOH$), 170.3 ($^{\varepsilon}CH_2NH\underline{\mathbf{C}}OC$), 156.6 ($O\underline{\mathbf{C}}ONH$), 144.3 (C-$\underline{\mathbf{C}}$-$CHCH_2$), 141.2, ($\underline{\mathbf{C}}$-C-$CHCH_2$), 128.1 (Fmoc-$C_3$), 127.5 (Fmoc-$C_2$), 125.8 (Fmoc-$C_1$), 120.6 (Fmoc-$C_4$), 74.3 ($\underline{\mathbf{C}}(CH_3)_3$), 66.1 ($CH\underline{\mathbf{C}}H_2NH^{\alpha}CH$), 62.5 ($NHCO\underline{\mathbf{C}}H_2C(CH_3)_3$), 54.2 ($NH^{\alpha}\underline{\mathbf{C}}HCOOH$), 47.1 ($\underline{\mathbf{C}}HCH_2NH^{\alpha}CH$), 38.3 ($^{\delta}CH_2{}^{\varepsilon}\underline{\mathbf{C}}H_2NH$), 30.9 ($^{\alpha}CH^{\beta}\underline{\mathbf{C}}H_2{}^{\gamma}CH_2$), 29.3 ($^{\gamma}CH_2{}^{\delta}\underline{\mathbf{C}}H_2{}^{\varepsilon}CH_2$), 27.5 ($C(\underline{\mathbf{C}}H_3)_3$), 23.5 ($^{\beta}CH_2{}^{\gamma}\underline{\mathbf{C}}H_2{}^{\delta}CH_2$). ESI-MS (in MeOH) as $[M+H]^+$ found: 483.1, calculated: 483.2. High resolution ESI-MS (in MeOH) as $[M+H]^+$ found: 483.2516, calculated for $C_{27}H_{35}N_2O_6^+$: 483.2495. NMR spectra can be found in **Supplementary Fig. 17, 18**.

4 NMR spectra

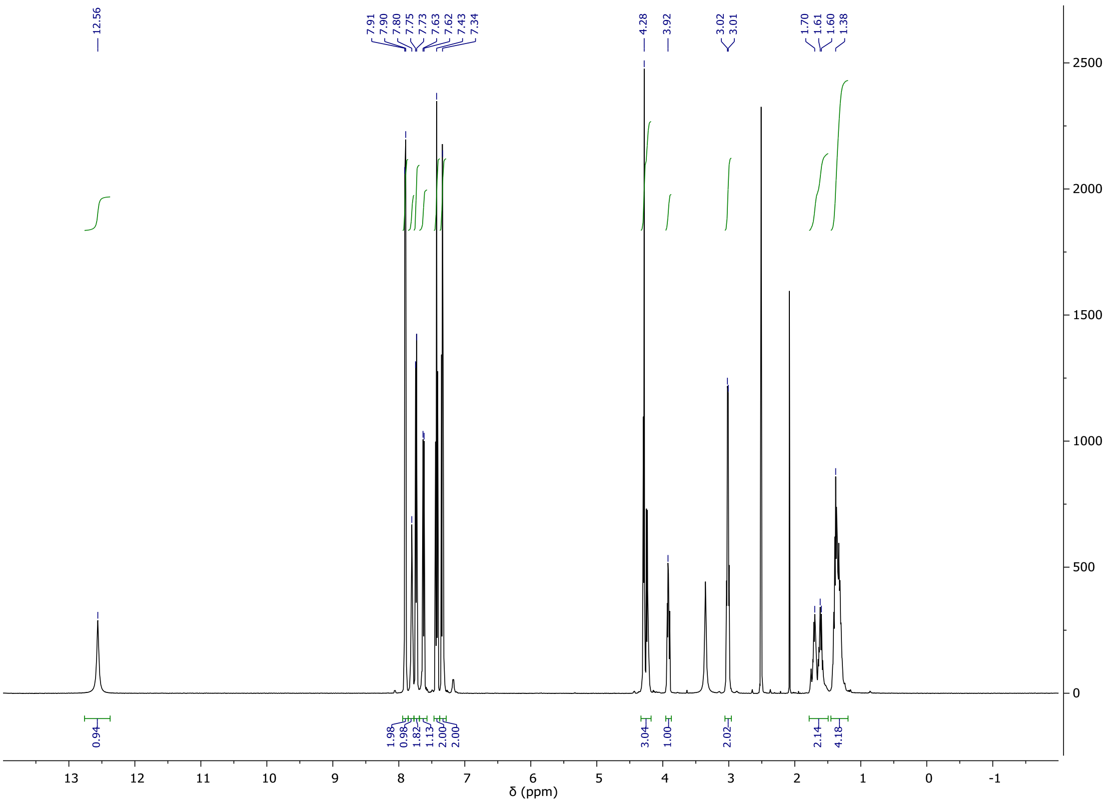

Supplementary Fig. 12 | ^{1}H-NMR spectrum of Fmoc-Lys(ac-$^2H_3$)-OH.

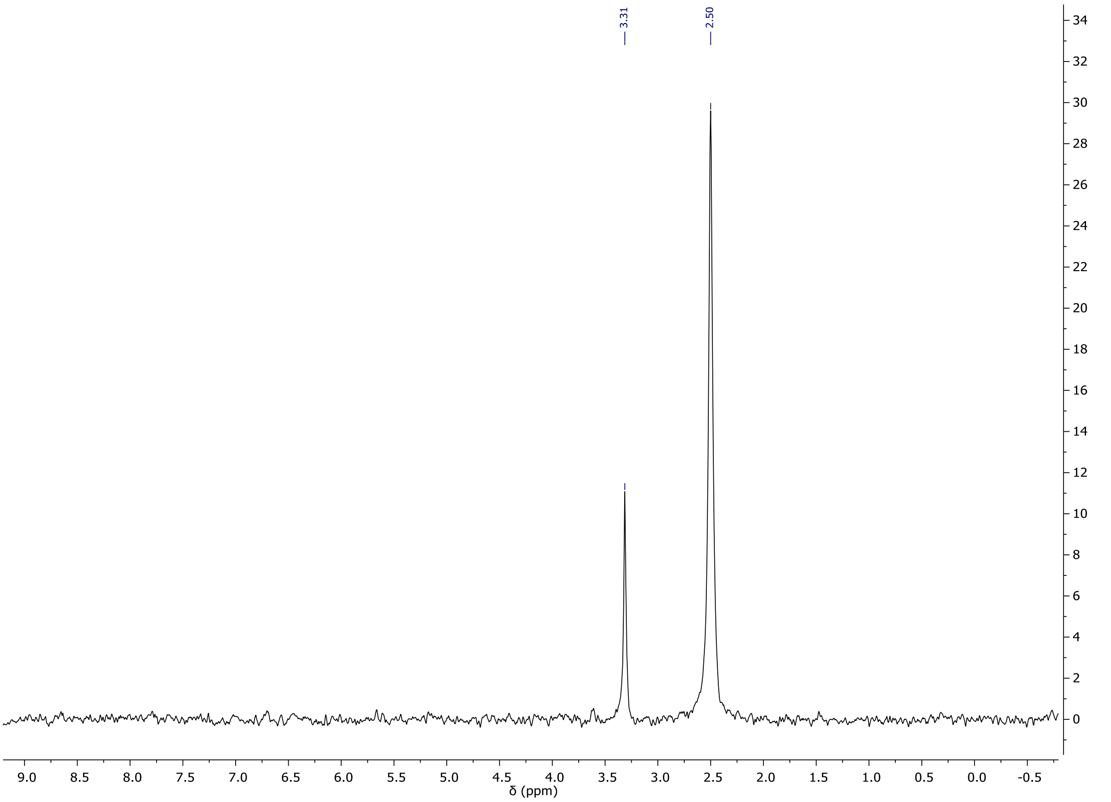

Supplementary Fig. 13 | ^{2}H-NMR spectrum of Fmoc-Lys(ac-$^2H_3$)-OH.

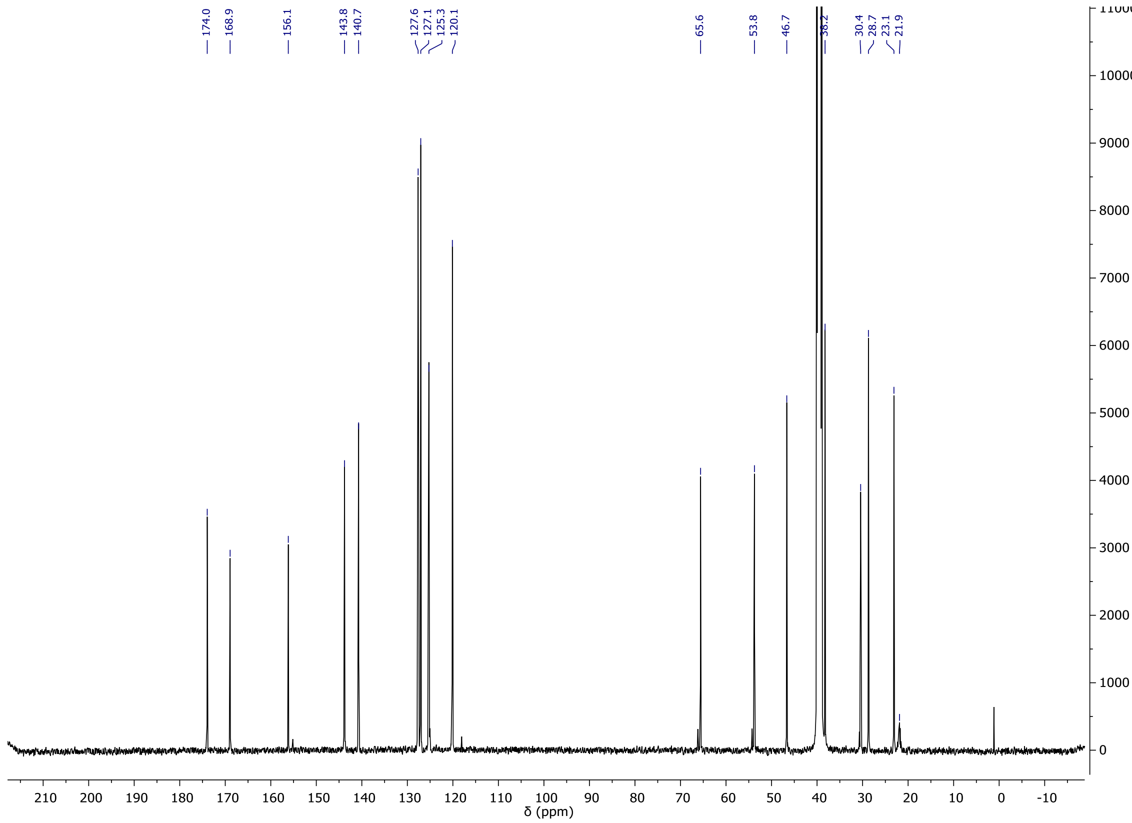

Supplementary Fig. 14 | ^{13}C-NMR spectrum of Fmoc-Lys(ac-$^{2}H_3$)-OH.

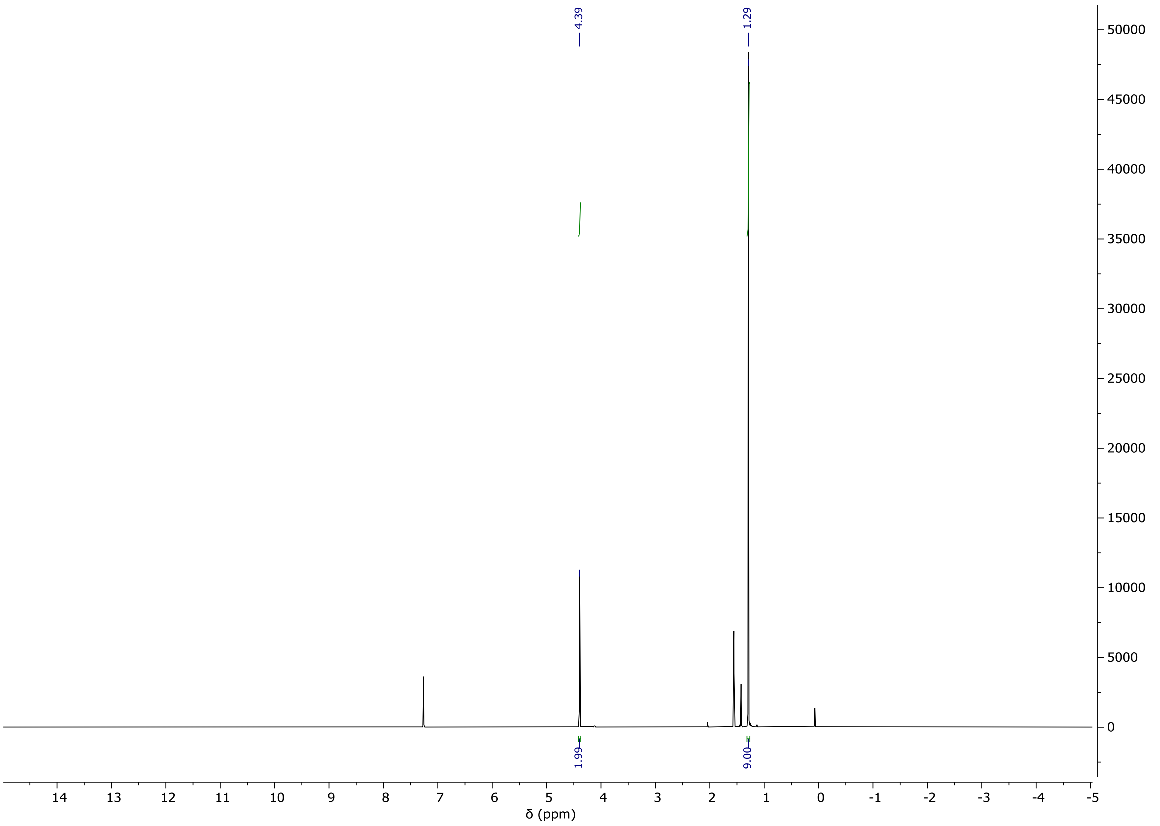

Supplementary Fig. 15 | ^{1}H-NMR spectrum of Perfluorophenyl 2-(tert-butoxy)-acetate.

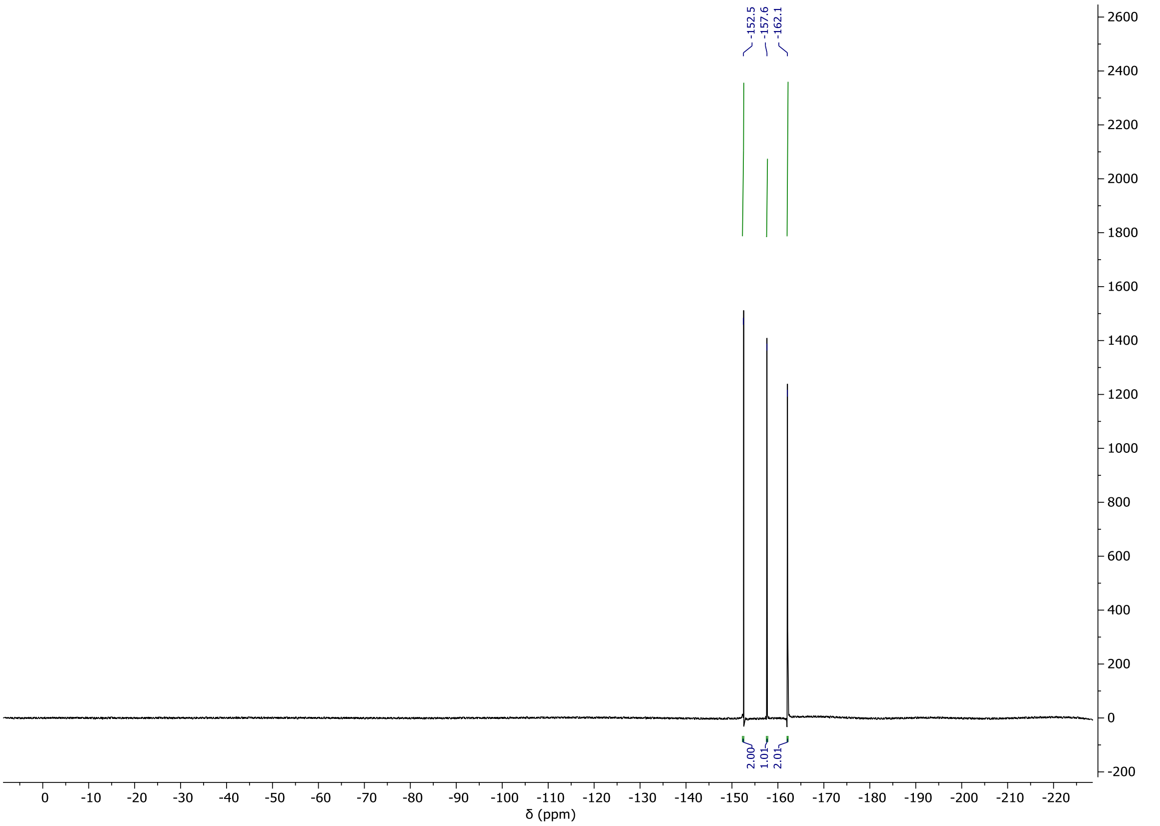

Supplementary Fig. 16 | ^{19}F-NMR spectrum of Perfluorophenyl 2-(tert-butoxy)-acetate.

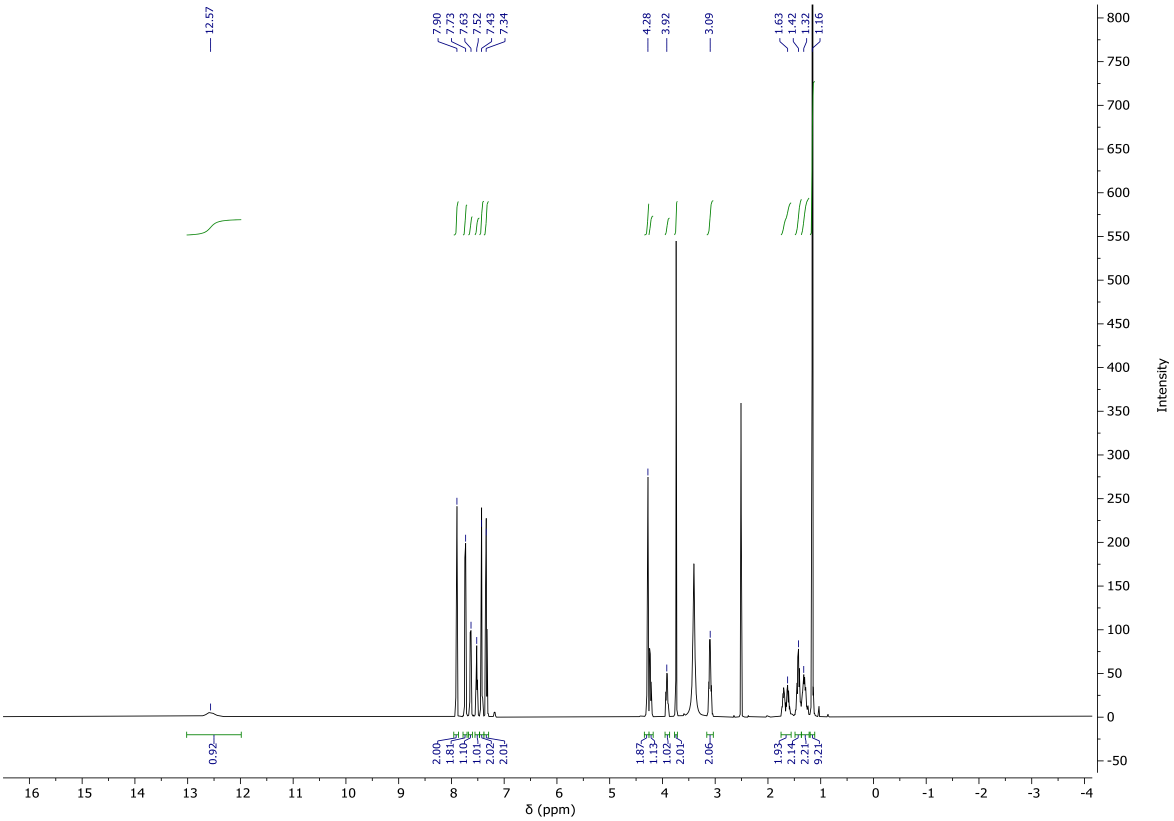

Supplementary Fig. 17 | ^{1}H-NMR spectrum of Fmoc-Lys(acO^{t}Bu)-OH.

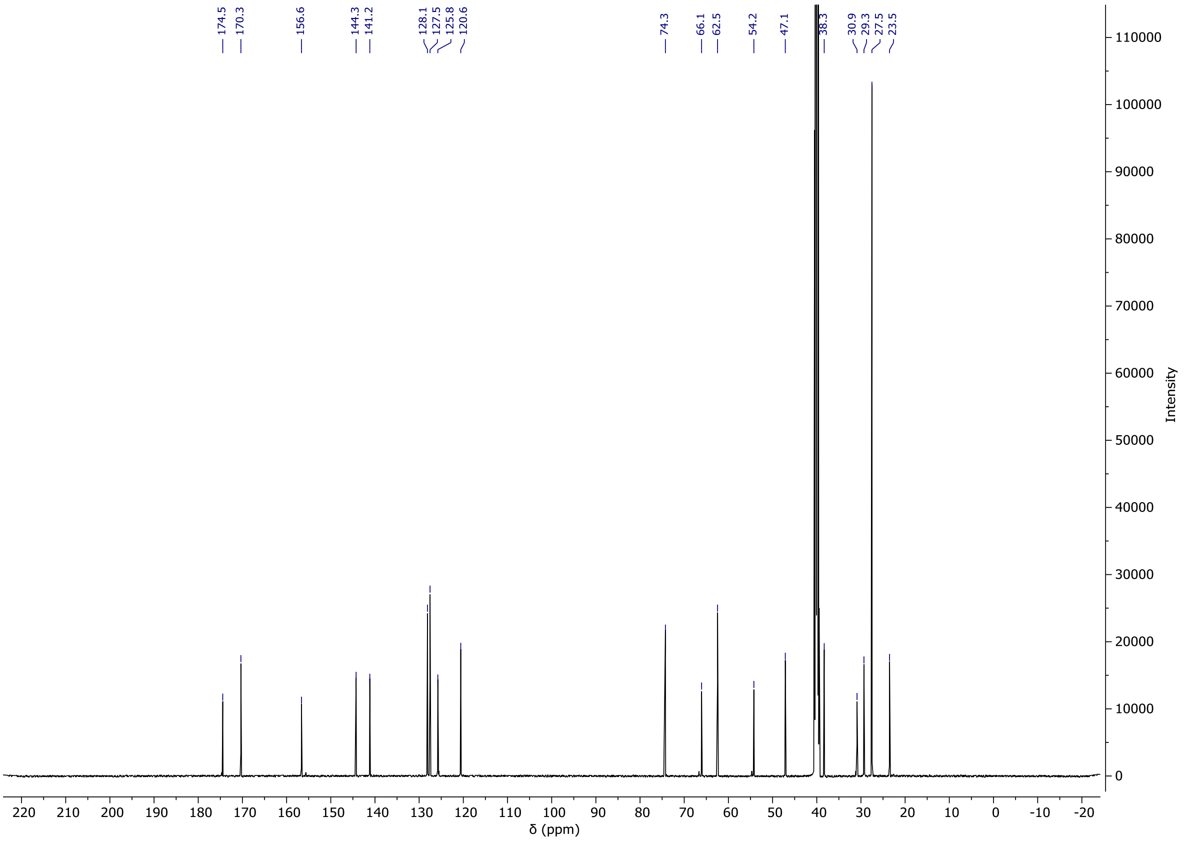

Supplementary Fig. 18 | ^{13}C-NMR spectrum of Fmoc-Lys(acO^{t}Bu)-OH.

5 Overview of peptides used in this work

Supplementary Table 6 | Peptides used were analysed by MALDI-TOF and LC-MS.

			Exact mass (Da)											
			Calculated					MALDI-TOF		LC-MS				
		Chemical formula	$[M+H]^{+}$	$[M+H]^{3+}$	$[M+H]^{4+}$	$[M+H]^{5+}$	$[M+H]^{6+}$	$[M+H]^{+}$	Error (ppm)	$[M+H]^{3+}$	$[M+H]^{4+}$	$[M+H]^{5+}$	$[M+H]^{6+}$	Analytical HPLC (%)
1	H3(1-21)K9me1	C95H175N37O27	2267.3536	756.5	567.6	454.3	378.7	2267.3570	1.48	756.4	567.7	454.4	378.8	>99%
2	H3(1-21)K9me2	C96H177N37O27	2281.3693	761.1	571.1	457.1	381.1	2281.3503	8.32	761.1	571.1	457.2	381.2	>88%
3	H3(1-21)K9me3	C97H179N37O27	2295.3849	765.8	574.6	459.9	383.4	2295.3686	7.12	765.8	574.7	460.0	383.5	>62%
4	H3(1-21)K9ac	C96H175N37O28	2295.3486	765.8	574.6	459.9	383.4	2295.3749	11.48	765.8	574.7	459.9	383.5	>93%
5	H3(1-21)K14me2	C96H177N37O27	2281.3693	761.1	571.1	457.1	381.1	2281.3964	11.88	761.3	571.2	457.2	381.1	>99%
6	H3(1-21)K14ac	C96H175N37O28	2295.3486	765.8	574.6	459.9	383.4	2295.3524	1.67	765.8	574.6	460.0	383.5	>87%
7	H3(1-21)K4me3K9me2	C99H183N37O27	2323.4162	775.1	581.6	465.5	388.1	2323.4216	2.31	775.2	581.9	465.5	388.2	>71%
8	H3(1-21)K4me3K9ac	C99H181N37O28	2337.3955	779.8	585.1	468.3	390.4	2337.3976	0.90	779.8	585.2	468.4	390.5	>94%
9	H3(1-21)K4me3	C97H179N37O27	2295.3849	765.8	574.6	459.9	383.4	2295.3906	2.46	765.8	574.6	460.0	383.6	>95%
10	H3(1-21)K4ac	C96H175N37O28	2295.3486	765.8	574.6	459.9	383.4	2295.3556	3.07	765.9	574.7	459.9	383.5	>92%
11	H3(1-21)K9acK14ac	C98H177N37O29	2337.3591	779.8	585.1	468.3	390.4	2337.3631	1.70	779.8	585.2	468.4	390.5	>95%
12	H3(1-15)K9ac	C65H120N25O22	1602.9040	535.0	401.5	321.4	268.0	1602.9248	12.99	535.0	401.5	321.4	N.D.	>96%
13	H3(1-21)K9ac-^{2}[H]$_{3}$	C96H172^{2}H3N37O28	2298.3668	766.8	575.3	460.5	383.9	2298.3834	7.22	766.7	575.3	460.5	384.0	>99%
14	H3(1-21)D-K9ac	C96H175N37O28	2295.3486	765.8	574.6	459.9	383.4	2295.3506	0.89	765.8	574.7	459.9	383.5	>93%
15	H3(1-21)K9acOH	C96H175N37O29	2311.3435	771.1	578.6	463.1	386.1	2311.3542	4.64	771.1	578.6	463.1	386.1	>99%
16	H3(1-44)K9ac	C204H357N75O55	2355.8644	786.0	589.7	472.0	393.5	2355.863*	0.58	N.D.	1178.4	943	786.1	>98%
27	H3(1-21)	C94H173N37O27	2253.3380	751.8	564.1	451.5	376.4	2253.3460	3.55	751.8	564.1	451.6	376.5	>95%
24	HIF1α(522-542)K532ac	C117H179N25O37S	2559.2693	853.8	640.6	512.7	427.4	2559.2802	4.27	854.0	640.6	N.D.	N.D.	>98%
25	HIF1α(664-684)K674ac	C94H160N34O33	2294.1965	765.4	574.3	459.6	383.2	2294.1621	15.01	765.5	574.4	459.8	N.D.	>93%
26	HIF1α(699-719)K709ac	C106H181N31O36	2433.3466	811.8	609.1	487.5	406.4	2433.3275	7.83	811.9	609.3	N.D.	N.D.	>98%

6 Analytical HPLC UV traces of peptides

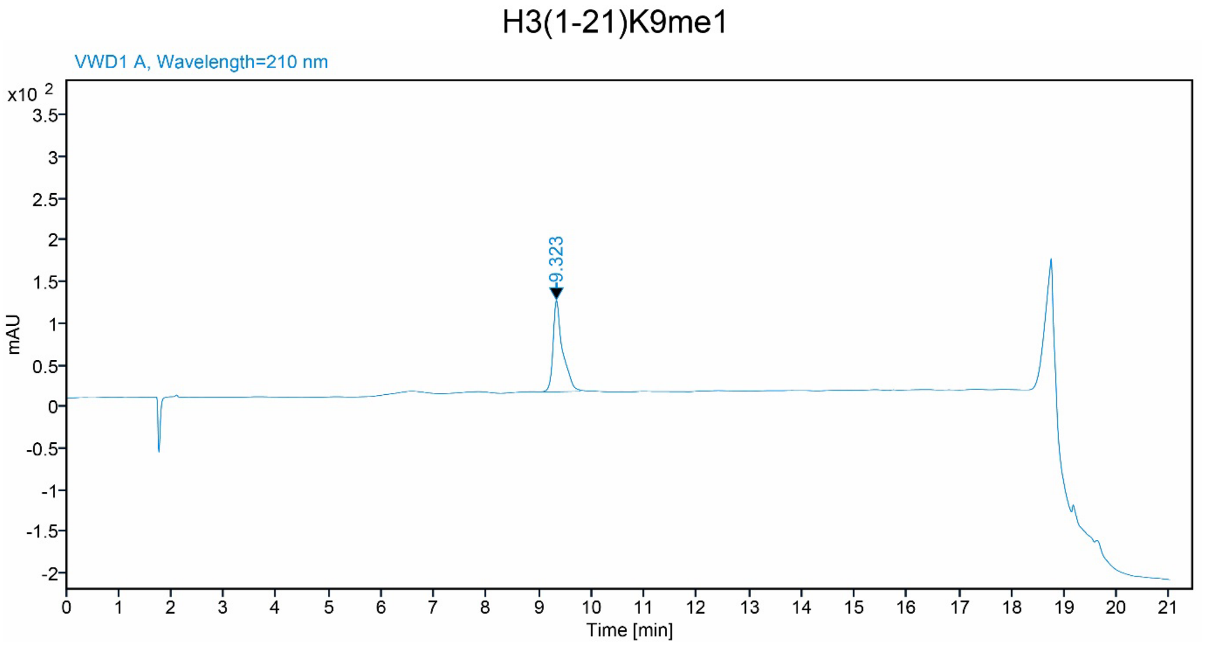

Supplementary Fig. 19a | Analytical HPLC UV spectrum of H3(1-21)K9me1.

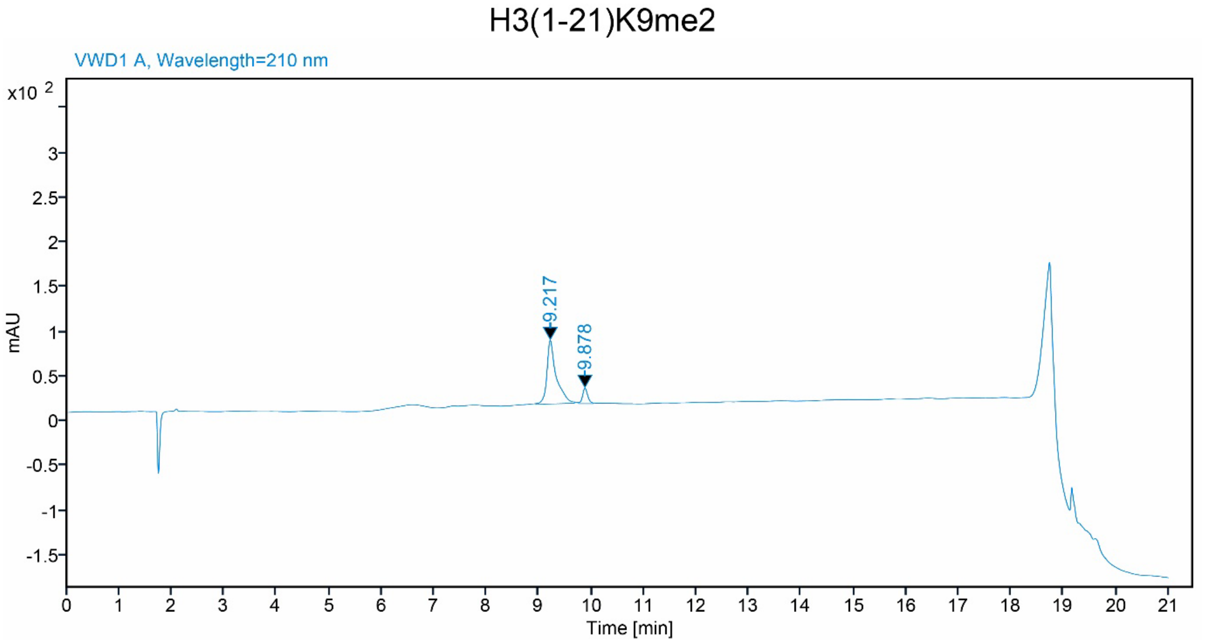

Supplementary Fig. 19b | Analytical HPLC UV spectrum of H3(1-21)K9me2.

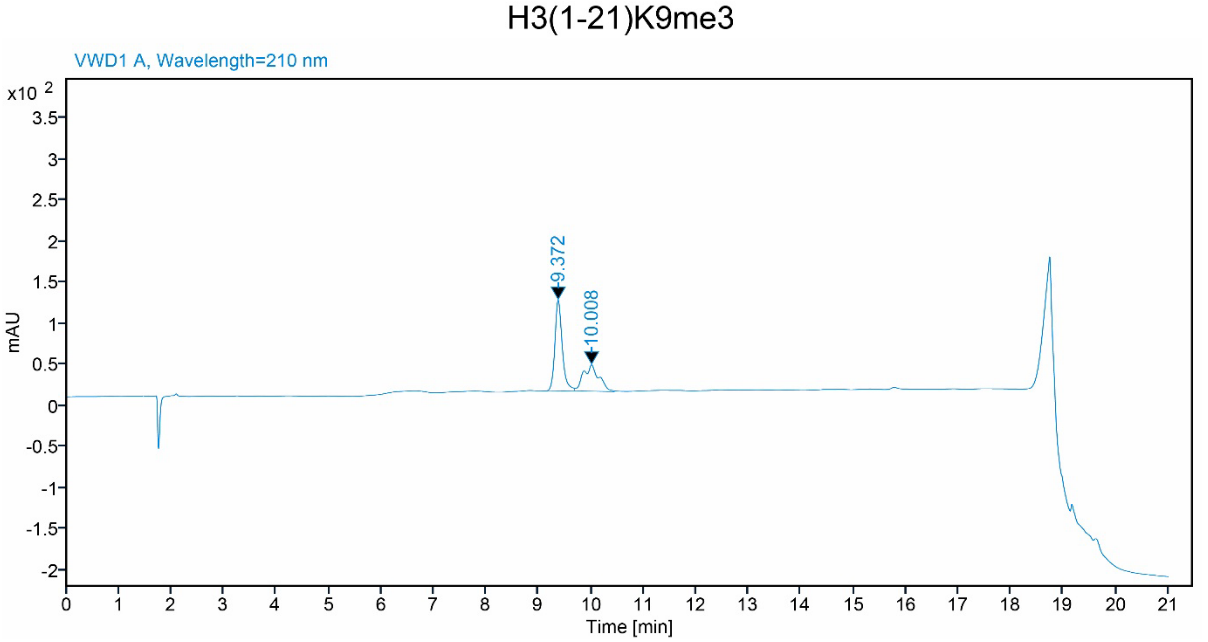

Supplementary Fig. 19c | Analytical HPLC UV spectrum of H3(1-21)K9me3.

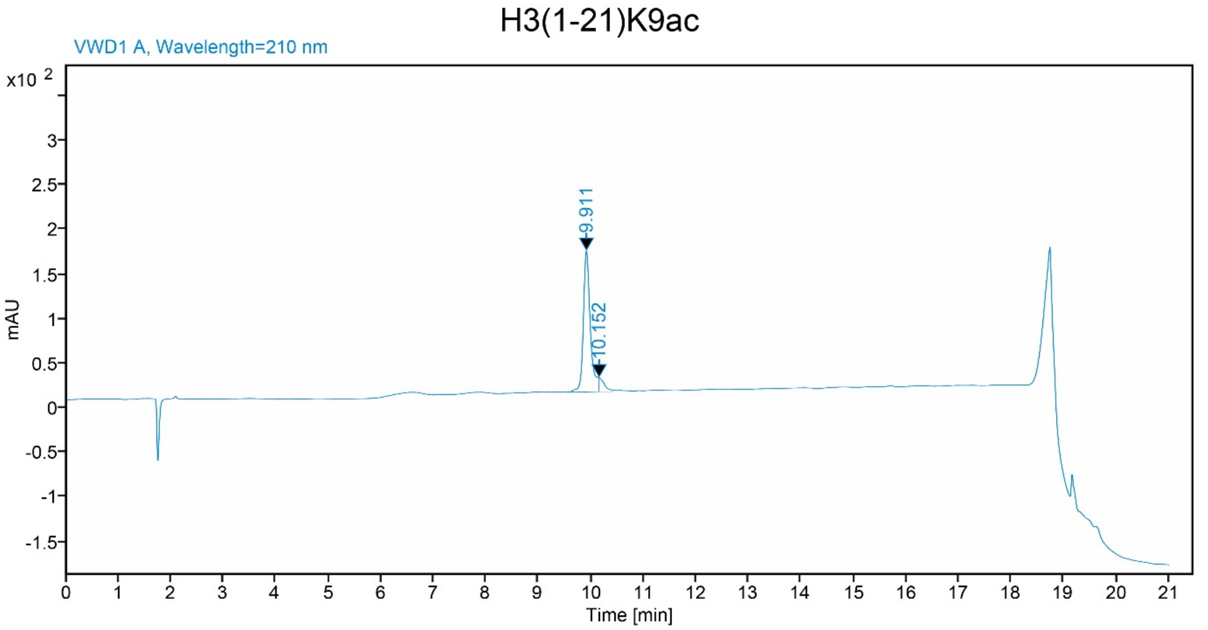

Supplementary Fig. 19d | Analytical HPLC UV spectrum of H3(1-21)K9ac.

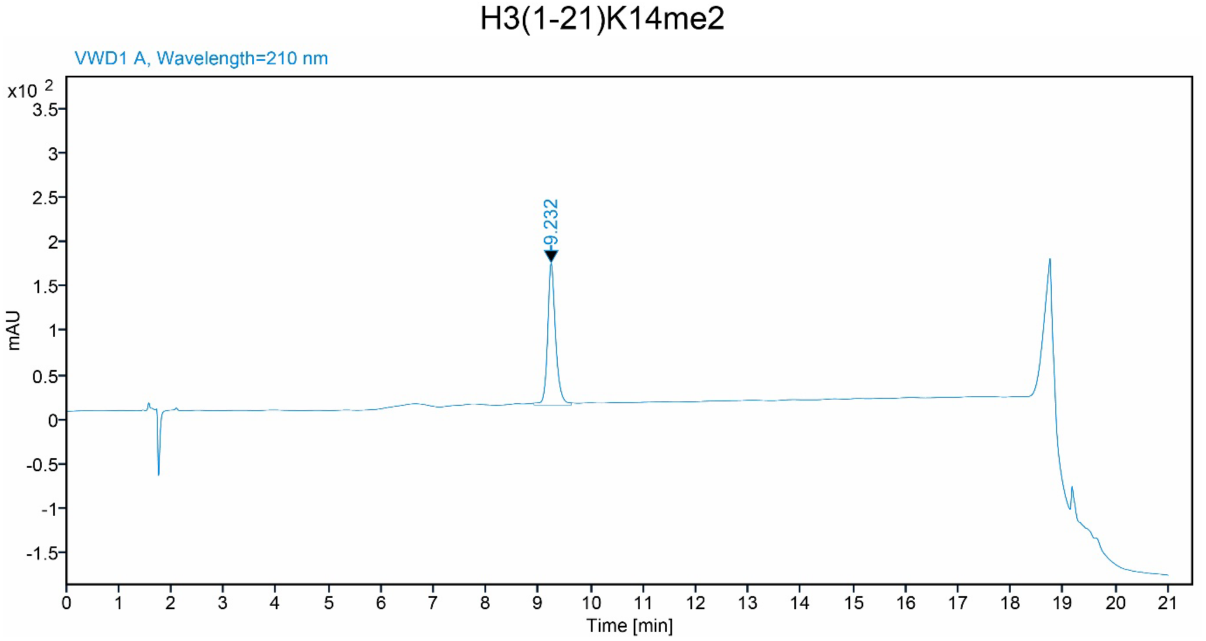

Supplementary Fig. 19e | Analytical HPLC UV spectrum of H3(1-21)K14me2.

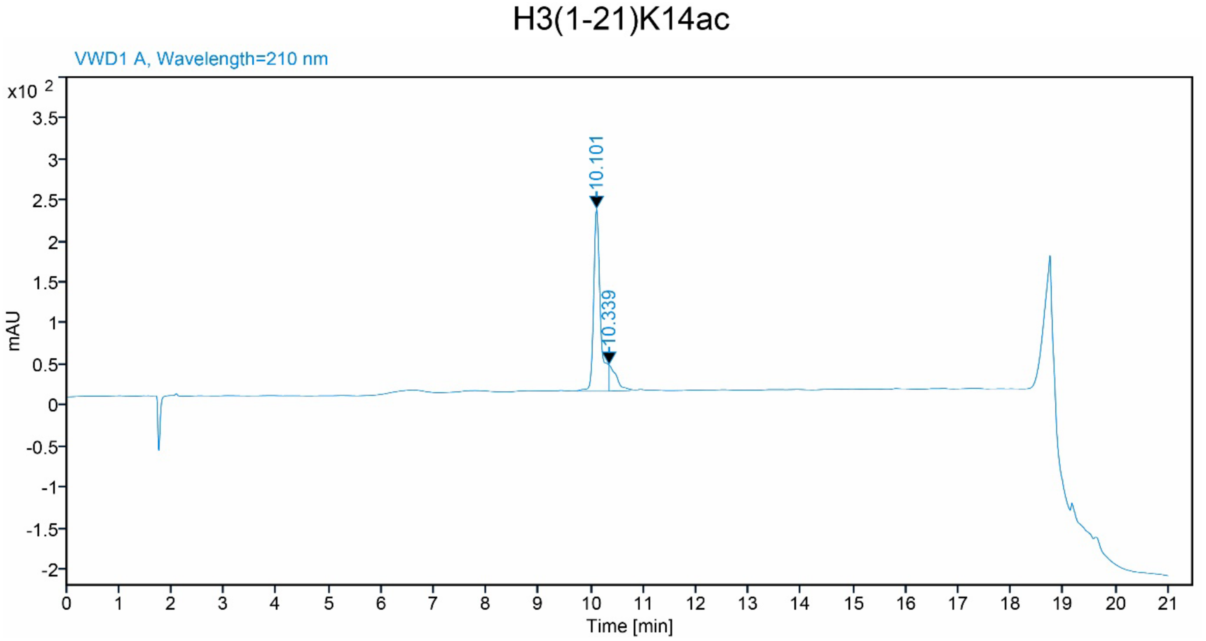

Supplementary Fig. 19f | Analytical HPLC UV spectrum of H3(1-21)K14ac.

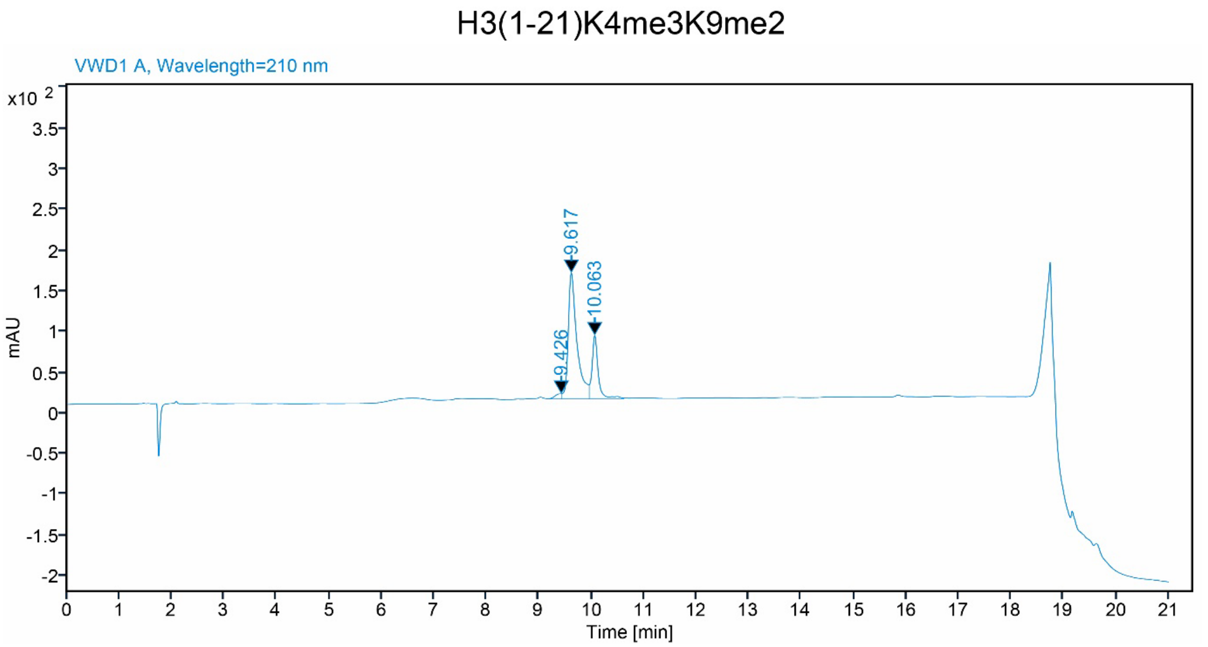

Supplementary Fig. 19g | Analytical HPLC UV spectrum of H3(1-21)K4me3K9me2.

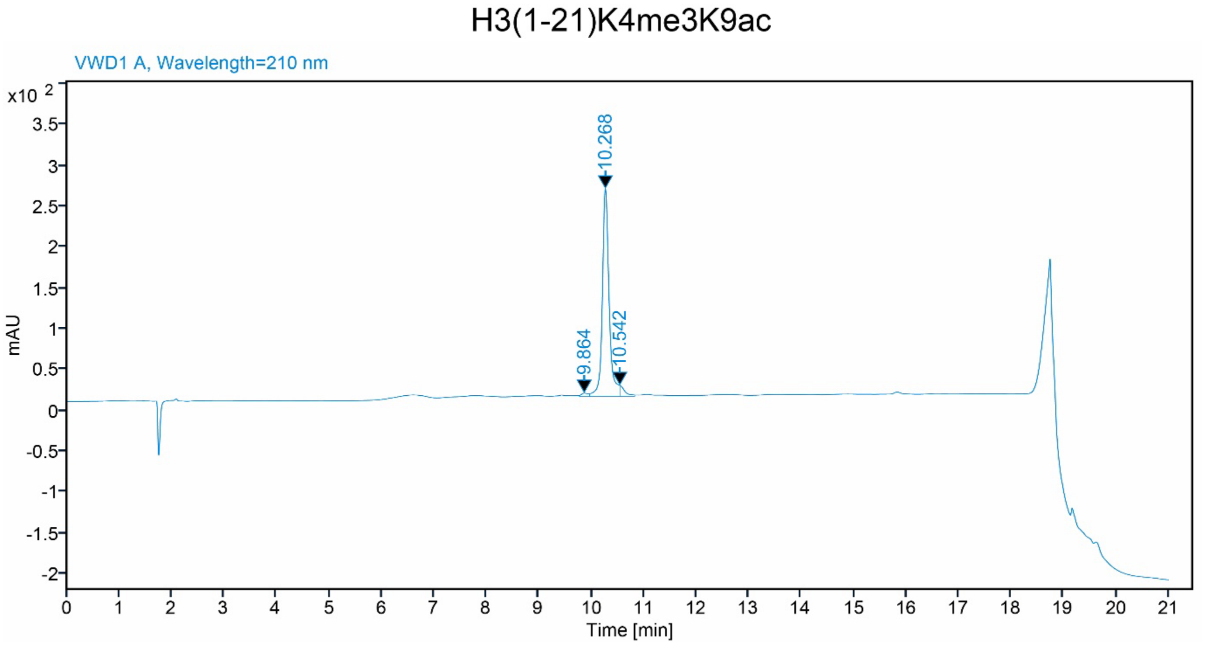

Supplementary Fig. 19h | Analytical HPLC UV spectrum of H3(1-21)K4me3K9ac.

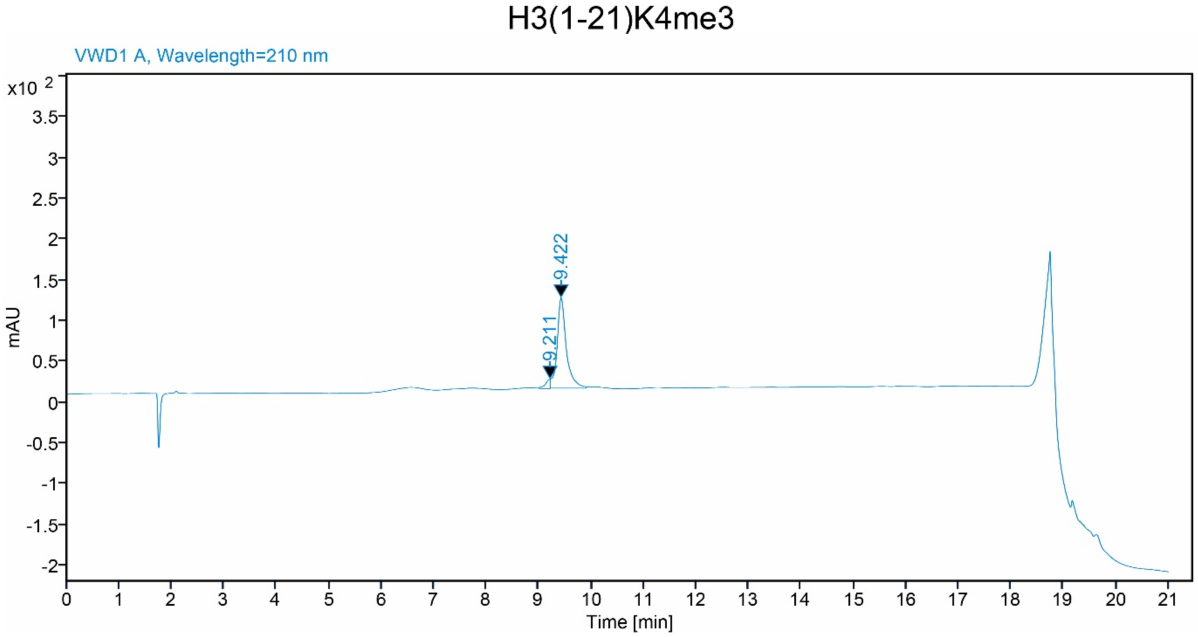

Supplementary Fig. 19i | Analytical HPLC UV spectrum of H3(1-21)K4me3.

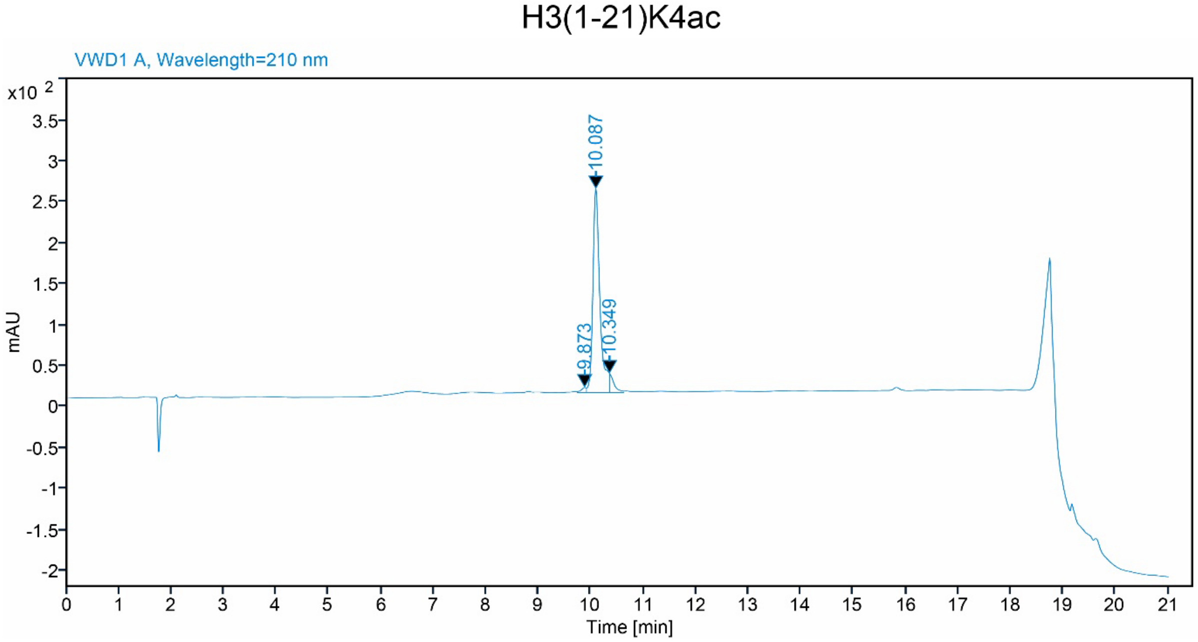

Supplementary Fig. 19j | Analytical HPLC UV spectrum of H3(1-21)K4ac.

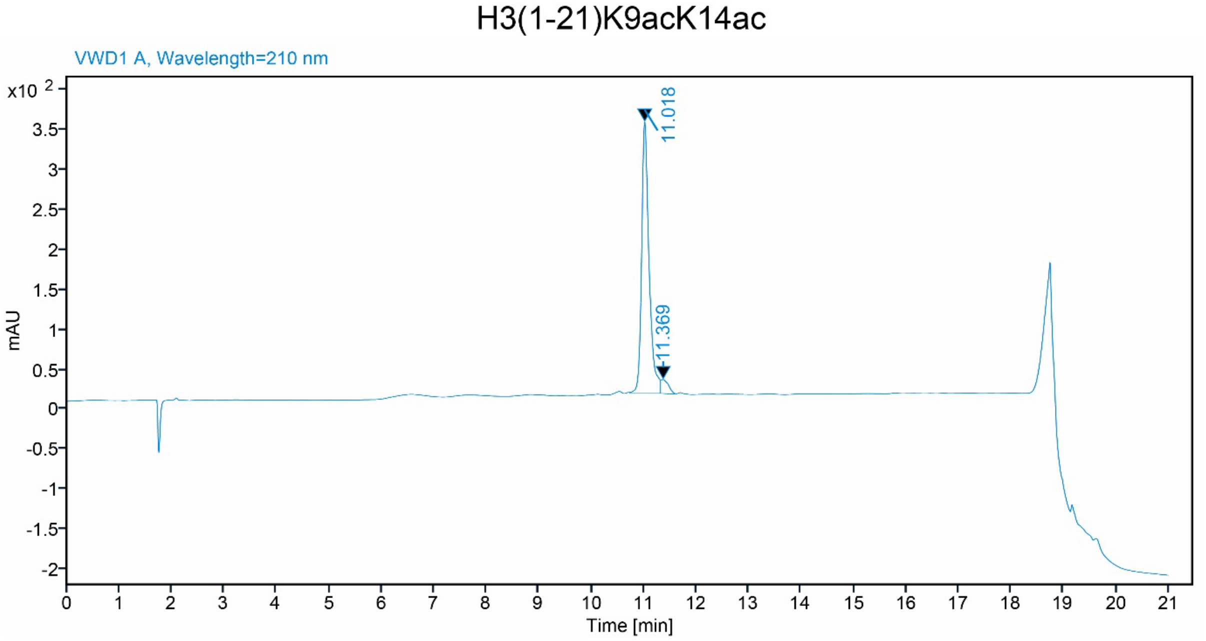

Supplementary Fig. 19k | Analytical HPLC UV spectrum of H3(1-21)K9acK14ac.

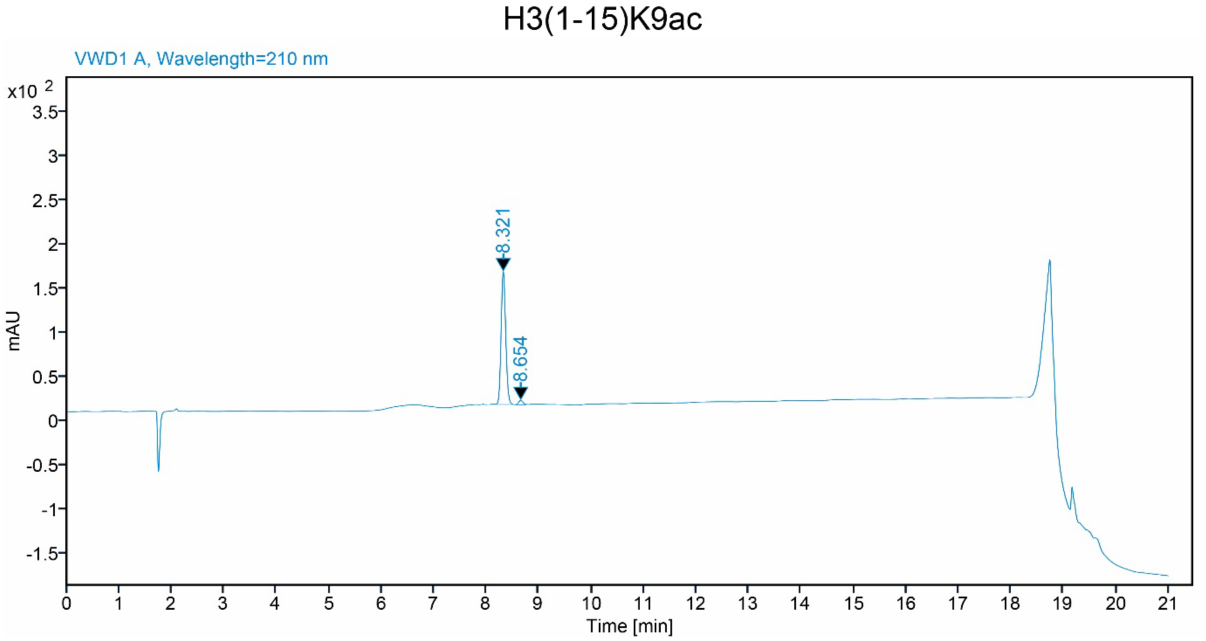

Supplementary Fig. 19l | Analytical HPLC UV spectrum of H3(1-15)K9ac.

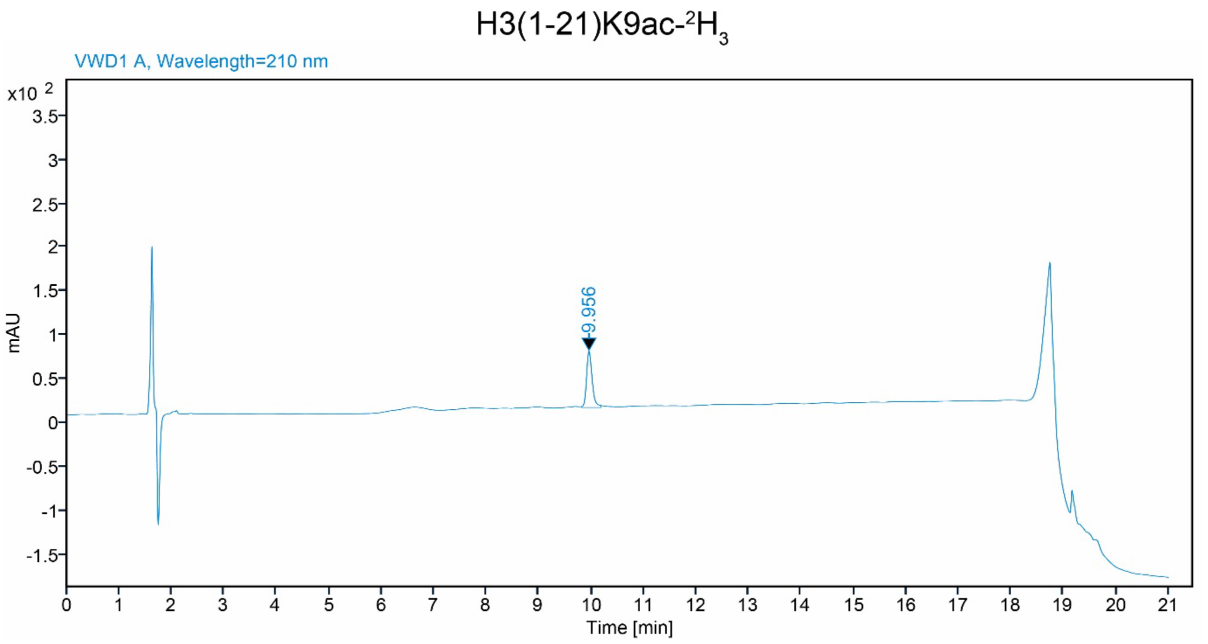

Supplementary Fig. 19m | Analytical HPLC UV spectrum of H3(1-21)K9ac-$^2H_3$.

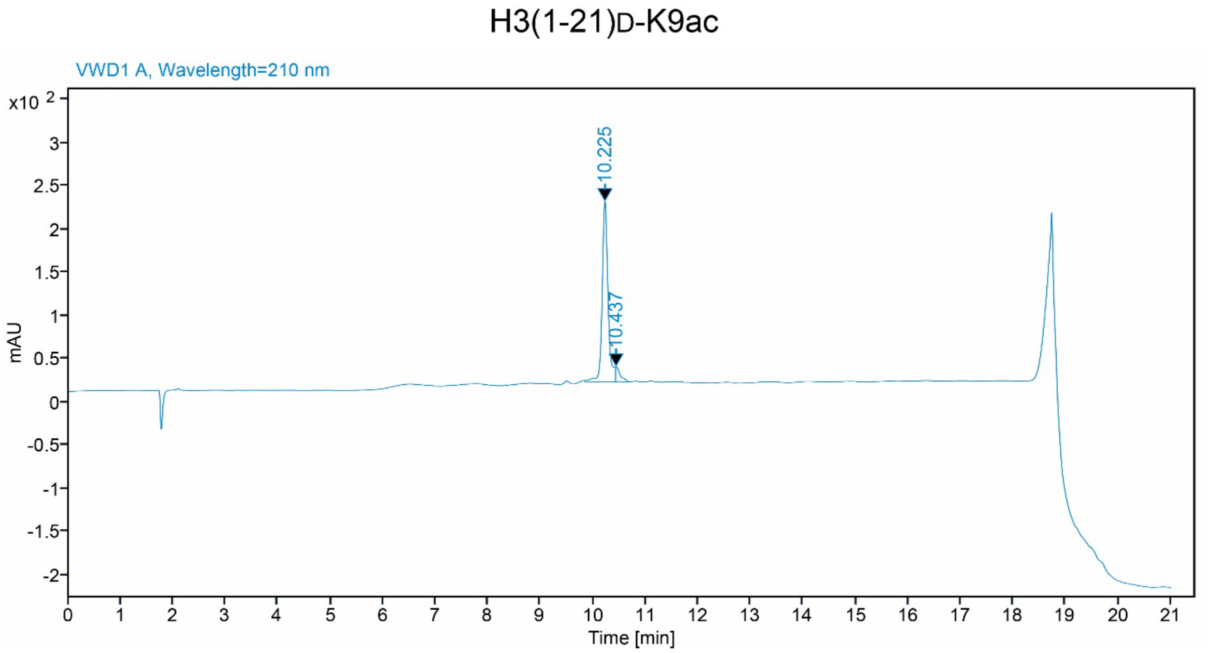

Supplementary Fig. 19o | Analytical HPLC UV spectrum of H3(1-21)D-K9ac.

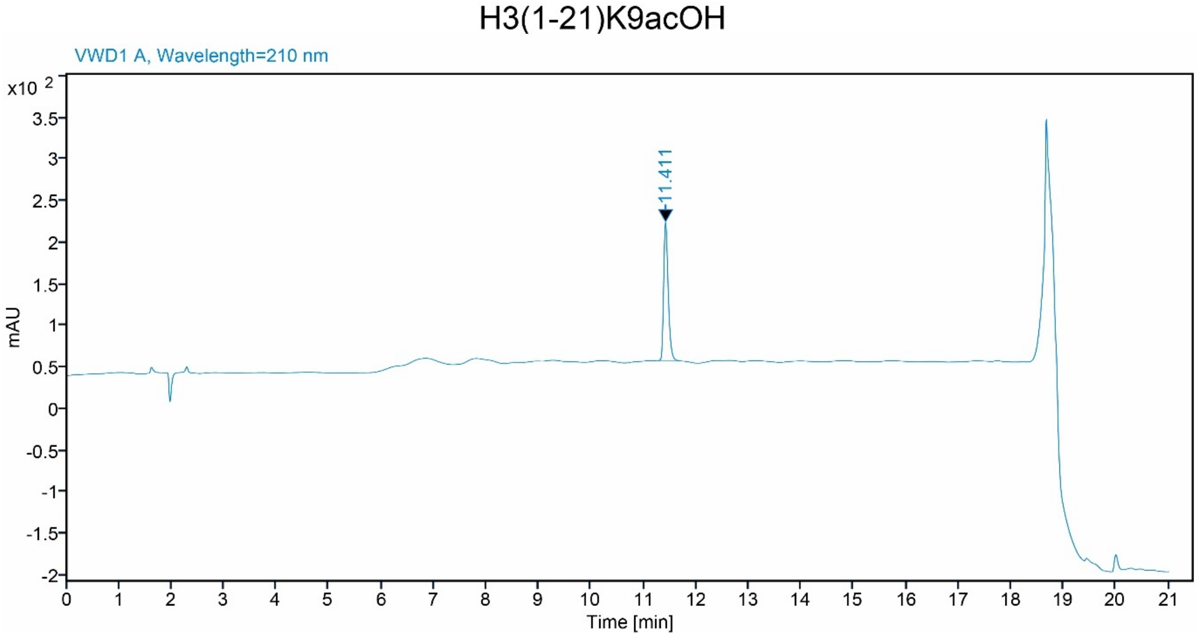

Supplementary Fig. 19p | Analytical HPLC UV spectrum of H3(1-21)K9acOH.

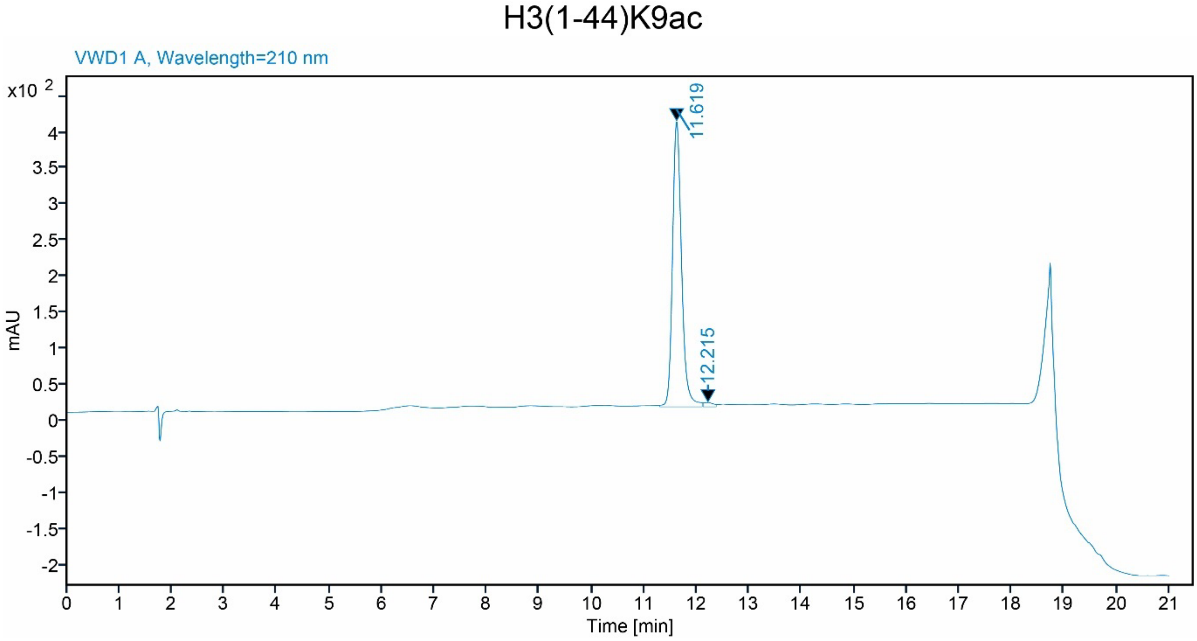

Supplementary Fig. 19q| Analytical HPLC UV spectrum of H3.2(1-44)K9ac.

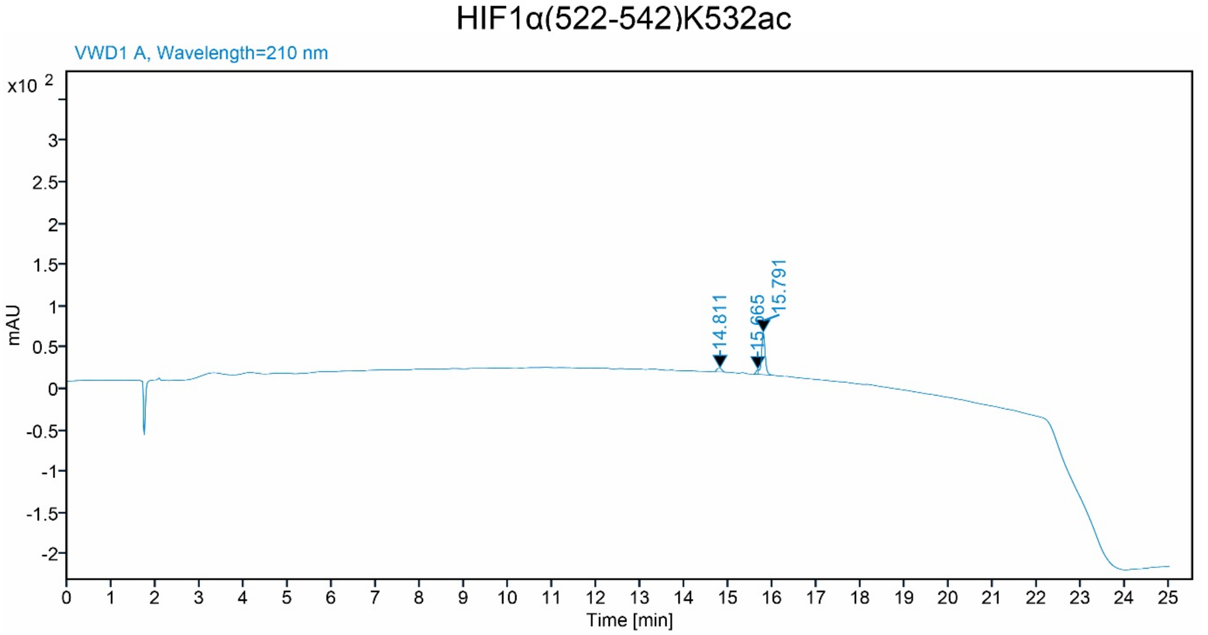

Supplementary Fig. 19r | Analytical HPLC UV spectrum of HIF1α(522-542)K532ac.

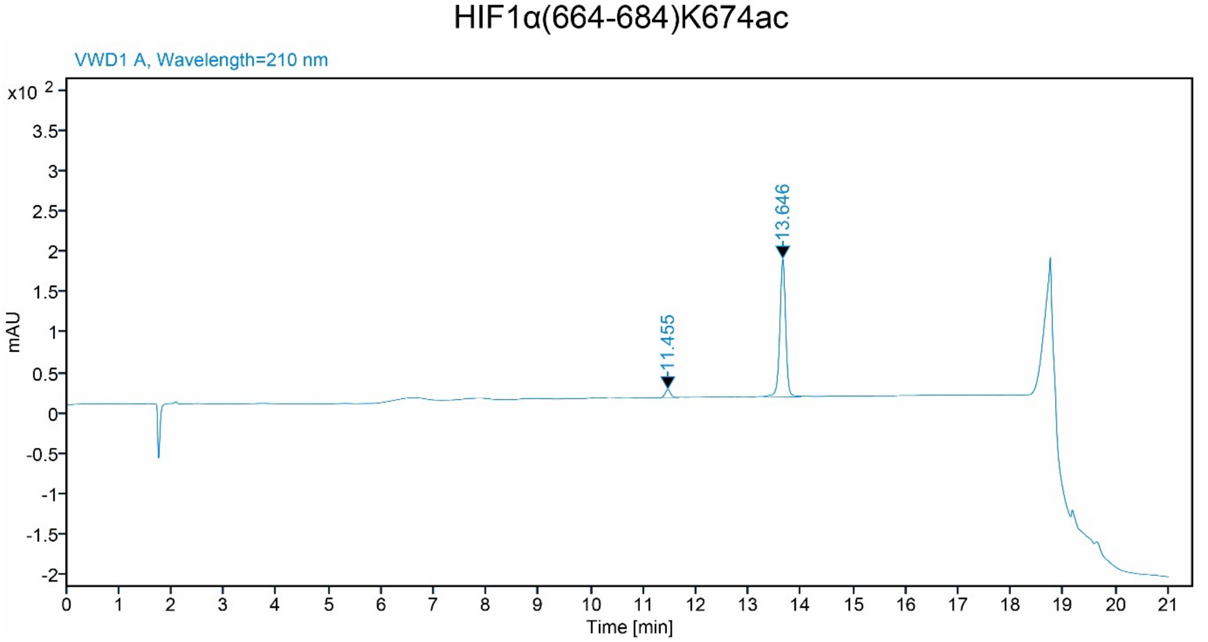

Supplementary Fig. 19s | Analytical HPLC UV spectrum of HIF1α(664-684)K674ac.

HIF1α(699-719)K709ac

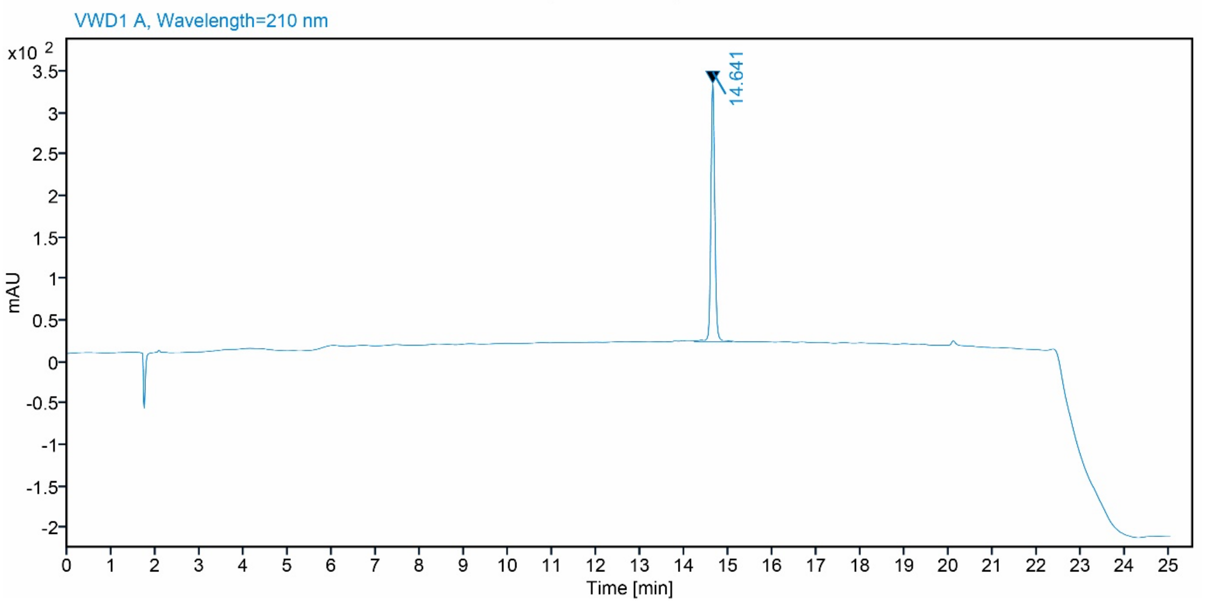

Supplementary Fig. 19t | Analytical HPLC UV spectrum of HIF1α(699-719)K709ac.

H3(1-21)

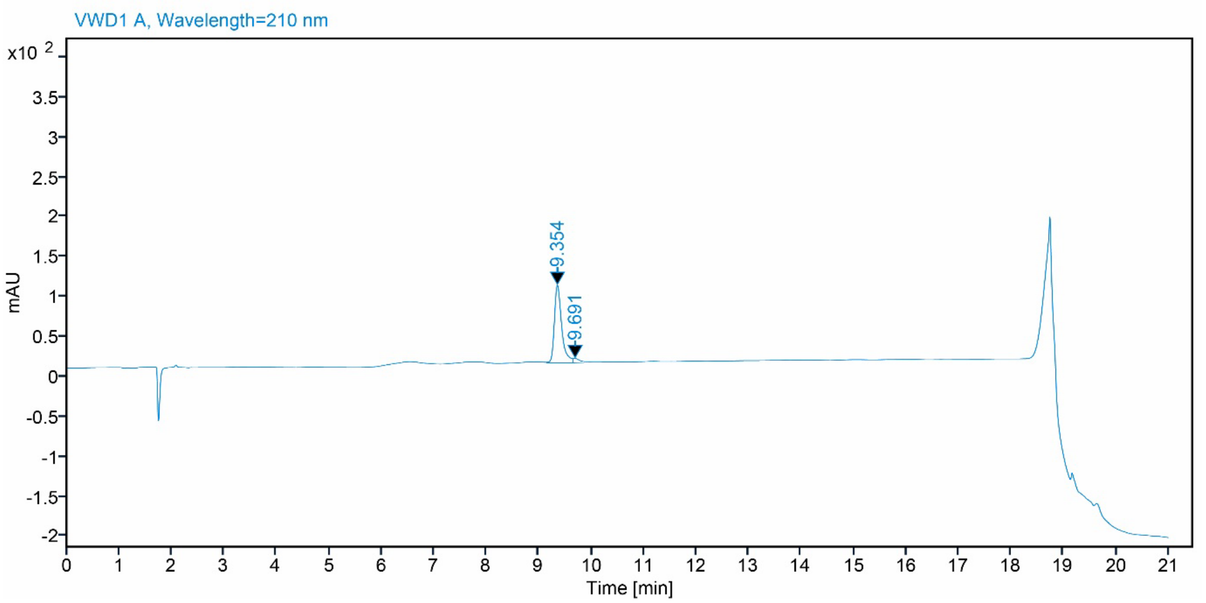

Supplementary Fig. 19u | Analytical HPLC UV spectrum of H3(1-21).

7 Uncropped western blot images

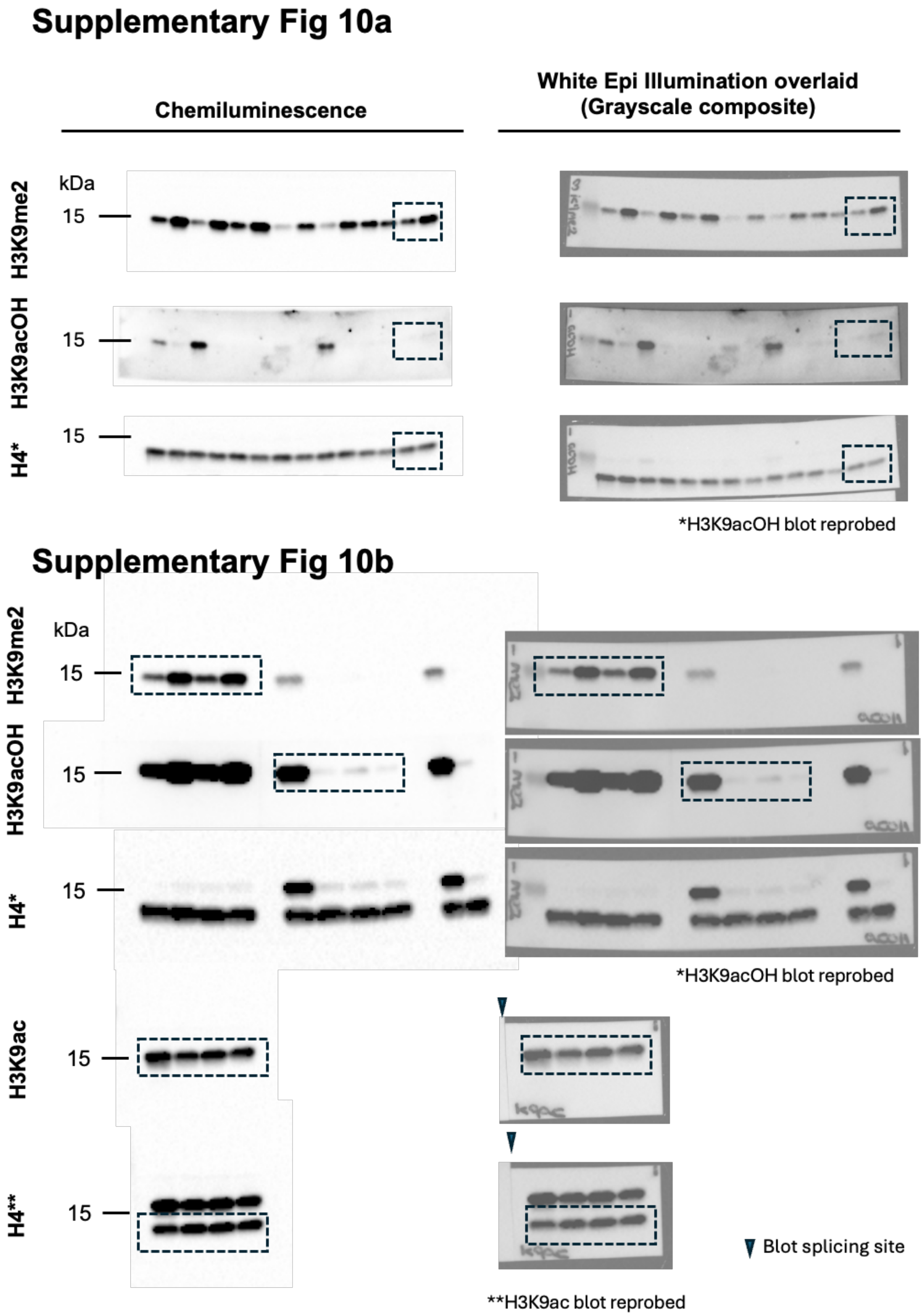

Supplementary Fig 10c

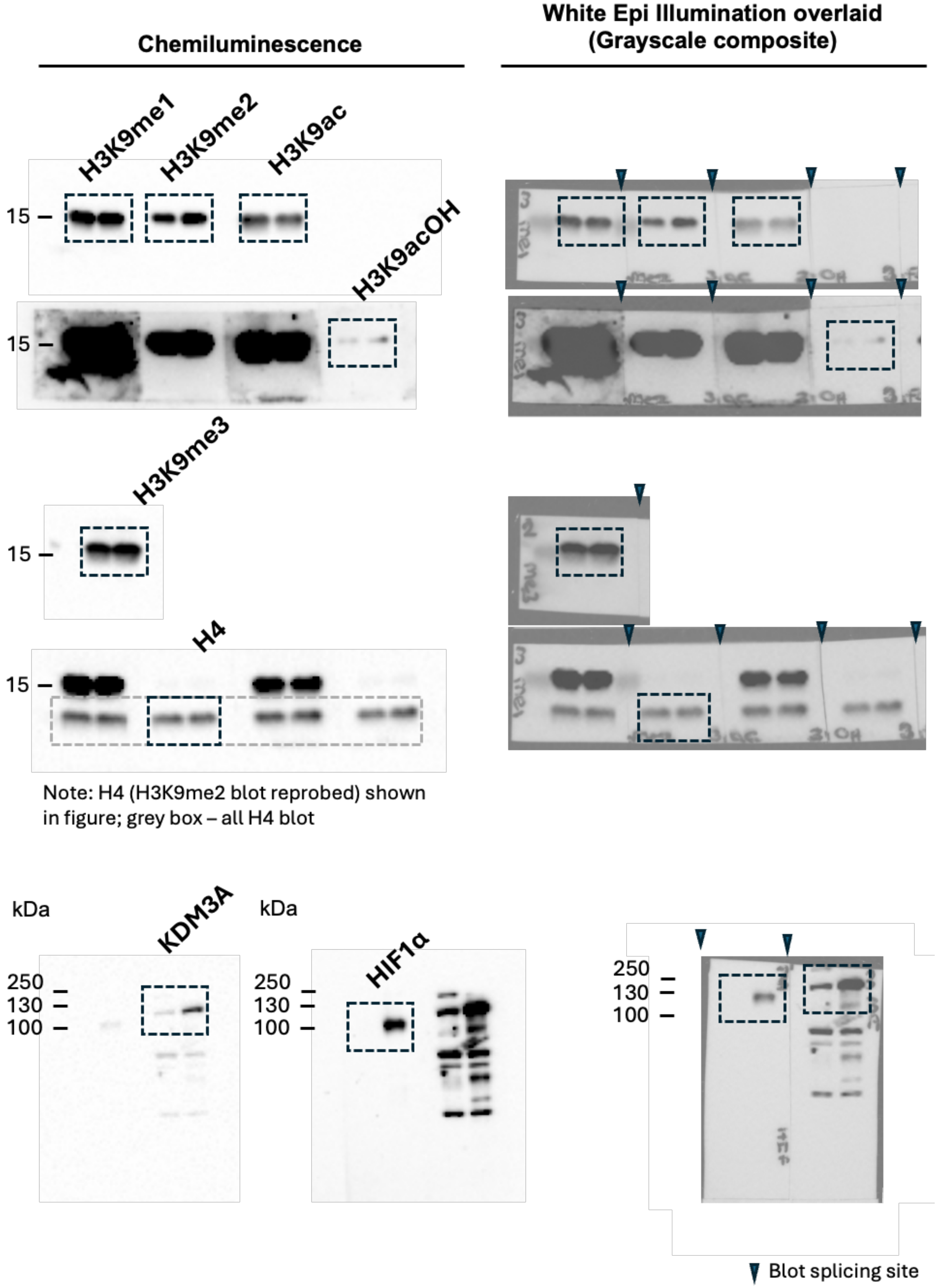

8 References

1 Rose, N. R. *et al.* Plant growth regulator daminozide is a selective inhibitor of human KDM2/7 histone demethylases. *J Med Chem* **55**, 6639-6643 (2012).

2 Ng, S. S. *et al.* Crystal structures of histone demethylase JMJD2A reveal basis for substrate specificity. *Nature* **448**, 87-91 (2007).

3 Hillringhaus, L. *et al.* Structural and Evolutionary Basis for the Dual Substrate Selectivity of Human KDM4 Histone Demethylase Family. *Journal of Biological Chemistry* **286**, 41616-41625 (2011).

4 Rose, N. R. *et al.* Inhibitor scaffolds for 2-oxoglutarate-dependent histone lysine demethylases. *J Med Chem* **51**, 7053-7056 (2008).

5 Johansson, C. *et al.* Structural analysis of human KDM5B guides histone demethylase inhibitor development. *Nat Chem Biol* **12**, 539-545 (2016).

6 Hallows, W. C., Lee, S. & Denu, J. M. Sirtuins deacetylate and activate mammalian acetyl-CoA synthetases. *Proc Natl Acad Sci U S A* **103**, 10230-10235 (2006).

7 Altamore, T. *et al.* Random-Coil:α-Helix Equilibria as a Reporter for the LewisX-LewisX Interaction. *Angewandte Chemie (International ed. in English)* **50**, 11167-11171 (2011).

8 Nikolovska-Coleska, Z. *et al.* Design and characterization of bivalent Smac-based peptides as antagonists of XIAP and development and validation of a fluorescence polarization assay for XIAP containing both BIR2 and BIR3 domains. *Anal Biochem* **374**, 87-98 (2008).
