# Supplementary material for: KDM3A catalyses the oxidation of acetyl-lysine to hydroxyacetyl-lysine on histone H3K9
Source: Nat Chem. 2026 Apr 15;18(5):823–34. doi: 10.1038/s41557-026-02112-x (PMC13149330; doi:10.1038/s41557-026-02112-x)
Supplement: Supplementary file 2 — Reporting Summary [file 41557_2026_2112_MOESM2_ESM.pdf]

Corresponding author(s): *Double-blind peer review submissions: write DBPR and your manuscript number here instead of author names.*

Last updated by author(s): Jan 10, 2025

# Reporting Summary

Nature Portfolio wishes to improve the reproducibility of the work that we publish. This form provides structure for consistency and transparency in reporting. For further information on Nature Portfolio policies, see our Editorial Policies and the Editorial Policy Checklist.

## Statistics

For all statistical analyses, confirm that the following items are present in the figure legend, table legend, main text, or Methods section.

| n/a | Confirmed | |
|---|---|---|
| ☐ | ☒ | The exact sample size (*n*) for each experimental group/condition, given as a discrete number and unit of measurement |
| ☐ | ☒ | A statement on whether measurements were taken from distinct samples or whether the same sample was measured repeatedly |
| ☐ | ☒ | The statistical test(s) used AND whether they are one- or two-sided<br>*Only common tests should be described solely by name; describe more complex techniques in the Methods section.* |
| ☒ | ☐ | A description of all covariates tested |
| ☒ | ☐ | A description of any assumptions or corrections, such as tests of normality and adjustment for multiple comparisons |
| ☐ | ☒ | A full description of the statistical parameters including central tendency (e.g. means) or other basic estimates (e.g. regression coefficient) AND variation (e.g. standard deviation) or associated estimates of uncertainty (e.g. confidence intervals) |
| ☒ | ☐ | For null hypothesis testing, the test statistic (e.g. *F*, *t*, *r*) with confidence intervals, effect sizes, degrees of freedom and *P* value noted<br>*Give P values as exact values whenever suitable.* |
| ☒ | ☐ | For Bayesian analysis, information on the choice of priors and Markov chain Monte Carlo settings |
| ☒ | ☐ | For hierarchical and complex designs, identification of the appropriate level for tests and full reporting of outcomes |
| ☒ | ☐ | Estimates of effect sizes (e.g. Cohen's *d*, Pearson's *r*), indicating how they were calculated |

*Our web collection on statistics for biologists contains articles on many of the points above.*

## Software and code

Policy information about availability of computer code

| | |
|---|---|
| Data collection | NMR - Bruker 500MHz; Low-res MS - Agilent Infinity II 1260 UPLC + MSD XT; High-res MS - Water Aquity UPLC + Xevo G2-XS; Peptide analysis and enzyme assays - MALDI-TOF Bruker rapiflex or MicroFlex LRF; Agilent 1290 Infinity II LC system connected to Agilent 6550; Orbitrap Fusion Lumos (Thermo Scientific)BMG PHERAstar FSX; Western blot / dot-blot - Biorad ChemiDoc MP Imaging System; Operetta High Content Imaging System; Illumina NextSeq 500 |
| Data analysis | BMG Labtech MARS (v 3.32); Microsoft Excel; GraphPad Prism (v 9.5.0); Harmony 3.5 software (Perkin Elmer); BWA (0.7.5a-r405); SAMtool view (0.1.19); Bedtools (2.17.0); flexAnalysis (v 3.4, build:79); Agilent MassHunter Qualitativve Analysis (vversion B.07.00); EpiProfile 2.0; Skyliner software |

For manuscripts utilizing custom algorithms or software that are central to the research but not yet described in published literature, software must be made available to editors and reviewers. We strongly encourage code deposition in a community repository (e.g. GitHub). See the Nature Portfolio guidelines for submitting code & software for further information.

# Data

Policy information about availability of data

All manuscripts must include a data availability statement. This statement should provide the following information, where applicable:

- Accession codes, unique identifiers, or web links for publicly available datasets
- A description of any restrictions on data availability
- For clinical datasets or third party data, please ensure that the statement adheres to our policy

Mass spectrometry proteomic data of histone samples are made available through Proteomics IDEntification database (PRIDE, accession number: PXD057969). ChIP-Sequencing data has been deposited in the NCBI Gene Expression Omnibus (GEO) data base (accession number: GSE282321).

# Human research participants

Policy information about studies involving human research participants and Sex and Gender in Research.

| | |
|---|---|
| Reporting on sex and gender | *Use the terms sex (biological attribute) and gender (shaped by social and cultural circumstances) carefully in order to avoid confusing both terms. Indicate if findings apply to only one sex or gender; describe whether sex and gender were considered in study design whether sex and/or gender was determined based on self-reporting or assigned and methods used. Provide in the source data disaggregated sex and gender data where this information has been collected, and consent has been obtained for sharing of individual-level data; provide overall numbers in this Reporting Summary. Please state if this information has not been collected. Report sex- and gender-based analyses where performed, justify reasons for lack of sex- and gender-based analysis.* |
| Population characteristics | *Describe the covariate-relevant population characteristics of the human research participants (e.g. age, genotypic information, past and current diagnosis and treatment categories). If you filled out the behavioural & social sciences study design questions and have nothing to add here, write "See above."* |
| Recruitment | *Describe how participants were recruited. Outline any potential self-selection bias or other biases that may be present and how these are likely to impact results.* |
| Ethics oversight | *Identify the organization(s) that approved the study protocol.* |

Note that full information on the approval of the study protocol must also be provided in the manuscript.

# Field-specific reporting

Please select the one below that is the best fit for your research. If you are not sure, read the appropriate sections before making your selection.

☐ Life sciences ☐ Behavioural & social sciences ☐ Ecological, evolutionary & environmental sciences

For a reference copy of the document with all sections, see nature.com/documents/nr-reporting-summary-flat.pdf

# Life sciences study design

All studies must disclose on these points even when the disclosure is negative.

| | |
|---|---|
| Sample size | All biochemical and cellular data were performed in n=2-4 (noted in Figure caption). |
| Data exclusions | No data were excluded. |
| Replication | All assays were performed in n=1-4. Mean and standard deviations are provided where appropriate, indicating reproducibility. |
| Randomization | N/A |
| Blinding | N/A |

# Behavioural & social sciences study design

All studies must disclose on these points even when the disclosure is negative.

| | |
|---|---|
| Study description | *Briefly describe the study type including whether data are quantitative, qualitative, or mixed-methods (e.g. qualitative cross-sectional, quantitative experimental, mixed-methods case study).* |

| | |
|---|---|
| Research sample | *State the research sample (e.g. Harvard university undergraduates, villagers in rural India) and provide relevant demographic information (e.g. age, sex) and indicate whether the sample is representative. Provide a rationale for the study sample chosen. For studies involving existing datasets, please describe the dataset and source.* |
| Sampling strategy | *Describe the sampling procedure (e.g. random, snowball, stratified, convenience). Describe the statistical methods that were used to predetermine sample size OR if no sample-size calculation was performed, describe how sample sizes were chosen and provide a rationale for why these sample sizes are sufficient. For qualitative data, please indicate whether data saturation was considered, and what criteria were used to decide that no further sampling was needed.* |
| Data collection | *Provide details about the data collection procedure, including the instruments or devices used to record the data (e.g. pen and paper, computer, eye tracker, video or audio equipment) whether anyone was present besides the participant(s) and the researcher, and whether the researcher was blind to experimental condition and/or the study hypothesis during data collection.* |
| Timing | *Indicate the start and stop dates of data collection. If there is a gap between collection periods, state the dates for each sample cohort.* |
| Data exclusions | *If no data were excluded from the analyses, state so OR if data were excluded, provide the exact number of exclusions and the rationale behind them, indicating whether exclusion criteria were pre-established.* |
| Non-participation | *State how many participants dropped out/declined participation and the reason(s) given OR provide response rate OR state that no participants dropped out/declined participation.* |
| Randomization | *If participants were not allocated into experimental groups, state so OR describe how participants were allocated to groups, and if allocation was not random, describe how covariates were controlled.* |

# Ecological, evolutionary & environmental sciences study design

All studies must disclose on these points even when the disclosure is negative.

| | |
|---|---|
| Study description | *Briefly describe the study. For quantitative data include treatment factors and interactions, design structure (e.g. factorial, nested, hierarchical), nature and number of experimental units and replicates.* |
| Research sample | *Describe the research sample (e.g. a group of tagged Passer domesticus, all Stenocereus thurberi within Organ Pipe Cactus National Monument), and provide a rationale for the sample choice. When relevant, describe the organism taxa, source, sex, age range and any manipulations. State what population the sample is meant to represent when applicable. For studies involving existing datasets, describe the data and its source.* |
| Sampling strategy | *Note the sampling procedure. Describe the statistical methods that were used to predetermine sample size OR if no sample-size calculation was performed, describe how sample sizes were chosen and provide a rationale for why these sample sizes are sufficient.* |
| Data collection | *Describe the data collection procedure, including who recorded the data and how.* |
| Timing and spatial scale | *Indicate the start and stop dates of data collection, noting the frequency and periodicity of sampling and providing a rationale for these choices. If there is a gap between collection periods, state the dates for each sample cohort. Specify the spatial scale from which the data are taken* |
| Data exclusions | *If no data were excluded from the analyses, state so OR if data were excluded, describe the exclusions and the rationale behind them, indicating whether exclusion criteria were pre-established.* |
| Reproducibility | *Describe the measures taken to verify the reproducibility of experimental findings. For each experiment, note whether any attempts to repeat the experiment failed OR state that all attempts to repeat the experiment were successful.* |
| Randomization | *Describe how samples/organisms/participants were allocated into groups. If allocation was not random, describe how covariates were controlled. If this is not relevant to your study, explain why.* |
| Blinding | *Describe the extent of blinding used during data acquisition and analysis. If blinding was not possible, describe why OR explain why blinding was not relevant to your study.* |

Did the study involve field work? ☐ Yes ☐ No

## Field work, collection and transport

| | |
|---|---|
| Field conditions | *Describe the study conditions for field work, providing relevant parameters (e.g. temperature, rainfall).* |
| Location | *State the location of the sampling or experiment, providing relevant parameters (e.g. latitude and longitude, elevation, water depth).* |
| Access & import/export | *Describe the efforts you have made to access habitats and to collect and import/export your samples in a responsible manner and in* |

| | |
|---|---|
| Access & import/export | *compliance with local, national and international laws, noting any permits that were obtained (give the name of the issuing authority, the date of issue, and any identifying information).* |
| Disturbance | *Describe any disturbance caused by the study and how it was minimized.* |

# Reporting for specific materials, systems and methods

We require information from authors about some types of materials, experimental systems and methods used in many studies. Here, indicate whether each material, system or method listed is relevant to your study. If you are not sure if a list item applies to your research, read the appropriate section before selecting a response.

## Materials & experimental systems

| n/a | Involved in the study |
|---|---|
| ☐ | ☒ Antibodies |
| ☐ | ☒ Eukaryotic cell lines |
| ☒ | ☐ Palaeontology and archaeology |
| ☒ | ☐ Animals and other organisms |
| ☒ | ☐ Clinical data |
| ☒ | ☐ Dual use research of concern |

## Methods

| n/a | Involved in the study |
|---|---|
| ☐ | ☒ ChIP-seq |
| ☒ | ☐ Flow cytometry |
| ☒ | ☐ MRI-based neuroimaging |

# Antibodies

| | |
|---|---|
| Antibodies used | actin (Sigma, A1978, Lot: 065M4837V), Flag (Sigma, F1804, Lot: SLBN5629V), Flag (Sigma, F7425, Lot: 085M4774V), HA (sc-7392, Lot: G1818), HA (CST, 3724S, Lot: 8), HIF-1α (BD Biosciences, 610959, Lot: 5296905), H3K9ac (Abcam, ab4441, GR3290365-1, GR3229436-1, GR3253211), H3K9acOH fraction: R63, P1, F1.3 & F1.4, R43, P1, and F1.4 in this study), H3K9me3 (Abcam, ab8898, Lot: GR3217826-1, GR3245584-1), H3K9me2 (Abcam, ab1220, Lot: GR325223-3, GR3228498-2, GR325223-4, GR3308902-5), H3K9me1 (EpiCypher, 13-0014, Lot: 14247001), H3K9me0 (Active Motif, 61399, Lot: 34612001), Histone H4 (Abcam, ab177840, Lot: GR3189348-8), KDM3A (Proteintech, 12835-1-AP, Lot: 00009716), KDM3B (CST, 2621S, Lot: 1), Vinculin (sigma, V9131, Lot: N/A), anti-Rabbit IgG (Vector, PI-1000-1) and anti-Mouse IgG (Vector, PI-2000-1). |
| Validation | See manufacturers website for data on commercial antibodies. Custom made antibody (H3K9acOH) for this study has been validated (see Supplementary Figure 16) |

# Eukaryotic cell lines

Policy information about cell lines and Sex and Gender in Research

| | |
|---|---|
| Cell line source(s) | HEK293T (CRL-3216), HeLa (CRM-CCL-2), U-2 OS (HTB-96) and ES-E14TG2a (CRL1821) were from ATCC; RPE-1, RPE-1(CrWT) and RPE-1(Cr22.1) and RPE1(Cr22.2) were kind gifts from Patricia Yeyeti |
| Authentication | None of the cells purchased / used were authenticated |
| Mycoplasma contamination | All cells tested negative for mycoplasma |
| Commonly misidentified lines (See ICLAC register) | *Name any commonly misidentified cell lines used in the study and provide a rationale for their use.* |

# ChIP-seq

## Data deposition

☒ Confirm that both raw and final processed data have been deposited in a public database such as GEO.

☒ Confirm that you have deposited or provided access to graph files (e.g. BED files) for the called peaks.

| | |
|---|---|
| Data access links<br>*May remain private before publication.* | Data access links<br><br>https://www.ncbi.nlm.nih.gov/geo/query/acc.cgi?acc=GSE282321<br>Enter token crqfeygwrvufnof |
| Files in database submission | 1_DMSO_K9ac_S1_R1_001.fastq.gz<br>3_DMSO_K9acOH_S3_R1_001.fastq.gz<br>7_DMSO_K4me3_S7_R1_001.fastq.gz<br>9_DMSO_H3_S9_R1_001.fastq.gz<br>11_DMSO_input_S11_R1_001.fastq.gz<br>processed data file<br>DMSO_K9ac_S1_DM_unmapped_HS_mapped_filtered_blklistrmd_sorted.bw |

DMSO_K9acOH_S3_DM_unmapped_HS_mapped_filtered_blklistrmd_sorted.bw
DMSO_K4me3_S7_DM_unmapped_HS_mapped_filtered_blklistrmd_sorted.bw
DMSO_H3_S9_DM_unmapped_HS_mapped_filtered_blklistrmd_sorted.bw
DMSO_input_S11_DM_unmapped_HS_mapped_filtered_blklistrmd_sorted.bw

Genome browser session (e.g. UCSC)

N/A

## Methodology

Replicates

Single replicates were performed for each condition

Sequencing depth

raw reads uniquely mapped reads read length
1_DMSO_K9ac_S1_R1_001.fastq.gz 50202614 4508782 75 single-end
3_DMSO_K9acOH_S3_R1_001.fastq.gz 62821726 5462923 75 single-end
7_DMSO_K4me3_S7_R1_001.fastq.gz 29306431 2613540 75 single-end
9_DMSO_H3_S9_R1_001.fastq.gz 38418491 3481054 75 single-end
11_DMSO_input_S11_R1_001.fastq.gz 24349981 2149720 75 single-end

Antibodies

H3K9acOH In house H3K9acOH
H3K9ac Abcam ab4441
H3K4me3 Cell Signaling 9751S
Total H3 Abcam ab1791

Peak calling parameters

N/A

Data quality

N/A

Software

BWA (0.7.5a-r405)
SAMtools (0.1.19)
Bedtools (2.17.0)
Ngs.plot.r (2.6.1)
