# Supplementary material for: KDM3A catalyses the oxidation of acetyl-lysine to hydroxyacetyl-lysine on histone H3K9
Source: Nat Chem. 2026 Apr 15;18(5):823–34. doi: 10.1038/s41557-026-02112-x (PMC13149330; doi:10.1038/s41557-026-02112-x)

Source Data Fig 2a

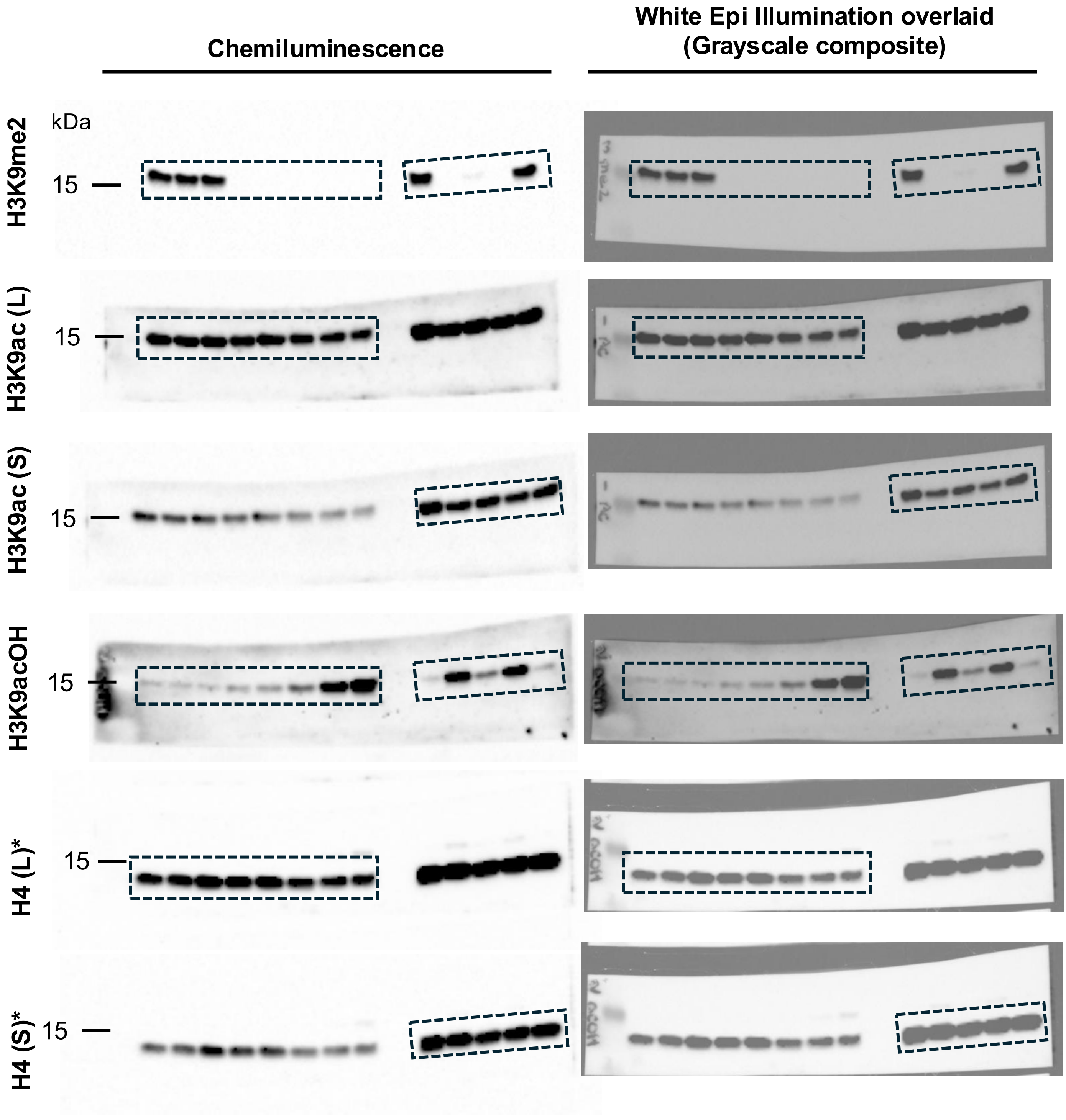

(L) : long exposure
(S) : short exposure
* : H3K9acOH blot reprobed

Source Data Fig 2b

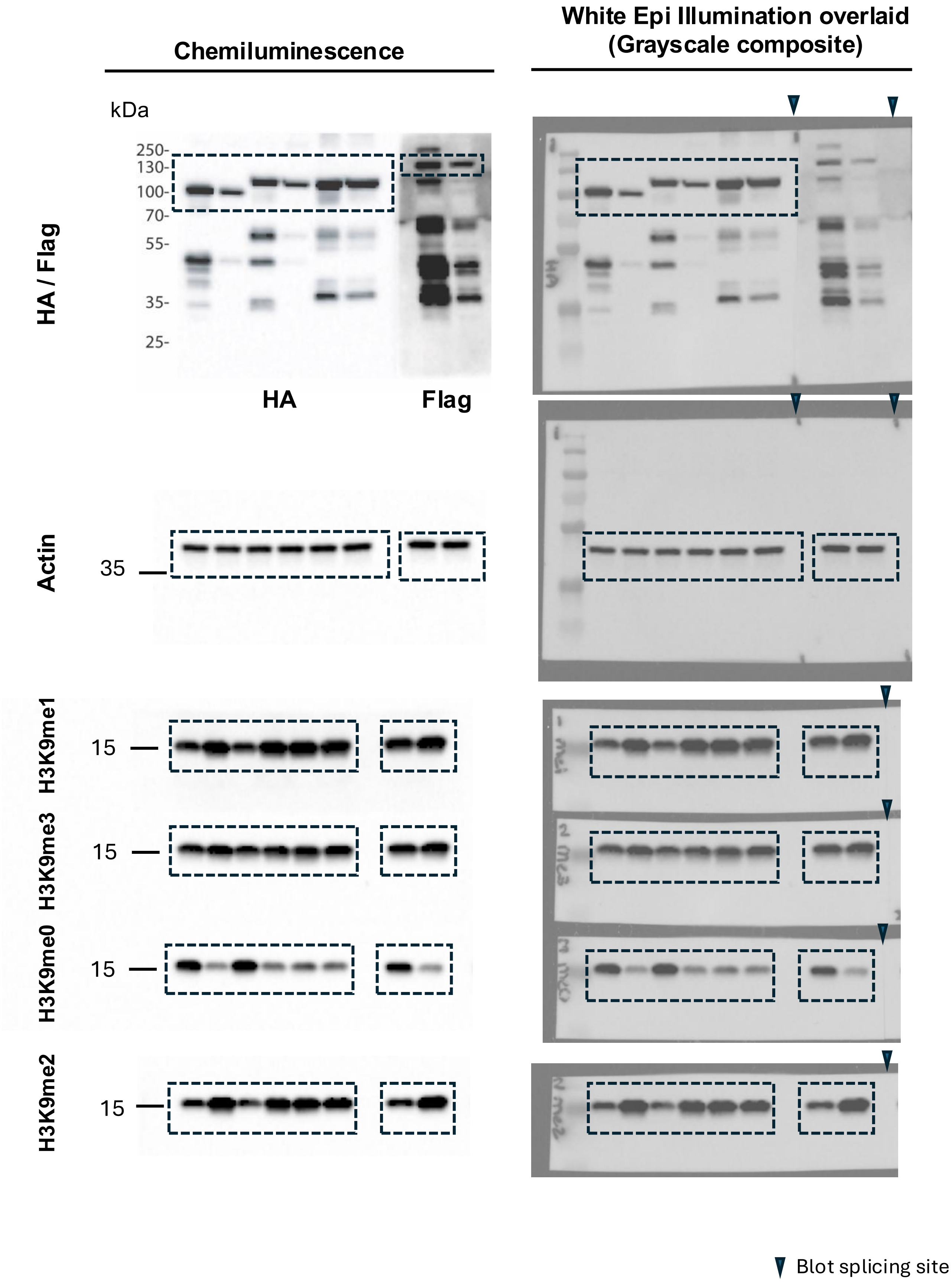

Source Data Fig 2b (continued)

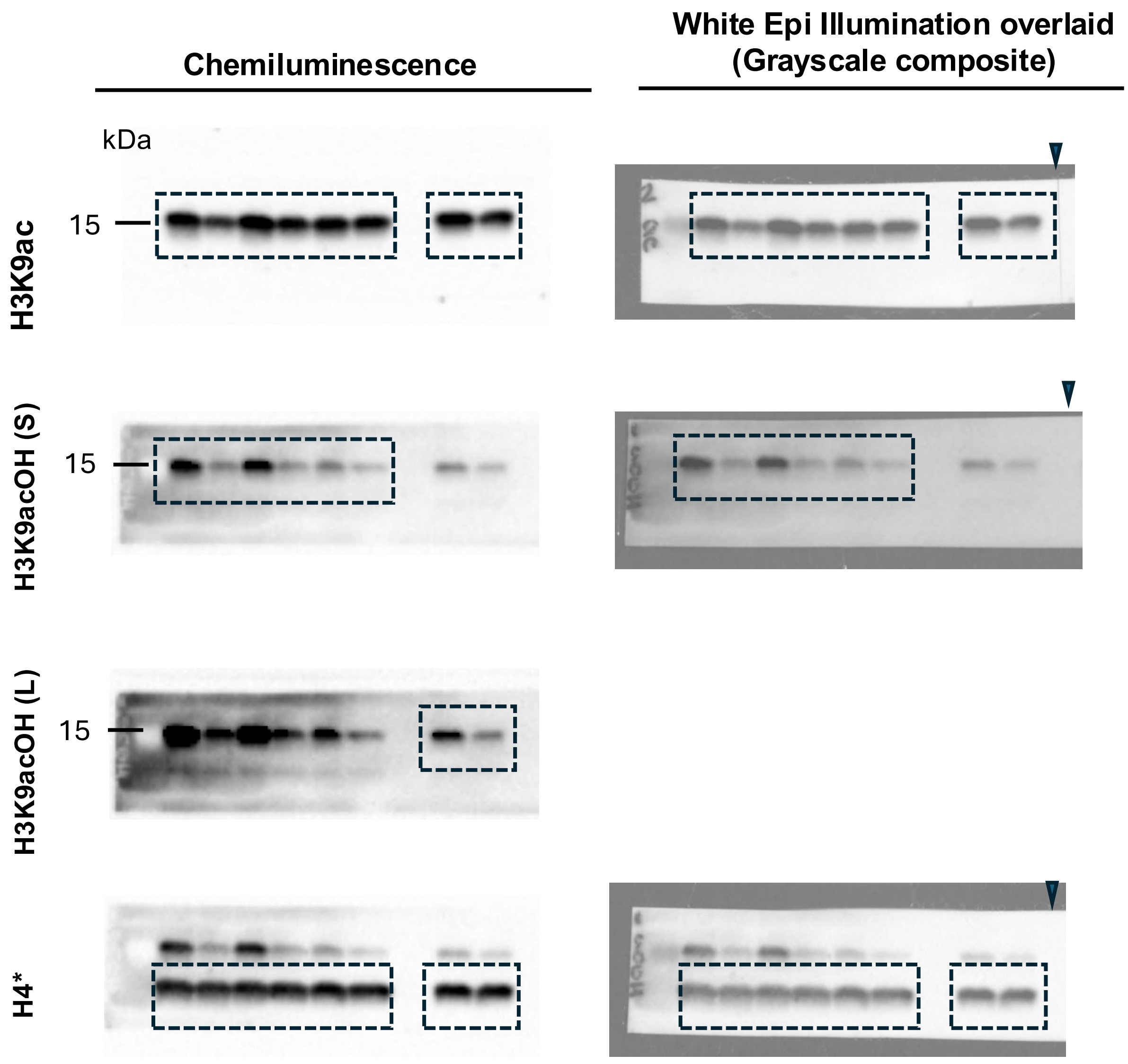

(L) : long exposure
(S) : short exposure
* : H3K9acOH blot reprobed

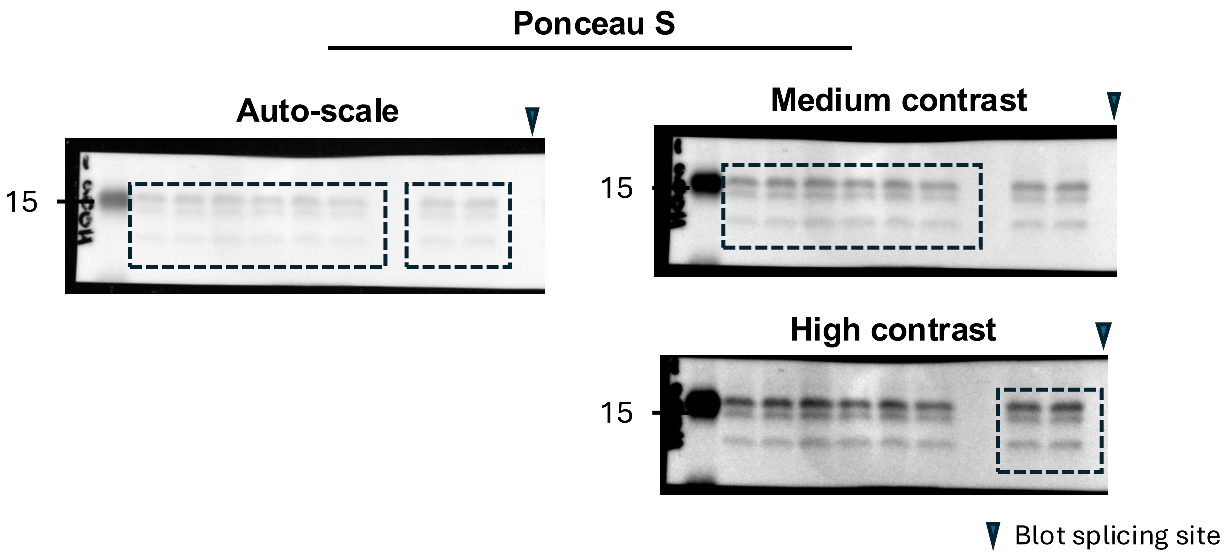

Supplement: Supplementary file 4 — Uncropped western gel blots for Fig. 2a,b and immunofluorescence data analysis for Fig. 2f. [file 41557_2026_2112_MOESM4_ESM.zip › SDF_Fig2/SDF Fig 2ab.pdf]
