# Supplementary material for: KDM3A catalyses the oxidation of acetyl-lysine to hydroxyacetyl-lysine on histone H3K9
Source: Nat Chem. 2026 Apr 15;18(5):823–34. doi: 10.1038/s41557-026-02112-x (PMC13149330; doi:10.1038/s41557-026-02112-x)
Supplement: Supplementary file 5 — Uncropped western gel blots for Fig. 3a,b,d. [file 41557_2026_2112_MOESM5_ESM.pdf]

Source Data Fig 3a

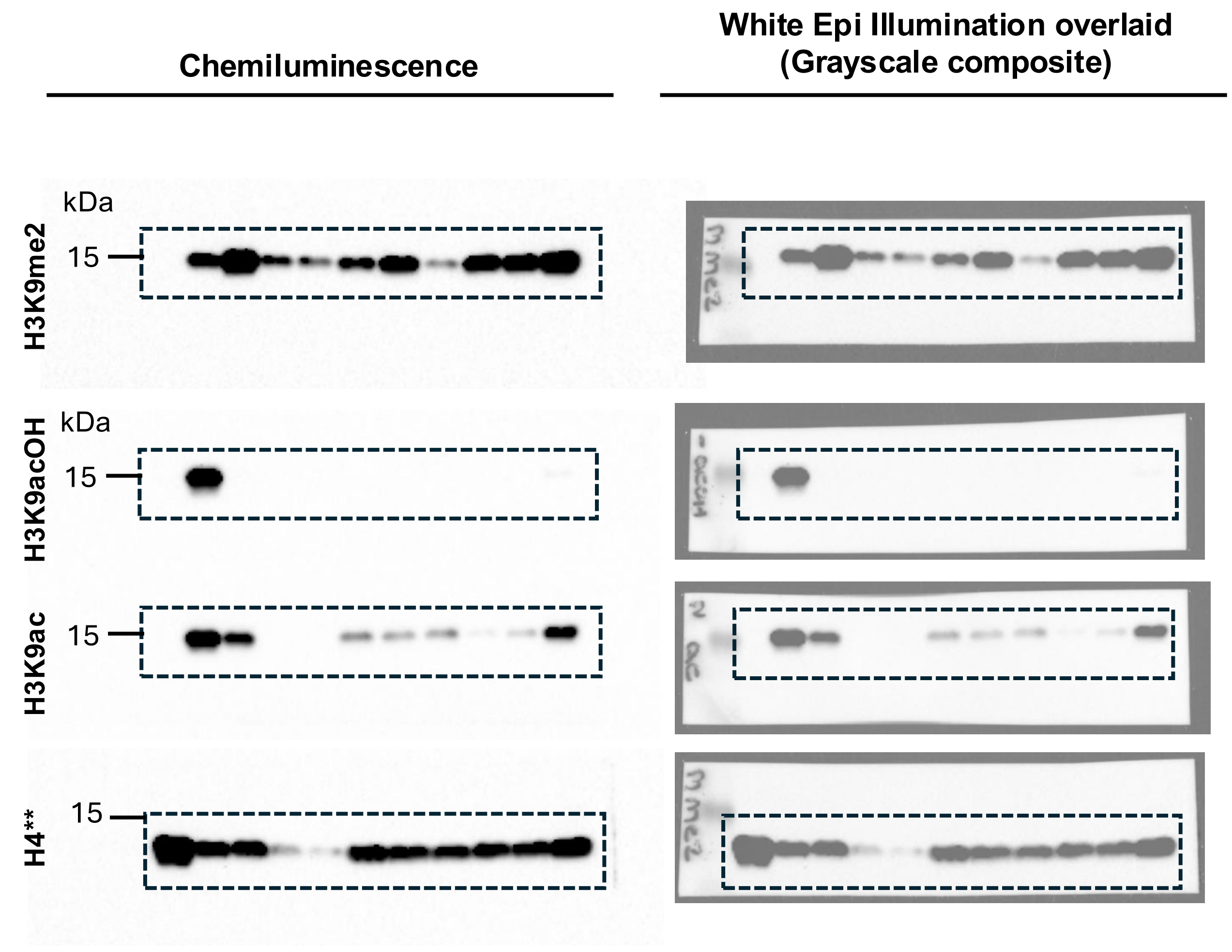

** : H3K9me2 blot reprobed

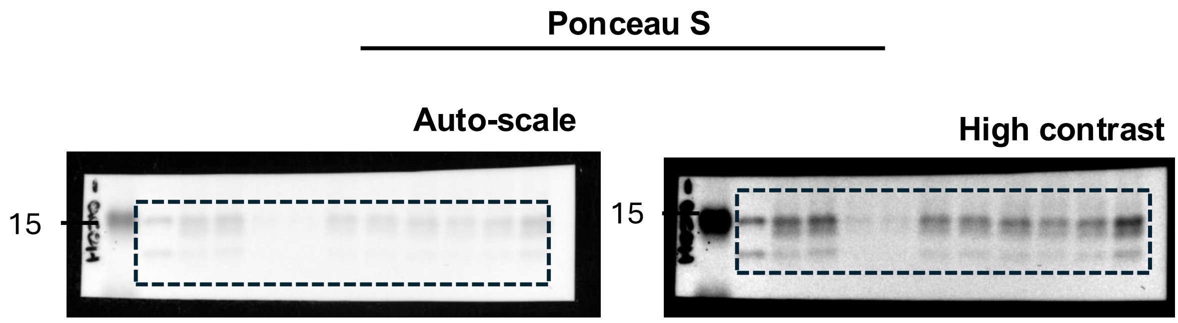

Source Data Fig 3b

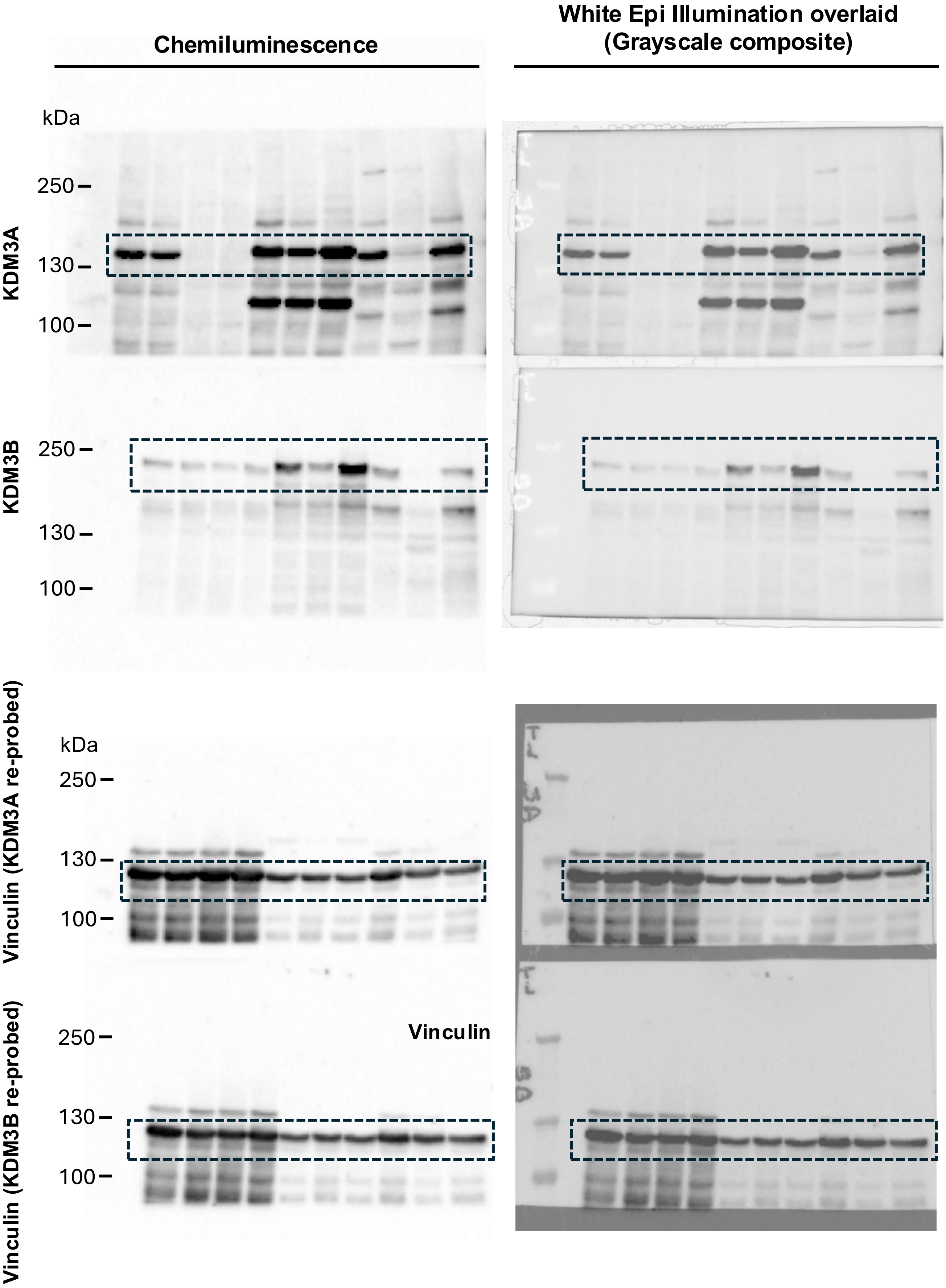

Source Data Fig 3d

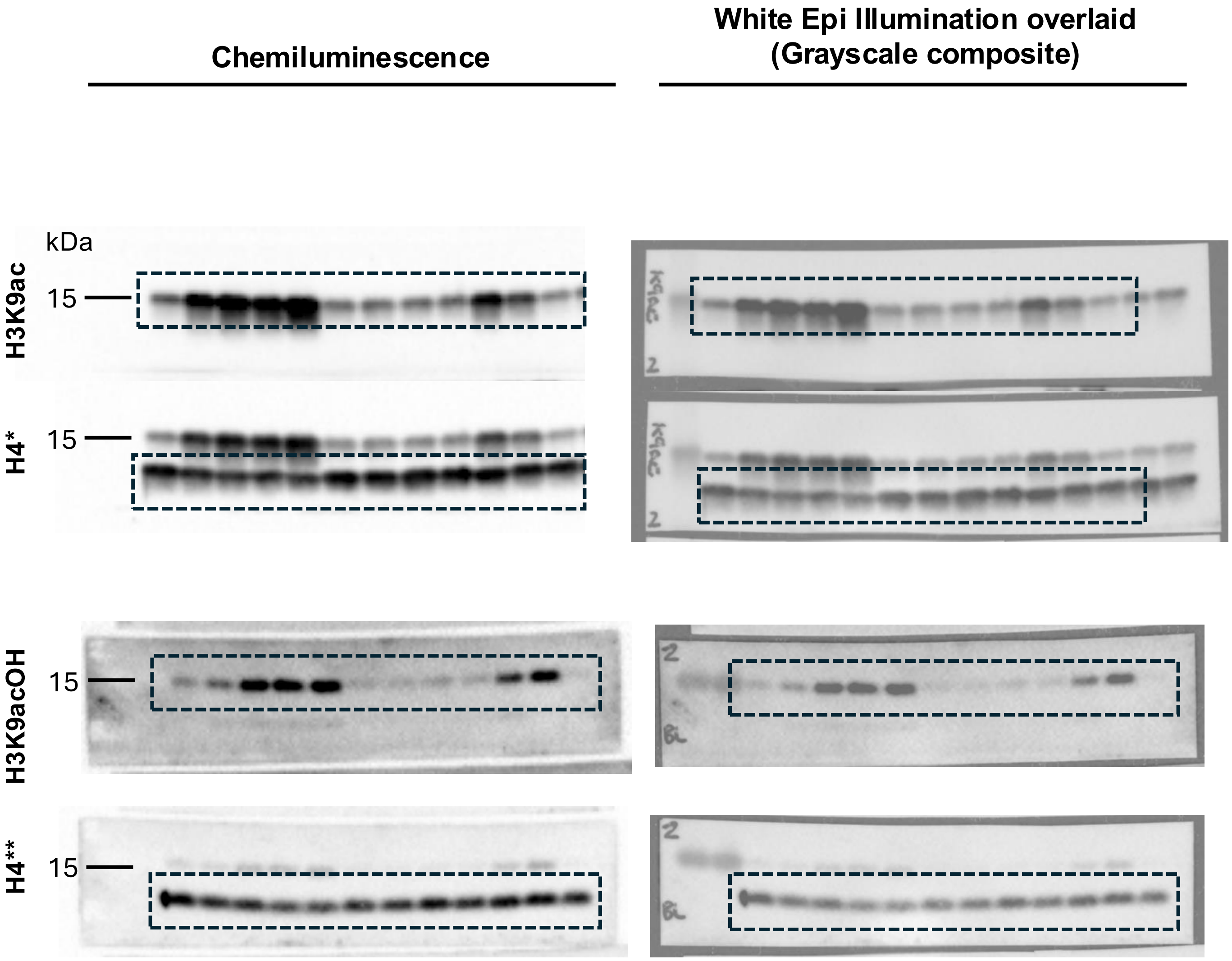

* H3K9ac blot reprobed; **H3K9acOH blot reprobed
