# Supplementary material for: KDM3A catalyses the oxidation of acetyl-lysine to hydroxyacetyl-lysine on histone H3K9
Source: Nat Chem. 2026 Apr 15;18(5):823–34. doi: 10.1038/s41557-026-02112-x (PMC13149330; doi:10.1038/s41557-026-02112-x)

Source Data Extended Fig 7c

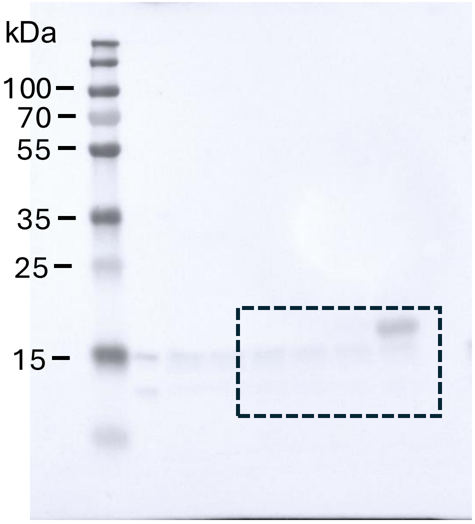

H3K9ac

H4
(H3K9ac blot
reprobed)

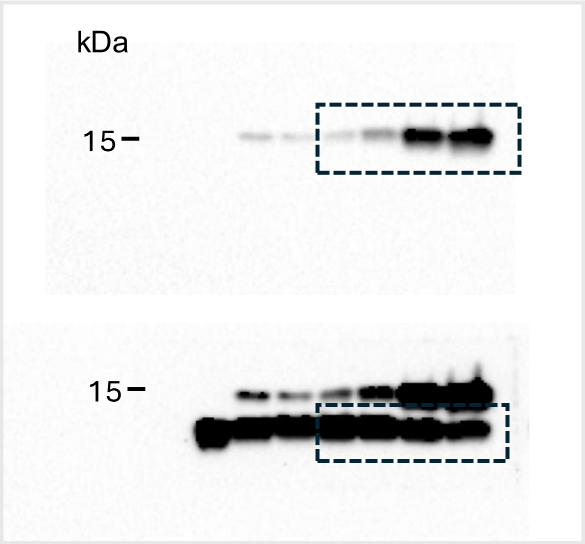

K9acOH

H4
(H3K9acOH blot
reprobed)

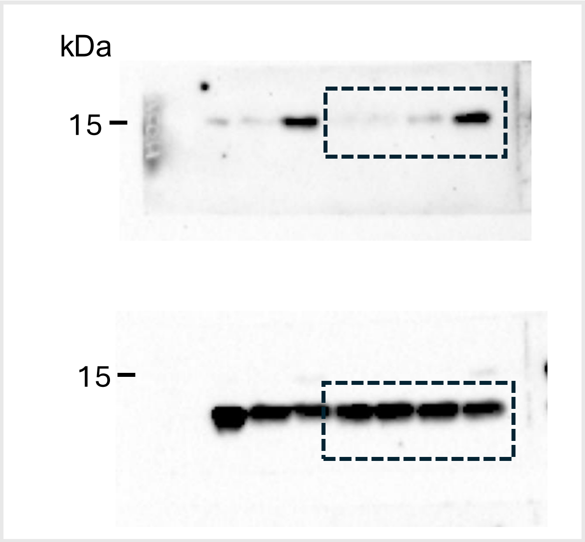

Source Data Extended Fig 7d, e

See Source Data from Fig 2a for H3K9me2, H3K9ac, H3K9acOH, H4 blots

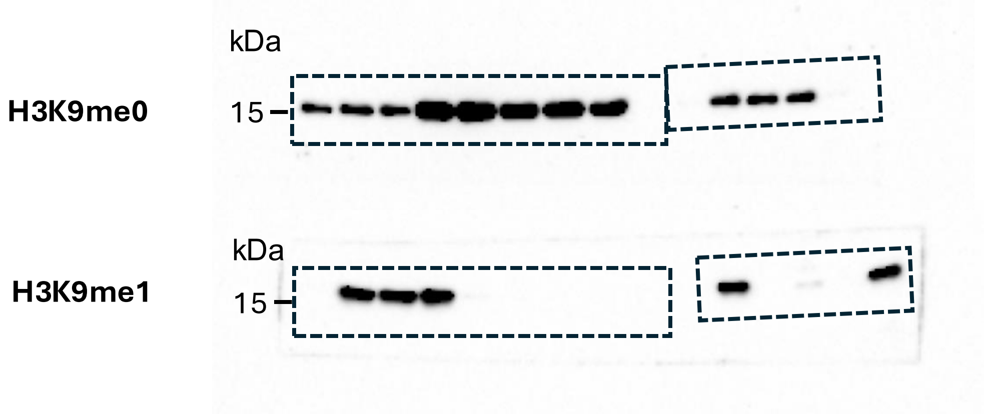

Source Data Extended Fig 7f

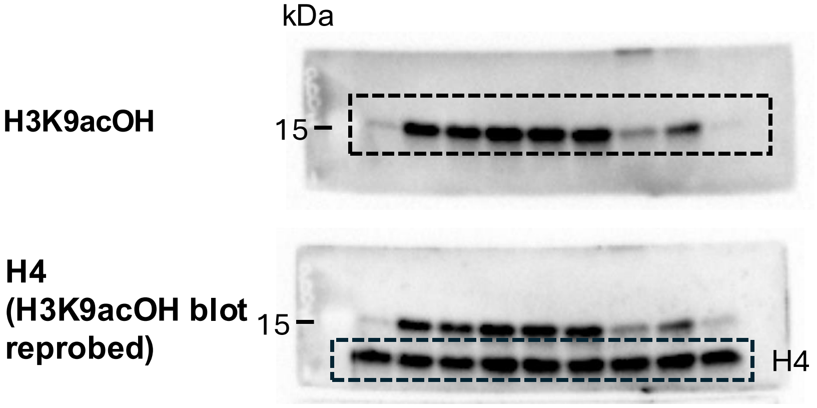

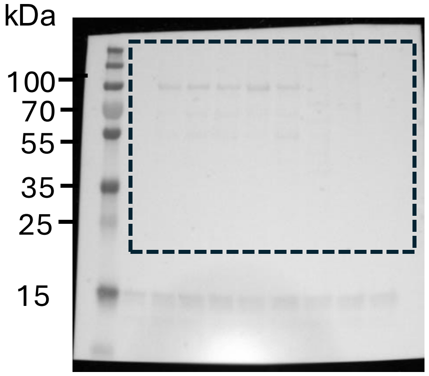

Ponceau S

Source Data Extended Fig 7g

H3K9acOH

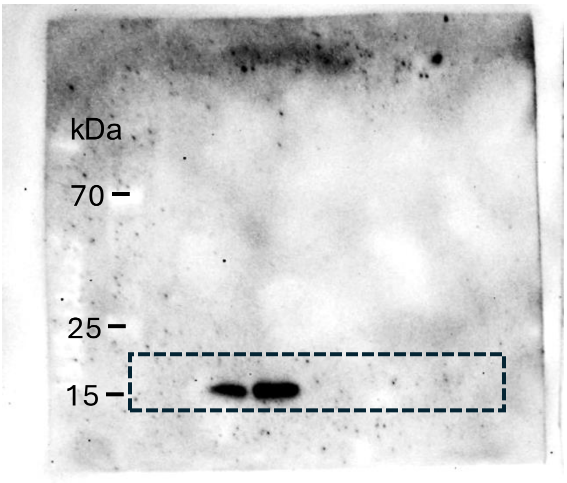

H3K9ac

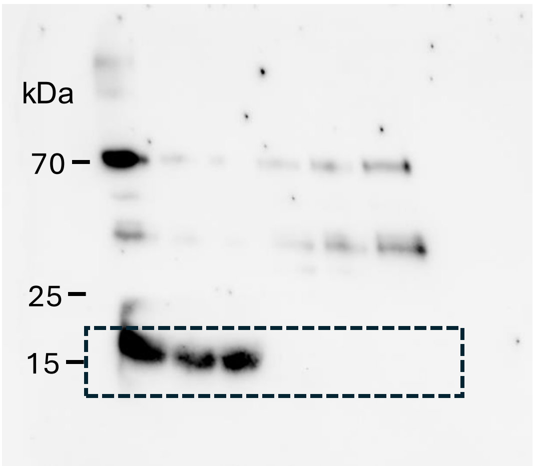

H4

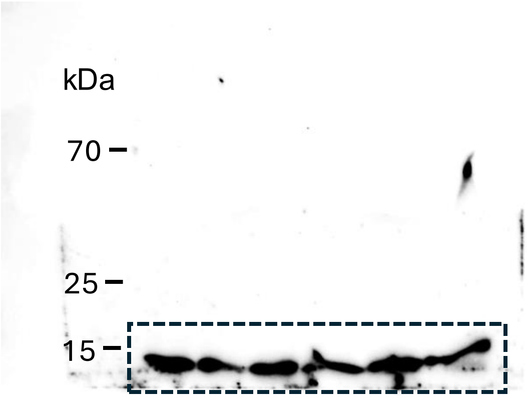

Flag-KDM3A

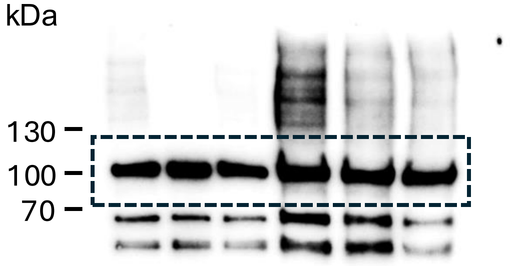

Supplement: Supplementary file 8 — Uncropped western gel blots for Extended Data Fig. 7c–g. [file 41557_2026_2112_MOESM8_ESM.pdf]
